# Asymmetric Monoreduction of α,β-Dicarbonyls to α-Hydroxy Carbonyls by Ene Reductases

**DOI:** 10.1021/acscatal.4c04676

**Published:** 2024-10-09

**Authors:** Allison E. Wolder, Christian M. Heckmann, Peter-Leon Hagedoorn, Diederik J. Opperman, Caroline E. Paul

**Affiliations:** † Biocatalysis section, Department of Biotechnology, Delft University of Technology, van der Maasweg 9, Delft 2629 HZ, the Netherlands; ‡ Department of Microbiology and Biochemistry, University of the Free State, Bloemfontein 9300, South Africa

**Keywords:** biocatalysis, old yellow enzymes, double bond reductases, dicarbonyls, chiral alcohols

## Abstract

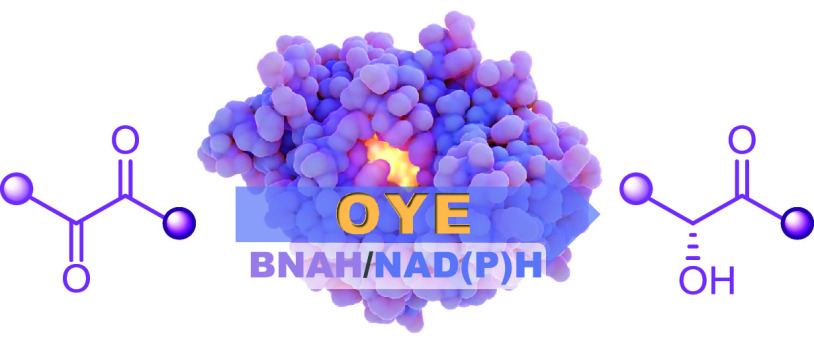

Ene reductases (EREDs) catalyze asymmetric reduction with exquisite chemo-, stereo-, and regioselectivity. Recent discoveries led to unlocking other types of reactivities toward oxime reduction and reductive C–C bond formation. Exploring nontypical reactions can further expand the biocatalytic knowledgebase, and evidence alludes to yet another variant reaction where flavin mononucleotide (FMN)-bound ERs from the old yellow enzyme family (OYE) have unconventional activity with α,β-dicarbonyl substrates. In this study, we demonstrate the nonconventional stereoselective monoreduction of α,β-dicarbonyl to the corresponding chiral hydroxycarbonyl, which are valuable building blocks for asymmetric synthesis. We explored ten α,β-dicarbonyl aliphatic, cyclic, or aromatic compounds and tested their reduction with five OYEs and one nonflavin-dependent double bond reductase (DBR). Only GluER reduced aliphatic α,β-dicarbonyls, with up to 19% conversion of 2,3-hexanedione to 2-hydroxyhexan-3-one with an *R*-selectivity of 83% *ee*. The best substrate was the aromatic α,β-dicarbonyl 1-phenyl-1,2-propanedione, with 91% conversion to phenylacetylcarbinol using OYE3 with *R*-selectivity >99.9% *ee*. Michaelis–Menten kinetics for 1-phenyl-1,2-propanedione with OYE3 gave a turnover *k*_cat_ of 0.71 ± 0.03 s^–1^ and a *K*_m_ of 2.46 ± 0.25 mM. Twenty-four EREDs from multiple classes of OYEs and DBRs were further screened on 1-phenyl-1,2-propanedione, showing that class II OYEs (OYE3-like) have the best overall selectivity and conversion. EPR studies detected no radical signal, whereas NMR studies with deuterium labeling indicate proton incorporation at the benzylic carbonyl carbon from the solvent and not the FMN hydride. A crystal structure of OYE2 with 1.5 Å resolution was obtained, and docking studies showed a productive pose with the substrate.

## Introduction

**Figure 1:**
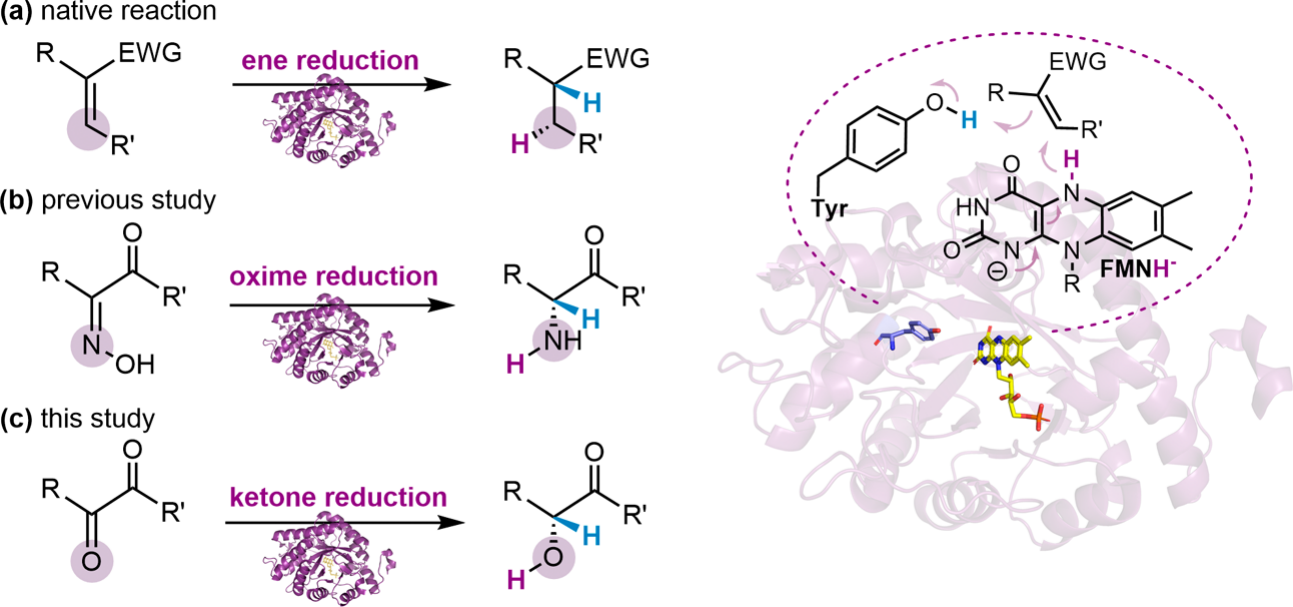
Simplified schematic representation of ERED-catalyzed reductions (left) via hydride transfer from reduced FMN and protonation via a tyrosine (right): (a) the native alkene reduction, (b) the previously observed oxime reduction, and (c) the currently examined vicinal dicarbonyl monoreduction.

**Figure 2:**
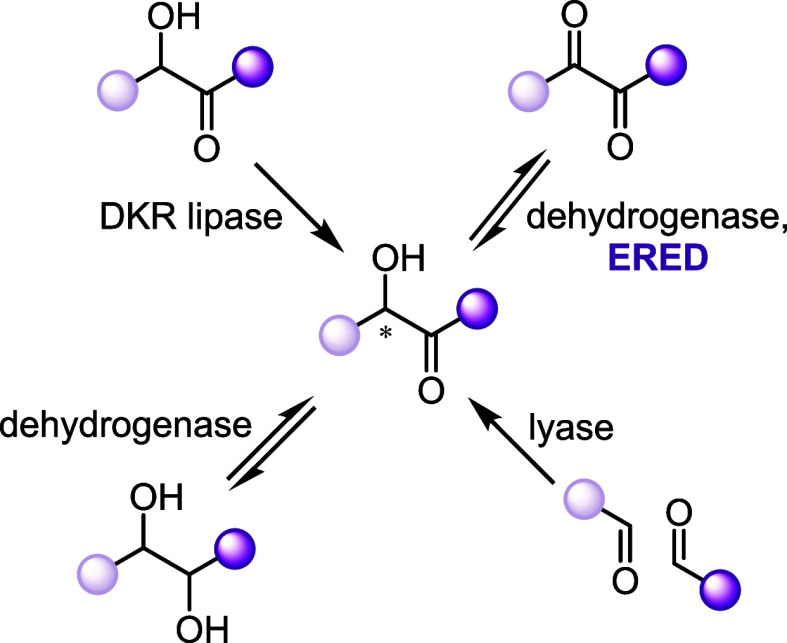
Biocatalytic approaches to produce α-hydroxy carbonyl compounds. DKR (dynamic kinetic resolution) with lipases; reduction by dehydrogenase such as BDH (2,3-butanediol dehydrogenase or acetoin reductase such as BudC) and other ADHs; aldol condensation via lyases including transketolase, MenD (2-succinyl-5-enolpyruvyl-6-hydroxy-3-cyclohexadiene-1-carboxylate synthase), and an oxidase-lyase cascade. Here, we show the state of art for biocatalytic pathways (black) and our added ERED approach. References (12−32).

Discovering new-to-nature and promiscuous enzymatic activities is a strategy to broaden the still modest biocatalytic toolbox for producing fine chemicals. A family of flavin-containing ene-reductases (EREDs) named old yellow enzymes (OYEs) have been studied for close to a century,^1^ yet continue to surprise with its versatile activities.^2^ OYEs are classified into several classes: class I are from plants, cyano-, actino-, and proteobacteria, class II are the classical OYEs from fungi, and class III are similar to class I species but are thermophilic-like OYEs, and so far classes IV–VI are less defined.^3,4^ Typically, OYEs reduce activated alkenes asymmetrically following a bi-bi ping-pong mechanism, in which a reduced nicotinamide cofactor reduces the flavin followed by a flavin hydride attack on the substrate’s β-carbon with a local tyrosine as a proton donor (Figure 1a).^5^ Recently, promiscuous activity for oxime reduction was reported^6^ that exhibits a notably unique mechanism,^7^ in which the oxime is reduced to an amine in a two-step OYE reduction scheme with an imine intermediate (Figure 1b). Looking at other unusual substrates for OYE catalysis, we found that α,β-dicarbonyls were highlighted by two studies. The first one showed a bacterial OYE (GluER from *Gluconobacter oxydans*) had activity on a variety of α,β-dicarbonyls, yet no information about the formed products.^8^ A second study showed OYEs mediate vicinal dicarbonyl reduction;^9^ however, the focus was narrowed to two enzymes from class II, OYE2 and OYE3 from *Saccharomyces cerevisiae* showing kinetic data only, without knowledge of the enzymatic product and enantioselectivity. We wondered whether other OYEs or to a greater extent other EREDs, such as the nonflavin-containing double bond reductases (DBRs), may also selectively reduce vicinal dicarbonyls. The products of such reductions are important templates that contain a chiral α-hydroxy carbonyl for substrate-controlled chemical processes.^10,11^ Although there are several other biocatalytic ways to produce chiral α-hydroxy carbonyl products (Figure 2) such as lipases,^12^ lyases (transketolase,^13^ ThDP-dependent synthase *Ec*MenD,^14^ and alcohol oxidase-lyase cascades),^15^ various dehydrogenases,^16−19^ and Baker’s yeast,^20−30^ the advantage of EREDs over other biocatalysts would be their ability to produce an enantiomerically pure monoproduct without over reduction to a diol.^9^ In this work, we detail the reactivity of EREDs toward the reduction of α,β-dicarbonyl compounds (Figure 1c), as well as provide mechanistic insights.

## Materials and Methods

### Chemicals

All chemicals were purchased from Sigma-Aldrich (Merck, Darmstadt, Germany), TCI Chemicals Europe (Tokyo Chemical Industry, Tokyo, Japan), abcr GmbH (Karlsruhe, Germany), or Alfa Aesar (Thermo Fisher Scientific, Ward Hill, MA, USA) and were used without further purification. The reduced cofactor β-nicotinamide adenine dinucleotide phosphate NADPH (CAS 2646-71-1) was purchased from Oriental Yeast Co., and β-nicotinamide adenine dinucleotide NADH (CAS 606-68-8) was purchased from Prozomix (UK). 1-Benzyl-1,4-dihydronicotinamide BNAH (CAS 952–92–1) and isotopically labeled 1-benzyl-1,4-dihydropyridine-4,4-*d*_*2*_-3-carboxamide ([4-^2^H]-BNAH) were previously synthesized.^38^

### Enzymes

The JM ERED kit EZK002 was gratefully received from Johnson Matthey (Cambridge, UK). All other enzymes were produced in-house. The wild-type enzymes, **GluER**, **OYE2**, **OYE3**, **EBP1**, ***Ts*****OYE**, and variant **OYE3 Y197F**, were recombinantly produced in *E. coli* BL21 Gold(DE3) competent cells harboring the pET-28a(+) vector with an *N*-terminal His-tag (Table S1). A preculture of Lysogeny broth (LB) medium with 50 μg/mL kanamycin was inoculated with a single colony and incubated overnight at 37 °C with shaking at 180 rpm. 1 L of Terrific broth (TB) medium containing 50 μg/mL kanamycin was inoculated with 1% v/v of the preculture and incubated at 37 °C and 180 rpm. When an OD_600_ of 0.5 was reached, the temperature was lowered to 25 °C for induction with 500 μM of isopropyl β-D-1-thiogalactopyranoside (IPTG) and incubated further for 18 h. Cells were harvested by centrifugation (30 min, 4 °C, 18 692 × *g*), washed with buffer (20 mM MOPS-NaOH pH 7, 300 mM NaCl), centrifuged (30 min, 4 °C, 10 000 × *g*), and stored at −20 °C. For cell disruption, the cell pellet was thawed and resuspended with ∼1.5 mL/g cell of lysis buffer (20 mM MOPS-NaOH pH 7, 300 mM NaCl, premixed with an EDTA-free complete protease inhibitor pill, MgCl_2_ (0.5 mM), DNase (0.1 mg/mL), and a spatula tip of lysozyme). The cells were disrupted at 1.35 kbar with a Multi Shot Cell Disruption System at 4 °C and centrifuged (45 min, 4 °C, 20 000 × *g*).

For heat purification (*Ts*OYE), the supernatant was placed in a 50 mL Greiner tube in a heat bath at 70 °C for 90 min and centrifuged (15 min, 4 °C, 4000 × *g*), obtaining a clear yellow supernatant. For IMAC purification (GluER, OYE2, OYE3, and OYE3 Y197F), the supernatant was filtered (0.22 μm) and loaded on a 5 mL HisTrap FF Crude column at 20 °C with loading buffer (20 mM MOPS-NaOH pH 7, 300 mM NaCl, 25 mM imidazole) followed by elution buffer (20 mM MOPS-NaOH pH 7, 300 mM NaCl, 500 mM imidazole). Purified OYE was incubated with 1:1 FMN on ice for 30 min, concentrated with a 10 kDa Amicon filter, and then passed through a PD-10 desalting column with storage buffer (20 mM MOPS-NaOH pH 7, 300 mM NaCl), flash frozen in liquid nitrogen, and stored at −80 °C. OYE concentration was measured by UV for flavin concentration and a BCA assay. Purity was assessed by sodium dodecyl sulfate-polyacrylamide gel electrophoresis (SDS-PAGE, Figure S1). Other enzymes produced can be found in the Supporting Information.

### Bioconversions

Example bioconversion reaction conditions for screening of EREDs with substrates **1–15a**: in a 2 mL plastic safe-lock Eppendorf tube were added 50 mM MOPS-NaOH pH 7 buffer, 11 mM NADPH (1.1 equiv), 5 μM ERED or 2 mg/mL for the JM kit, 10 mM substrate from a 0.5 M DMSO stock (2% v/v final), 6 h, 30 °C, 900 rpm, 0.5 mL volume, unless otherwise specified. The reaction was extracted with 0.5 mL of ethyl acetate (EtOAc), centrifuged (2 min, 13 000 rpm), and the organic phase was separated and dried with MgSO_4_, centrifuged (2 min, 13 000 rpm), and decanted to GC vials for analysis. For normal phase HPLC the reaction was extracted with heptane:isopropanol (IPA) 9:1 with 5 mg of NaCl. For reverse phase HPLC the reaction was quenched with 0.5 mL MeCN, centrifuged (2 min 13 000 rpm), then diluted 1:4 in MeCN, centrifuged (2 min 13 000 rpm) and pipetted into HPLC glass vials for analysis. For NMR the reaction was extracted with CDCl_3_, dried with MgSO_4_, centrifuged (2 min, 13 000 rpm), and pipetted into NMR tubes.

### Analytical Methods

Gas chromatography (GC) was performed on Shimadzu GC-2010 gas chromatographs (Shimadzu corporation, Kyoto, Japan) equipped with a flame ionization detector (FID), and achiral (CP-Sil 8 CB) and chiral (Chirasil-Dex CB and Hydrodex β-TBDM) columns. Products were confirmed by reference standards and GC-MS. Product concentrations were obtained with calibration curve equations using 5 mM tridecane as an internal standard in EtOAc used to extract all compounds. High pressure liquid chromatography (HPLC) was performed on a Shimadzu Prominence (Shimadzu Corporation, Kyoto, Japan) reverse (ARC-18 column) and normal (chiral CHIRALCEL OD and OB-H columns) phase HPLC instrument equipped with an autosampler (SIL-40a) and diode array detector (SPD-M40 DAD). Products were confirmed by reference standards, and concentrations were measured with a calibration curve. Nuclear magnetic (NMR) spectroscopy was carried out on an Agilent 400 MHz (9.4 T) spectrometer operating at 399.67 MHz for ^1^H at 298 K in CDCl_3_. Spectra were interpreted using the software MestReNova (version 12.0.1 by Mestrelab Research S.L.). Electron paramagnetic resonance (EPR) spectra were recorded on a Bruker EMXplus X-band spectrometer equipped with a helium-flow cryostat operating at a temperature of 20K under the following conditions: Microwave frequency, 9.4096 GHz; microwave power, 2 mW; modulation frequency, 100 kHz; modulation amplitude, 10 G; temperature, 20 K. Polarimetry analyses were performed on a PerkinElmer instrument model 343 equipped with a Na/Hal lamp. Specific rotation measurements were carried out at 589 nm at 20 °C, in CDCl_3_.

### Crystallization

OYE2 was crystallized by hanging drop vapor diffusion in 2 μL drops containing equal amounts of protein (OYE2, 6–8 mg/mL) and precipitant (0.1 M sodium citrate, pH 5, 16% (v/v) PEG 10 000). Crystals were soaked in 30% (w/v) glycerol before flash cryocooling in liquid nitrogen. X-ray diffraction data were collected at Diamond Light Source (UK) on beamline i03 (Table S10). Coordinates and structure factors were deposited in the Protein Data Bank (PDB) under accession code 9FH7.

## Results and Discussion

### Asymmetric Bioreduction of α,β-Dicarbonyls

**Figure 3:**
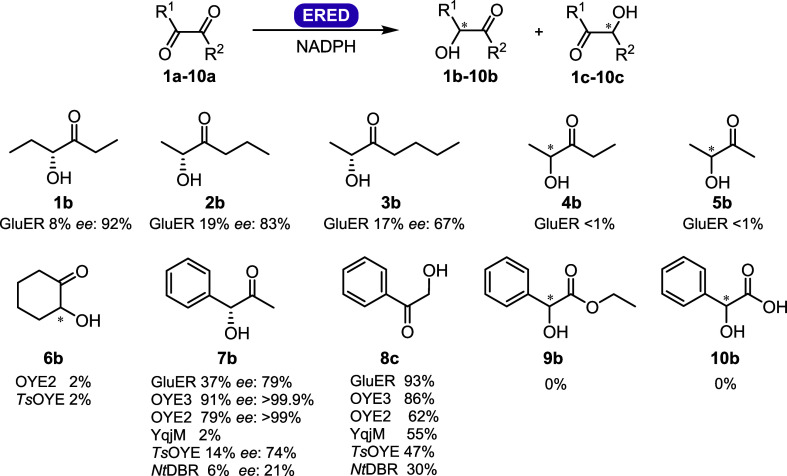
Products **1–10b–c** of ERED-catalyzed selective monoreduction of α,β-dicarbonyl substrates. Conditions: 1.1 eq. NADPH, 10 mM **1a**–**10a**, 2% v/v DMSO, 5 μM ERED, 50 mM MOPS-NaOH pH 7.0, 6 h, 30 °C, 900 rpm, 0.5 mL, average of duplicates. EREDS screened: GluER, *Ts*OYE, OYE3, OYE2, YqjM and *Nt*DBR. Conversions and *ee* values were measured on (chiral) GC. **7b** and **8c** were additionally measured on (chiral) HPLC. Full details are in Table S3. Substrate and product names: **1a** 3,4-hexanedione, **1b** 4-hydroxyhexan-3-one, **2a** 2,3-hexanedione, **2b** 2-hydroxyhexan-3-one, **3a** 2,3-heptanedione, **3b** 2-hydroxyheptan-3-one, **4a** 2,3-pentanedione, **4b** 2-hydroxypentan-3-one, **5a** 2,3-butanedione, **5b** 3-hydroxybutan-2-one, **6a** 1,2-cyclohexanedione, **6b** 2-hydroxycyclohexanone, **7a** 1-phenyl-1,2-propanedione, **7b** phenylacetylcarbinol, **7c** 2-hydroxy-1-phenylpropan-1-one, **8a** phenylglyoxal, **8b** 2-hydroxy-2-phenyl-acetaldehyde, **8c** 2-hydroxyacetophenone, **9a** ethylbenzoylformate, **9b** ethyl 2-hydroxy-2-phenylacetate, **10a** benzoyl formic acid, and **10b** 2-hydroxy-2-phenylacetic acid.

We initially screened a series of ten α,β-dicarbonyl compounds (Figures 3, S12 and Table S3) with six EREDs: GluER (class I), OYE2 and OYE3 (class II), *Ts*OYE from *Thermus scotoductus* and YqjM from *Bacillus subtilis* (class III), as well as a double bond reductase from *Nicotiana tabacum*(*Nt*DBR). All enzymes were produced and purified by heat or affinity chromatography (Table S1), and their activity for cyclohexenone as a model substrate was measured (Table S2). We were surprised to discover glucose dehydrogenase (GDH), used as a cofactor recycling system, effectively reduced α,β-dicarbonyls with varying conversions and enantioselectivity (Figure S13). To ensure that only ERED dicarbonyl reduction activity was measured, a stoichiometric amount of reduced cofactor was used with purified ERED, eliminating the need for a cofactor recycling system.

Linear aliphatic substrates **1a**–**5a** were converted only by GluER into the monoreduced α-hydroxy carbonyl **1b**–**5b** (Figure 3 and Table S3). The two smallest aliphatic substrates **4a** and **5a** showed only traces of conversion as well as considerable mass balance issues (for **4a** see Figure S22). This mass loss may have been due to the use of plastic vials or the volatility of the products. Previous reports showed GluER activity for **4a**,^8^ for which we could show some conversion. A previous study had also shown activity of OYE2 and OYE3 with **4a, 5a,** and **8a**;^9^ however, we could not detect products with **4a** and **5a** due to volatility issues, but could confirm excellent conversions with **8a**. GluER was the most versatile ERED, able to reduce **1a**–**5a**, **7a**, and **8a** where the highest conversion of 19% was found with **2a**. Cyclohexanedione **6a** was not accepted by most of the EREDs screened, with the exception of those for OYE2 and *Ts*OYE, albeit with only traces of conversion and no measurable enantioselectivity (Table S3). Aromatic diketone 1-phenyl-1,2-propanedione **7a** and ketoaldehyde phenylglyoxal **8a** gave a range of low to excellent conversions, whereas ketoester **9a** and ketoacid **10a** were not converted by any of the enzymes studied.

**Figure 4:**
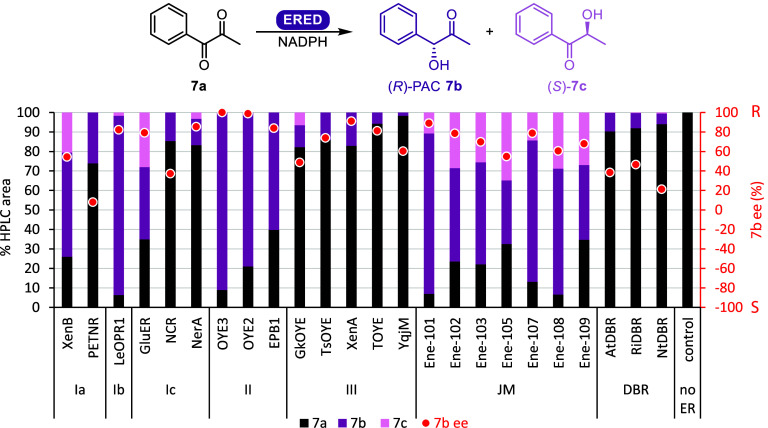
ERED screening of 1-phenyl-1,2-propanedione **7a**. Conditions: 5 μM ERED or 2 mg/mL for the JM kit, 11 mM NADPH, 10 mM 1-phenyl-1,2-propanedione, 6 h, 30 °C, 900 rpm, 2% v/v DMSO, 0.5 mL, 50 mM MOPS-NaOH pH 7.0, average of duplicate experiments measured on HPLC at 210 nm. The OYE classes listed are Ia, Ib, Ic, II, and III.^3^ JM: Johnson Matthey ERED kit EZK002. A scientific color map was used to ensure accurate data representation and inclusivity for readers with color-vision deficiencies.^33^

Since multiple enzymes produced the chiral hydroxyketone product phenylacetylcarbinol (PAC) **7b** (Figure 3), a valuable precursor to norephedrine, the screening was expanded to all our purified in-house EREDs, including the Johnson Matthey (JM) C=C double bond reduction kit containing cell-free extracts (CFE, Figure 4and Table S4). EREDs of class III as well as DBRs showed little conversion, with the exception of JM ENE-105, a DBR, which reached 68% conversion with poor regioselectivity (33% **7b***ee* 55% *R*, 35% **7c***ee* 99% *S*). *Le*OPR1 showed highest levels of product formation with 94% conversion (92% **7b***ee* 82% *R*, 2% **7c***ee* >99.9% *S*). EREDs had **7b** as the major product with (*R*)-stereopreference, and the minor product isomer **7c** with (*S*)-selectivity. Nine of the enzymes produced the single product **7b**; class I (PETNR, NCR), class II (OYE3, OYE2, EPB1), and class III (*Ts*OYE, XenA, *T*OYE, YqjM). The JM lyophilized unpurified enzymes produced both **7b** and isomer **7c**, where perhaps dehydrogenase activity also gave the isomer **7c**. It can equally be noted that *Gk*OYE, which was only heat purified, may also have dehydrogenase activity accounting for some conversion to **7c**. However, XenB, *Le*OPR1, GluER, and NerA, all purified by affinity chromatography, produced both **7b** and **7c**, implying that there are likely two distinct mechanisms to reach these products. In general, OYE3 had the highest enantio- and regio-selectivity combination (>99.9% *ee*, 91% conversion of single product **7b**), an ideal enzyme and substrate combination to further investigate.

We continued to characterize the monoreduction of **7a** with OYE3 by exploring different cofactors, NADPH, NADH, and the synthetic mimic 1-benzyl-1,4-dihydronicotinamide (BNAH) with and without the presence of oxygen (Table 1).^34^ All three cofactors gave a similar high conversion range when done anaerobically of 91–100% (Table 1). A preparative scale bioconversion demonstrated significant conversion >99% with a high selectivity of 97% *ee* (Table 1), with an isolated yield of 33%. BNAH is similar to NADPH in terms of stability in aqueous solutions,^35^ with a half-life of 1.54 h in an aerobic solution (pH 7, 37 °C).^36^ The poorer aerobic conversion with BNAH could be ascribed to degradation, as with oxygen and DMSO removal there was a 1.9-fold higher conversion with OYE3 and BNAH (54% aerobic to 100% anaerobic, Table 1, entry 3). We obtained high conversions anaerobically and overall excellent *ee* > 99.9% (Figure 4; Table 1, entry 2). Finally, we used the native cofactor NADPH and OYE3 to determine Michaelis–Menten kinetics for **7a** and obtained a turnover *k*_cat_ value of 0.71 ± 0.03 s^–1^ (42.6 min^–1^) and a *K*_m_ value of 2.46 ± 0.25 mM, giving an overall catalytic efficiency of 17 mM^–1^min^–1^ (Figure S15).

**Table 1:**
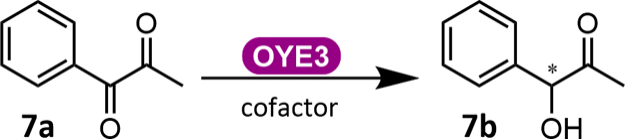
ERED-Catalyzed Monoreduction of 1-Phenyl-1,2-propanedione **7a** to (*R*)-PAC **7b** with Different Cofactors^a^

					Aerobic		Anaerobic	
Entry	Cofactor	[**7a**] (mM)	% DMSO	[OYE] (μM)	**7b** (%)	*ee* (%)	**7b** (%)	*ee* (%)
1	NADPH	10	2	20	91	99 (*R*)	100	99 (*R*)
2	NADH	10	2	20	81	95 (*R*)	100	>99.9 (*R*)
3	BNAH	10	2	20	54	96 (*R*)	100	98 (*R*)
4	BNAH	30	0	20	n.a.	n.a.	91	96 (*R*)
5^b^	BNAH	31	0	30	n.a.	n.a.	99.4	97 (*R*)

a Conditions: 50 mM MOPS-NaOH pH 7, 1.1 eq. cofactor, 6 h, 30 °C, 900 rpm, duplicated experiments, analysed on HPLC.

b Conditions: the same except at the preparative scale, 25 mL volume, single experiment, with an isolated yield of 33%. n.a.: not applicable (not performed).

The second aromatic dicarbonyl, ketoaldehyde phenylglyoxal **8a**, exhibited several unusual characteristics. Instead of major product being α-hydroxy carbonyl **8b** as with the other OYE dicarbonyl reductions, the GC results show monoreduction to the β-hydroxy carbonyl product **8c** (see Table S3 and Figure S38). The expected α-hydroxy carbonyl **8b** has previously been shown to be unstable^8,9^ and may spontaneously form the more stable β-hydroxy carbonyl **8c** especially at the elevated temperatures during GC injection. Thus, observation of **8c** as the only product does not necessarily imply that the regiochemistry of the enzymatic reaction differs. HPLC, LC-MS, and NMR results (see Figures S34–S45) agree with this hypothesis where an aldol reaction product between substrate **8a** and product **8b** is detected, supporting the idea that the OYE enzymes catalyze the reduction of α,β-dicarbonyl compounds toward the highly reactive **8b** as the major product.

To assess whether OYE3 catalyzes other aromatic compounds similar to **7a**, additional substituted substrates were tested: 1-phenylbutan-1,2-dione **11a**, benzil **12a**, 1-(4-(trifluoromethyl)phenyl)propane-1,2-dione **13a**, and 1-(4-methoxyphenyl)propane-1,2-dione **14a** (Table 2). Bulkier substrates **11a** with an additional methyl group and **12a** with a phenyl group, were not converted. *para*-Substituted **13a** with the electron-withdrawing trifluoro group and **14a** with the electron-donating methoxy group afforded 90 and 79% conversion, respectively, with excellent *ee* values of 91 and 99% (Table 2).

**Table 2:**
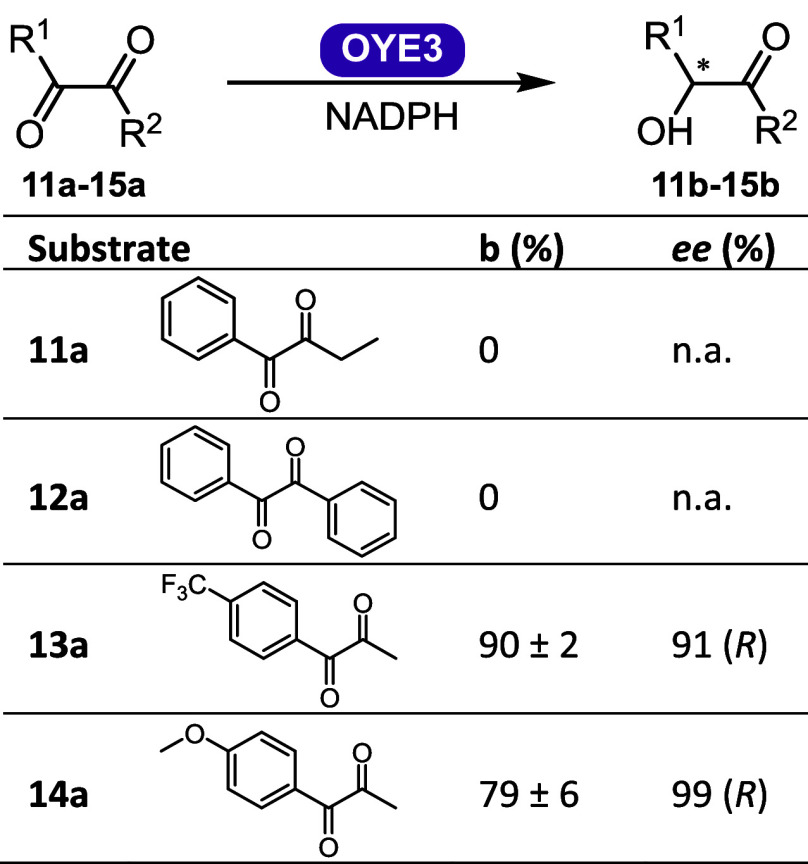
ERED-Catalyzed Monoreduction of Substituted Substrates to (*R*)-Hydroxycarbonyl Product^a^

a n.a.: not applicable.

### Mechanistic and Structural Insights

Previously, ketone reduction of acetophenone derivatives catalyzed by OYEs was reported via a ketyl radical mechanism using a ruthenium photocatalyst.^37^ Therefore, we carried out EPR spectroscopy for the dicarbonyl **7b** monoreduction with OYE3. Both the reaction and blank samples showed no clear radical signal, even at 10-fold higher concentration of OYE3, such that there is no evidence to support a radical mechanism, yet we cannot entirely exclude this possibility (Figure S16). We also screened acetophenone **15a** as a substrate and observed no conversion (see the Figure S53).

**Figure 5:**
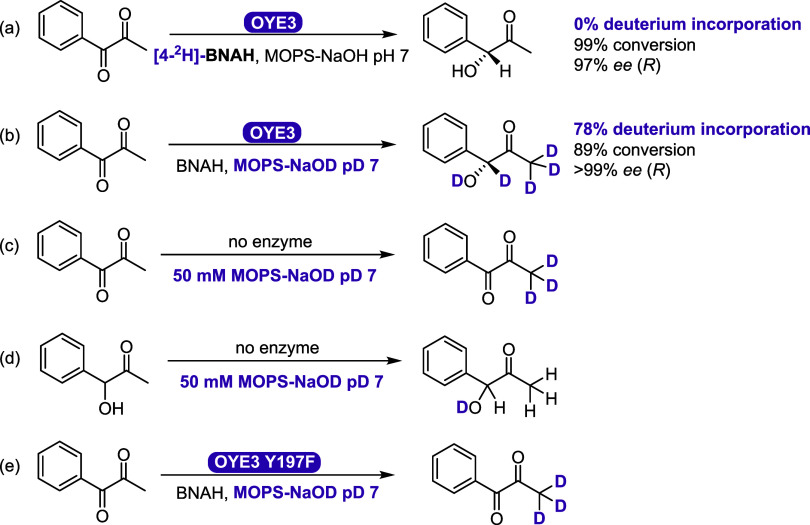
OYE3-catalyzed monoreduction of **7a** to **7b** with (a) dideuterated cofactor [4-^2^H]-BNAH in buffer (50 mM MOPS-NaOH pH 7.0); (b) BNAH in deuterated buffer (50 mM MOPS-NaOD pD 7.0); (c, d) control reactions without enzyme; (e) reaction with OYE3 Y197F. Conditions: 60 μM OYE3, 30 mM 1-phenyl-1,2-propanedione, 30 mM cofactor, 4.5 h at 30 °C, anaerobic.

Further mechanistic studies were conducted by observing the incorporation of deuterium by ^1^H and ^13^C NMR (Figure 5). When isotopically labeled cofactor mimic 1-benzyl-1,4-dihydropyridine-4,4-*d*_*2*_-3-carboxamide ([4-^2^H]-BNAH) was used,^38^ we observed no deuterium incorporation in the product, while still achieving 99% conversion (Figures 5a and S27), thus ruling out a hydride attack on an enol formation followed by protonation at the benzylic position, or an alcohol dehydrogenase-like mechanism with hydride transfer to the carbonyl carbon. Using a deuterated buffer with BNAH, we observed 89% conversion with 78% deuterium incorporation at the benzylic carbon (Figures 5b and S28). These observations, including control reactions in deuterated buffer (Figure 5c,d), are consistent with protonation from the reaction medium at the benzylic carbon and not from the hydride that originates from the cofactor. The OYE3 Y197F mutant showed <5% conversion, which supports a mechanism with direct involvement of Y197.

With these insights, the current proposed mechanism might occur via a concerted hydride transfer to the carbonyl oxygen, followed by protonation of the benzylic α-carbon by the tyrosine, in line with the deuterated product we observed by NMR (Figure S54A). This type of mechanism would align with the previously proposed oxime reduction mechanism with other OYEs,^7^ in which using advanced quantum mechanics/molecular mechanics (QM/MM) simulations,^39^ where the authors propose a hydride transfer to the formally more electronegative nitrogen atom within the CN bond of the imine intermediate. The formation of isomer **7c** with other EREDs can be explained by the formation of an enol, which would be a substrate comparable to that of the C=C double bond of activated alkenes to undergo a hydride attack followed by protonation (Figure S54B).

**Figure 6:**
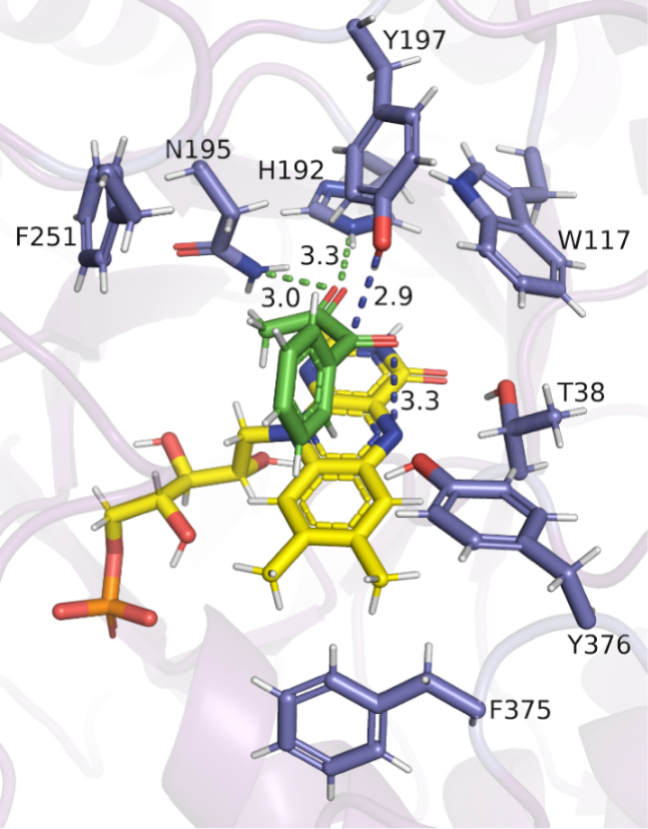
Docking study of **7a** (green) in OYE2 (PDB ID 9FH7). Distances are shown as dashed lines in Å.

Despite numerous attempts at cocrystallization or crystal soaking of OYE2 and OYE3 with **7a**, no interpretable electron density was observed in the active site corresponding to the substrate. A new structure of OYE2 was solved at a resolution of 1.5 Å resolution (Table S9), higher than the previously published 2.45 Å,^40^ and molecular docking was performed with **7a**. Semiflexible dockings were performed whereby **7a** was allowed to sample various conformations, with the condition that only the side chains of amino acids lining the active site of the OYEs were allowed to move (semi-induced fit). Productive binding conformations were considered if one of the carbonyl groups was hydrogen bonded to the active site Asn-His pair. **7a** was docked into OYE2 with the β-carbonyl hydrogen bonded to the Asn-His pair, and the carbonyls in a cis configuration. The benzene ring of **7a** is in an edge-to-face position relative to both FMN and F297 (Figure 6). A similar docking pose was observed for OYE3 (see the Figure S55) whereas *Ts*OYE showed the dicarbonyl groups in a *trans* conformation with either the α- or β-carbonyl hydrogen bonded to the His-His pair (see Figure S56). This would place the ortho carbon of the aromatic ring of **7a** above the N5 of FMN. The dihedral angle between the two carbonyl groups in OYE2 is only ca. 18°, which would suggest a high energy conformation for **7a**. This could, however, be mediated by the hydrogen bonding of the β-carbonyl group to the Asn-His pair. Alternatively, if **7a** were to adopt a lower potential energy by rotation of the intercarbonyl bond, this would place the α-carbonyl oxygen closer to the N5 of FMN.

## Conclusions

EREDs are able to catalyze the asymmetric monoreduction of α,β-dicarbonyl compounds toward α-hydroxy carbonyls. GluER, in particular, was able to reduce aliphatic α,β-dicarbonyl compounds. All tested EREDs from a variety of classes with distinct structural differences were able to reduce aromatic α,β-dicarbonyl compounds. The best results were obtained with class II OYE3 that converted 1-phenyl-1,2-propanedione **7a** to the valuable (*R*)-PAC **7b** (91% conversion *ee* > 99.9%), a precursor to norephedrine. The NMR deuterium labeling mechanistic study carried out with OYE3 showed only the enantiopure (*R*)-**7b** was formed and indicates proton incorporation at the benzylic carbonyl carbon from the solvent, which aligns with the oxime reduction mechanism proposed by Gruber and coworkers.^39^ Some EREDs produced both **7b** and its isomer **7c**, suggesting two mechanisms at work: the second forming a potential enol intermediate. Overall, the monoreduction of α,β-dicarbonyl compounds catalyzed by EREDs showcases a new biocatalytic approach in chemical synthesis to access enantiopure hydroxy carbonyls.

## Supporting Information Available

The production of enzymes, protocols, analytical measurements, dockings, GC chromatograms, and NMR spectra (PDF)
