# Supplementary material for: Asymmetric Monoreduction of α,β-Dicarbonyls to α-Hydroxy Carbonyls by Ene Reductases
Source: ACS Catal. 2024 Oct 9;14(20):15713–20. doi: 10.1021/acscatal.4c04676 (PMC11494505; doi:10.1021/acscatal.4c04676)
Supplement: Supplementary file 1 — cs4c04676_si_001.pdf [file cs4c04676_si_001.pdf]

Supporting Information

Asymmetric monoreduction of α,β-dicarbonyls to α-hydroxy carbonyls by ene reductases

Allison E. Wolder,[a] Christian M. Heckmann,[a] Peter-Leon Hagedoorn,[a] Diederik J. Opperman,[b] Caroline E. Paul[a,*]

[a] *Biocatalysis section, Department of Biotechnology, Delft University of Technology, van der Maasweg 9, 2629 HZ Delft, The Netherlands*
[b] *Department of Microbiology and Biochemistry, University of the Free State, Bloemfontein 9300, South Africa*

Contents

General information 2
Enzyme production and purification 3
Enzyme sequences 9
Enzyme activity 17
Bioconversions 18
Kinetic parameters 20
Analytic methods 20
EPR spectroscopy 20
GC analyses 21
Accuracy of GC measurements for product 7b 22
HPLC analyses 23
HPLC-MS analyses 24
Chromatograms and spectra by substrate 25
Bioconversion of 3,4-hexanedione 1a 25
Bioconversion of 2,3-hexanedione 2a 26
Bioconversion of 2,3-heptanedione 3a 26
Bioconversion of 2,3-pentanedione 4a 27
Bioconversion of 1,2-cyclohexanedione 6a 27
Bioconversion of 1-phenyl-1,2-propanedione 7a 28
NMR spectra of 1-phenyl-1,2-propanedione 7a mechanistic study 29
Preparative scale 1-phenyl-1,2-propanedione 7a 37
Bioconversion of phenylglyoxal 8a 38
LC-MS extracted ion count plot of phenylglyoxal 8a 38
NMR spectra of phenylglyoxal 8a 39
Bioconversion of ethylbenzoylformate 9a 40
Bioconversion of benzoylformic acid 10a 40
Bioconversion of 1-phenylbutan-1,2-dione 11a 41
Bioconversion of benzil 12a 41
Bioconversion of 1-(4-(trifluoromethyl)phenyl)propane-1,2-dione 13a 42
Bioconversion of 1-(4-methoxyphenyl)propane-1,2-dione 14a 43
Bioconversion of 1-acetophenone 15a 44
Proposed mechanisms 44
Crystal structure and docking 45
References 46

General information

All chemicals were purchased from Sigma-Aldrich (Merck, Darmstadt, Germany), TCI Chemicals Europe (Tokyo Chemical Industry, Tokyo, Japan), abcr GmbH (Karlsruhe, Germany) or Alfa Aesar (Thermo Fisher Scientific, Ward Hill, MA, USA) and were used without further purification.

The reduced cofactor β-nicotinamide adenine dinucleotide phosphate NADPH (CAS 2646-71-1) was purchased from Oriental Yeast Co., and β-nicotinamide adenine dinucleotide NADH (CAS 606-68-8) from Prozomix (UK). 1-Benzyl-1,4-dihydronicotinamide BNAH (CAS 952-92-1) and isotopically labelled 1-benzyl-1,4-dihydropyridine-4,4-d_2-3-carboxamide ([4-^{2}H]-BNAH) were previously synthesized.[1] Flavin mononucleotide sodium salt (FMN, CAS 6184-17-4) was purchased from Sigma-Aldrich.

The JM ERED kit EZK002 was gratefully received from Johnson Matthey (Cambridge, UK). All other enzymes were produced in-house. Plasmids were either purchased from BaseClear B.V. (The Netherlands), SynBio Technologies (USA) or received as indicated.

Cells were disrupted in a Multi Shot Cell Disruption System at 4 °C. Immobilized metal (nickel)-affinity chromatography (IMAC) purification was performed on a Next Generation Chromatography (NGC) system from Bio-Rad using 5 mL GE HealthCare HisTrap FF Crude columns unless otherwise specified. Amicon® Ultra 15 mL Centrifugal filters with molecular cut-offs (MWCO) of 10 or 30 kDa were used to concentrate or exchange buffer. Enzyme concentrations were determined with a standard bicinchoninic acid (BCA) assay from Uptima, using known concentrations of bovine serum albumin (BSA) as calibration.

Gas chromatography (GC) was performed on Shimadzu GC-2010 gas chromatographs (Shimadzu corporation, Kyoto, Japan) equipped with a flame ionization detector (FID), and achiral and chiral columns. Products were confirmed by reference standards and GC-MS. Product concentrations were obtained with calibration curve equations using 5 mM tridecane as an internal standard in the EtOAc used to extract all compounds.

High pressure liquid chromatography (HPLC) was performed on a Shimadzu Prominence (Shimadzu corporation, Kyoto, Japan) reverse phase HPLC equipped with an autosampler (SIL-40a) and diode array detector (SPD-M40 DAD). Products were confirmed by reference standards, and concentrations measured with a calibration curve.

Nuclear magnetic (NMR) spectroscopy was carried out on an Agilent 400 MHz (9.4 Tesla) spectrometer operating at 399.67 MHz for ^{1}H at 298 K. Spectra were interpreted using the software MestReNova (version 12.0.1 by Mestrelab Research S.L.).

Electron paramagnetic resonance (EPR) spectra were recorded on a Bruker EMXplus X-band spectrometer equipped with a helium-flow cryostat operating at a temperature of 20K under the following conditions: Microwave frequency, 9.4096 GHz; microwave power, 2 mW; modulation frequency, 100 kHz; modulation amplitude, 10 Gauss; temperature, 20 K.

Polarimetry analyses were performed on a PerkinElmer instrument model 343 equipped with a Na/Hal lamp. Specific rotation measurements were carried out at 589 nm at 20 °C, in $CDCl_3$.

Enzyme production and purification

Table S1. List of enzymes produced for this study.

ERED	source organism	UniProt	vector	*E. coli* host strain	His-tag	antibiotic
GluER	*Gluconobacter oxydans* 621H	Q5FTL6	pET-28a(+)	BL21 Gold(DE3)	*N*-term	Kan
*Le*OPR1	*Lycopersicon esculentum (Solanum lycopersicum)*	Q9XG54	pET-21a(+)	BL21 Gold(DE3)	*C*-term	Amp
NCR	*Zymomonas mobilis* subsp. *mobilis* ATCC 31821/ZM4/CP4	Q5NLA1	pET-22b(+)	BL21 Gold(DE3)	*C*-term	Amp
NerA	*Rhizobium radiobacter*	O31246	pET-21a(+)	BL21 Gold(DE3)	*C*-term	Amp
PETNR	*Enterobacter cloacae*	P71278	pET-21a(+)	BL21 Gold(DE3)	*C*-term	Amp
XenB	*Pseudomonas fluorescens*	Q9RPM1	pET-21a(+)	BL21 Gold(DE3)	*C*-term	Amp
OYE2	*Saccharomyces cerevisiae* ATCC 204508/S288c	Q03558	pET-28a(+)	BL21 Gold(DE3)	*N*-term	Kan
OYE3	*Saccharomyces cerevisiae* ATCC 204508/S288c	P41816	pET-28a(+)	BL21 Gold(DE3)	*N*-term	Kan
OYE3 Y197F	*Saccharomyces cerevisiae*	P41816	pET-28a(+)	BL21 Gold(DE3)	*N*-term	Kan
EBP1	*Candida albicans*	P43084	pET-28a(+)	BL21 Gold(DE3)	*N*-term	Kan
*Gk*OYE	*Geobacillus kaustophilus* HTA426	Q5KXG9	pET-28a(+)	BL21(DE3)	*N*-term	Kan
*T*OYE	*Thermoanaerobacter pseudethanolicus* ATCC 33223/39E	B0KAH1	pET-21a(+)	BL21 Gold(DE3)	*C*-term	Amp
*Ts*OYE	*Thermus scotoductus* SA-01	B0JDW3	pET-28a(+)	BL21 Gold(DE3)	*N*-term	Kan
XenA	*Pseudomonas putida*	Q9R9V9	pET-28a(+)	BL21 Gold(DE3)	*N*-term	Kan
YqjM	*Bacillus subtilis* 168	P54550	pET-28a(+)	BL21(DE3)	*N*-term	Kan
*Nt*DBR	*Nicotiana tabacum*	Q9SLN8	pET-28a(+)	BL21(DE3)pLysS	*N*-term	Kan + Cam
*At*DBR	*Arabidopsis thaliana*	Q39172	pET-28a(+)	BL21(DE3)pLysS	*N*-term	Kan + Cam
*Ri*DBR	*Rubus idaeus*	G1FCG0	pET-28a(+)	BL21(DE3)pLysS	*N*-term	Kan + Cam
*Bs*GDH E170K/Q252L	*Bacillus subtilis* 168	P12310	pET-28a(+)	BL21 Gold(DE3)	*N*-term	Kan

Kan = kanamycin; Amp = ampicillin; Cam = chloramphenicol.

The wild-type enzymes **GluER**, **OYE2**, **OYE3**, **EBP1**, ***Ts*OYE**, and variant **OYE3 Y197F**, were recombinantly produced in *E. coli* BL21 Gold(DE3) competent cells harboring the pET-28a(+) vector with a *N*-terminal His-tag (**Table S1**). A pre-culture of Luria broth (LB) medium with 50 µg/mL kanamycin was inoculated with a single colony and incubated overnight at 37 °C with shaking at 180 rpm. 1 L of Terrific broth (TB) medium containing 50 µg/mL kanamycin was inoculated with 1% v/v of the pre-culture and incubated at 37 °C and 180 rpm. When an OD_{600} of 0.5 was reached, the temperature was lowered to 25 °C for induction with 500 µM of isopropyl β-D-1-thiogalactopyranoside (IPTG) and incubated further for 18 h. Cells were harvested by centrifugation (30 min, 4 °C, 18,692 × *g*), washed with buffer (20 mM MOPS-NaOH pH 7, 300 mM NaCl), centrifuged (30 min, 4 °C, 10,000 × *g*) and stored at -20 °C. For cell disruption, the cell pellet was thawed and re-suspended with ~1.5 mL/g cell of lysis buffer (20 mM MOPS-NaOH pH 7, 300 mM NaCl, premixed with an EDTA-free complete protease inhibitor pill, $MgCl_2$ (0.5 mM), DNase (0.1 mg/mL) and a spatula tip of lysozyme). The cells were disrupted at 1.35 kbar with a Multi Shot Cell Disruption System at 4 °C and centrifuged (45 min, 4 °C, 20,000 × *g*).

For heat purification (*Ts*OYE), the supernatant was placed in a 50 mL Greiner tube in a heat bath at 70 °C for 90 min and centrifuged (15 min, 4 °C, 4000 × *g*), obtaining a clear yellow supernatant. For IMAC purification (GluER, OYE2, OYE3, OYE3 Y197F), the supernatant was filtered (0.22 µm), loaded on a 5 mL HisTrap FF Crude column at 20 °C with loading buffer (20 mM MOPS-NaOH pH 7, 300 mM NaCl, 25 mM imidazole) followed by elution buffer (20 mM MOPS-NaOH pH 7, 300 mM NaCl, 500 mM imidazole). Purified OYE was incubated with 1:1 FMN on ice for 30 min, concentrated with a 10 kDa Amicon filter then passed through a PD-10 desalting column with storage buffer (20 mM MOPS-NaOH pH 7, 300 mM NaCl), flash frozen in liquid nitrogen and stored at -80 °C. OYE concentration was measured by UV for flavin concentration and a BCA assay. Purity was assessed by sodium dodecyl sulfate-polyacrylamide gel electrophoresis (SDS-PAGE, **Figure S1**).

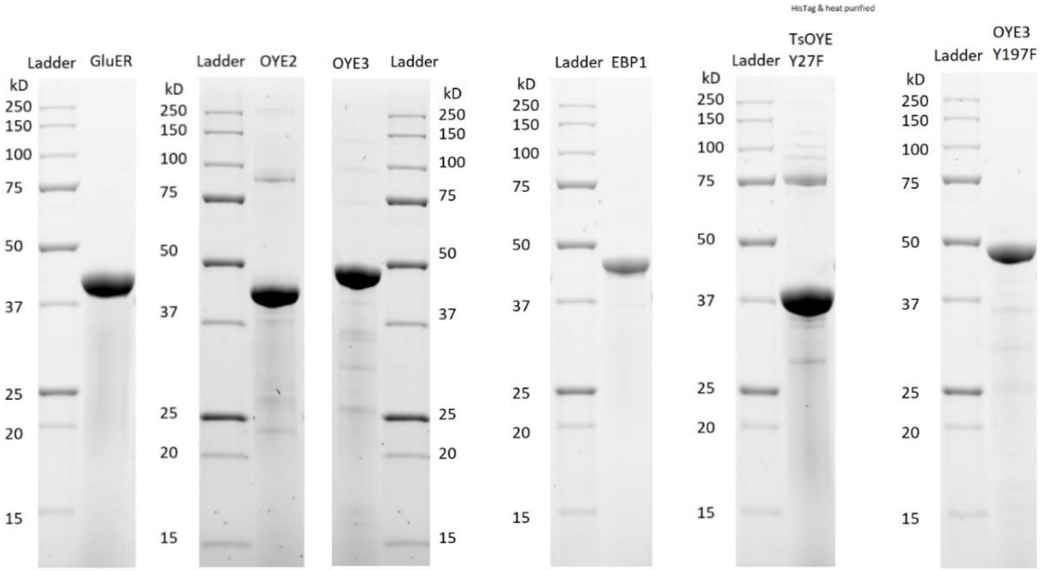

Figure S1. SDS-PAGE gels of purified enzymes GluER, OYE2, OYE3, EBP1, *Ts*OYE Y27F, and OYE3 Y197F

***Gk*OYE** was recombinantly produced in *E. coli* BL21(DE3) competent cells harboring the plasmid pET-28a(+)-*N-his-gkoye* as above with the following variations: when an OD_{600} of 0.7 was reached, the temperature was decreased to 30 °C for induction with 500 µM of IPTG and further incubated for 18 h. Cells were harvested by centrifugation (20 min, 4 °C, 17,024 × *g*), washed with buffer (20 mM MOPS-NaOH pH 7.5), centrifuged (30 min, 4 °C, 3,200 × *g*) and stored at -20 °C. The cells were thawed and re-suspended with 2 mL/g cell of lysis buffer, disrupted and centrifuged (45 min, 4 °C, 11,963 × *g*).
For heat purification, the supernatant was placed in a 50 mL Greiner tube in a heat bath at 55 °C for 30 min and centrifuged (20 min, 4 °C, 11,963 × *g*) obtaining a clear yellow supernatant. The supernatant was filtered (0.22 µm), incubated with 1:1 FMN overnight at 4 °C, then passed through a PD-10 desalting column with MOPS-NaOH buffer (20 mM, pH 7.5). The enzyme stock was concentrated with a 30 kDa Amicon filter, then flash frozen in liquid nitrogen and stored at -80 °C (**Figure S2**).

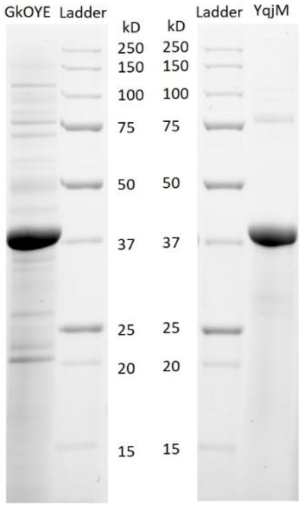

Figure S2. SDS-PAGE gels of purified enzymes *Gk*OYE and YqjM.

YqjM was recombinantly produced in *E. coli* BL21(DE3) competent cells harboring the plasmid pET-28a(+)-*N-his-yqjm*. 1% v/v pre-culture was added in autoinduction ZYM-5052 media with 50 µg/mL kanamycin and grown overnight at 37 °C and 180 rpm. Cells were harvested by centrifugation (4,500 × *g*, 15 min, 4 °C), washed with buffer (20 mM KPi pH 6.5) and centrifuged (10,000 × *g*, 15 min, 4 °C). The cell pellet was resuspended (1:4 ratio) in lysis buffer (20 mM KPi pH 6.5, 30 mM imidazole, premixed with an EDTA-free complete protease inhibitor pill, $MgCl_2$ (0.5 mM), DNase (0.1 mg/mL)).

Cells were disrupted and centrifuged (20,000 × *g*, 30 min at 4 °C). For IMAC purification, the supernatant was loaded to a 5 mL column, washed with buffer (20 mM KPi pH 6.5, 30 mM imidazole) and eluted (20 mM KPi pH 6.5, 250 mM imidazole) in a 0-100% gradient over seven column volumes (CV). Collected fractions were concentrated with a 10 kDa Amicon filter and passed through a PD-10 desalting column with 20 mM KPi pH 6.5. The enzyme was flash frozen in liquid nitrogen and stored at -80 °C (**Figure S2**).

XenA was recombinantly produced in *E. coli* BL21 Gold(DE3) competent cells harboring the plasmid pET-28a(+)-*N-his-xena* (purchased from BaseClear). 1% v/v of pre-culture inoculated 0.5 L TB autoinduction medium containing 50 µg/mL kanamycin, 5 g/L lactose and 0.5 g/L glucose, grown for 2 h at 37 °C and 180 rpm, then lowered to 25 °C for 20 h. Cells were harvested by centrifugation (10 min, 4 °C, 5000 × *g*), washed with buffer (50 mM KPi pH 8), centrifuged in 50 mL Greiner tubes (10 min, 4 °C, 5000 × *g*) and stored at -20 °C (7.6 g/500 mL wet cell pellet). Cells were resuspended (1:3 ratio) in lysis buffer (50 mM KPi pH 8, 10 mM imidazole, 300 mM NaCl, EDTA-free cOmplete^{TM} protease inhibitor cocktail pill, $MgCl_2$ (0.5 mM), DNase (0.1 mg/mL), 0.2 mM FMN). The cells were disrupted and centrifuged (1 h, 4 °C, 18,000 × *g*). The supernatant (~10 mL) was filtered (0.22 µm) and loaded on a 5 mL column equilibrated with buffer (50 mM KPi pH 8, 10 mM imidazole, 300 mM NaCl), washed (50 mM KPi pH 8, 20 mM imidazole, 300 mM NaCl) and eluted (50 mM KPi pH 8, 300 mM imidazole, 300 mM NaCl). The eluted enzyme fractions were passed through a PD-10 desalting column with 20 mM MOPS-NaOH pH 7, flash frozen in liquid nitrogen and stored at -80 °C (**Figure S3**).

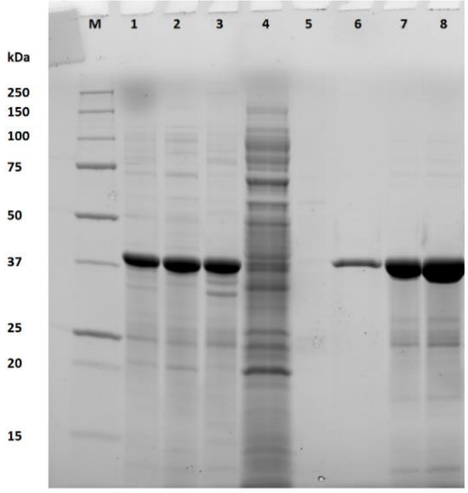

Figure S3. SDS-PAGE gel of XenA (40.7 kDa) production and purification. **M:** protein marker, **1:** harvested pellet, **2:** insoluble fraction, **3:** CFE, **4:** loading waste, **5:** washing waste, **6:** elution not kept, **7:** 0.05 g/L XenA, **8:** 0.10 g/L XenA.

***T*OYE**, **NerA**, ***Le*OPR1**, **XenB** and **PETNR** were each recombinantly produced in a pET-21a(+) vector with a *C*-terminal His-tag, given graciously by Prof. N. Scrutton of the Manchester Institute of Biotechnology (UK). The plasmids were transformed into *E. coli* BL21 Gold(DE3) competent cells. A single colony was picked for the 1% v/v pre-culture in LB medium with 100 µg/mL ampicillin and grown overnight at 37 °C and 180 rpm. 0.5 L TB autoinduction medium (in 2 L shake flasks) containing 100 µg/mL ampicillin, 5 g/L lactose and 0.5 g/L glucose was inoculated with 1% v/v pre-culture and grown for 2 h at 37 °C and 180 rpm, then at 25 °C for 20 h. Cells were harvested by centrifugation (10 min, 4 °C, 5000 × *g*), washed with buffer (50 mM KPi pH 8), centrifuged (4000 × *g*, 4 °C for 10 min) in 50 mL Greiner tubes and stored at -20 °C (8 g/0.5 L of wet cell pellet). Cells were resuspended (1:3 ratio) in lysis buffer (50 mM KPi pH 8, 10 mM imidazole, 300 mM NaCl, EDTA-free cOmplete protease inhibitor cocktail pill, $MgCl_2$ (0.5 mM), DNase (0.1 mg/mL), 0.2 mM FMN). The cells were disrupted and centrifuged (1 h, 4 °C, 18,000 × *g*). The supernatant was filtered (0.22 µm), loaded on a 5 mL column equilibrated with buffer (50 mM KPi pH 8, 10 mM imidazole, 300 mM of NaCl), washed (50 mM KPi pH 8, 20 mM imidazole, 300 mM of NaCl), and eluted (50 mM KPi pH 8, 300 mM imidazole, 300 mM of NaCl). The

eluted enzyme was passed through a PD-10 desalting column with 20 mM MOPS-NaOH buffer pH 7, flash frozen in liquid nitrogen and stored at -80 °C (**Figure S4-8**).

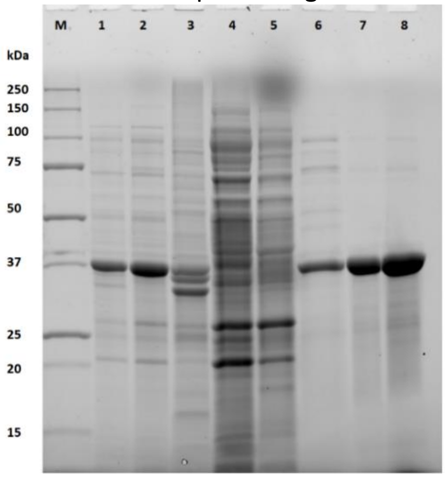

Figure S4. SDS-PAGE gel of *T*OYE (38.6 kDa) production and purification. **M:** protein marker, **1:** harvested pellet, **2:** insoluble fraction, **3:** CFE, **4:** loading waste, **5:** washing waste, **6:** elution not kept, **7:** 0.05 g/L *T*OYE, **8:** 0.10 g/L *T*OYE.

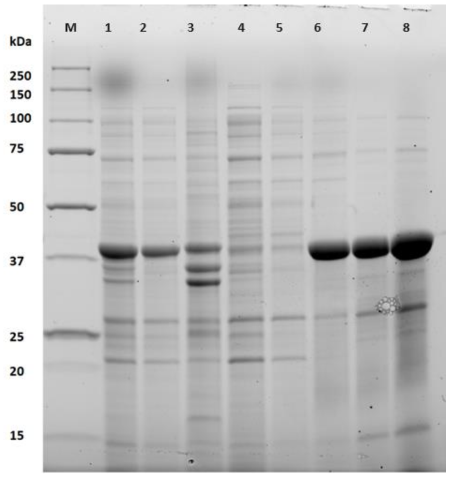

Figure S5. SDS-PAGE gel of NerA (40.8 kDa) production and purification. **M:** protein marker, **1:** harvested pellet, **2:** insoluble fraction, **3:** CFE, **4:** loading waste, **5:** washing waste, **6:** elution, **7:** 0.05 g/L NerA, **8:** 0.10 g/L NerA.

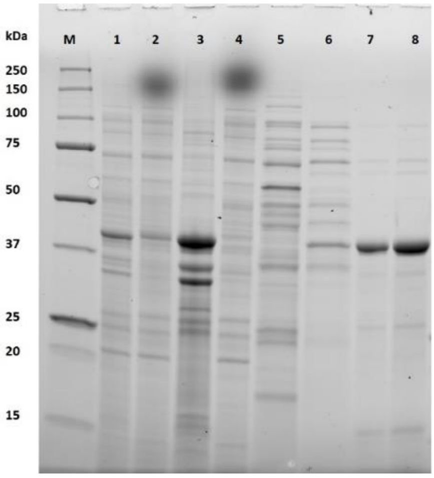

Figure S6. SDS-PAGE gel of *Le*OPR1 (43.5 kDa) production and purification. **M:** marker, **1:** harvested pellet, **2:** insoluble fraction, **3:** CFE, **4:** loading waste, **5:** washing waste, **6:** elution not kept, **7:** 0.05 g/L *Le*OPR1, **8:** 0.10 g/L *Le*OPR1.

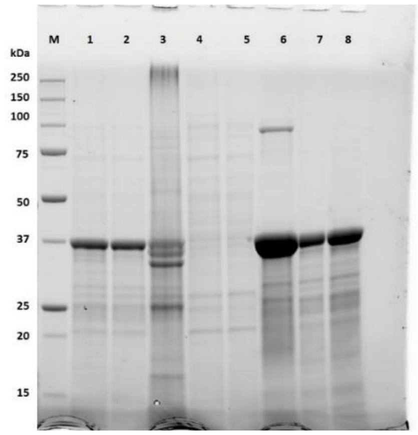

Figure S7. SDS-PAGE gel of XenB (38.65 kDa) production and purification. **M:** protein marker, **1:** harvested pellet, **2:** insoluble fraction, **3:** CFE, **4:** loading waste, **5:** washing waste, **6:** elution not kept, **7:** 0.05 g/L XenB, **8:** 0.10 g/L XenB.

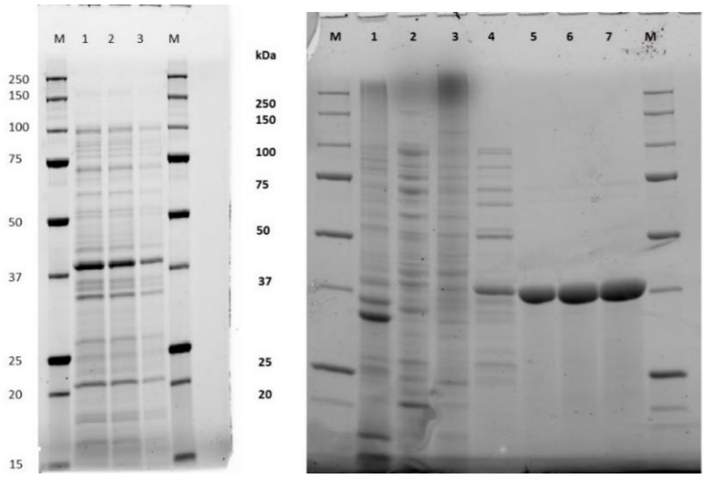

Figure S8. SDS-PAGE gel of PETNR (40.3 kDa) production and purification. **Left:** Harvested cell pellet where **M:** protein marker, **1:** ~0.075 OD_{600}, **2:** ~0.05 OD_{600}, **3:** ~0.025 OD_{600}. **Right:** Purification where **M:** protein marker, **1:** insoluble fraction, **2:** flowthrough while loading, **3:** flowthrough with washing step, **4:** elution not kept, **5-7:** elution of enzyme after PD-10, **5:** 0.05 g/L, **6:** 0.075 g/L, **7:** 0.1 g/L.

NCR was recombinantly produced in *E. coli* BL21 Gold(DE3) competent cells harboring a pET-22b vector (NdeI/XhoI) with a *C*-terminal His-tag. A pre-culture (15 mL) of LB medium containing ampicillin (100 µg/mL) was inoculated with a single colony and incubated overnight at 37 °C, 180 rpm. TB medium (600 mL in a 2 L baffled shake-flask) containing ampicillin (100 µg/mL), lactose monohydrate (5 g/L), and glucose (0.5 g/L) was inoculated with the pre-culture and incubated at 37 °C, 180 rpm (1 inch throw) for 2 h, followed by 25 °C, 180 rpm (1 inch throw) for 21 h. Cells were harvested centrifugation at 4500 × *g*, 4 °C, 20 min, washed with deionized water, and pelleted again (3220 × *g*, 4 °C, 20 min). The cell pellet was stored at -20 °C (12.4 g wet cell pellet, 20.6 g/L).
Cells were resuspended (with the aid of brief sonication) in buffer A (MOPS-NaOH (20 mM), NaCl (300 mM), imidazole (25 mM), pH 7.5) in approx. 3:1 v:w ratio. A spatula tip of both $MgCl_2$ and DNase was added, followed by FMN (approx. 0.2 mg/mL). The cells were disrupted (two cycles at 21 kpsi), and the lysate was centrifuged for 1 h (48,000 × *g*, 4 °C) and the supernatant was filtered (0.45 µm then 0.22 µm). IMAC purification was carried out on a 5 mL His-Trap FF crude column. After loading the sample, the column was washed with buffer A (3 CV), followed by 10% buffer B (20 mM MOPS-NaOH, 300 mM NaCl, 300 mM imidazole, pH 7.5; 2 CV). Leaching of NCR was observed during this washing. The protein was then eluted using 100% buffer B in fractions of 2.5 mL. The 10% B and 100% B fractions were combined (after verifying purity by SDS-PAGE), and dialysed overnight against storage buffer (20 mM MOPS-NaOH, pH 7.5) at 5 °C; the buffer was renewed after the first 3 h of dialysis. The sample was concentrated using a 20 kDa Amicon filter and protein concentration was estimated using the extinction coefficient of the bound FMN (464 nm, 10.5 $mM^{-1}cm^{-1}$). The concentration was 864 µM

(35 mg/mL, 8 approx. 220 mg/L culture); 70 nmol aliquots were flash frozen in liquid nitrogen and stored at -80 °C until use (Figure S9).

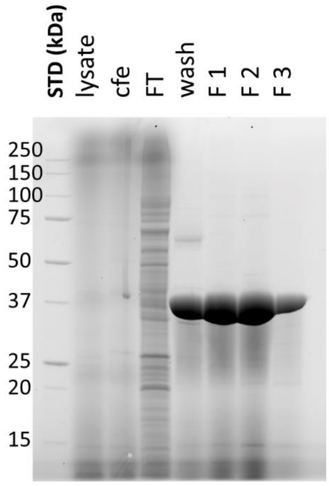

Figure S9. SDS-PAGE gel of NCR production and purification. CFE: cell free extract, FT: flow through, F1-3 fraction eluted with 100% B.

***Nt*DBR**, ***At*DBR** and ***Ri*DBR** were recombinantly produced in *E. coli* BL21(DE3)pLysS with the vector pET-28a(+). Pre-culture of LB medium (50 µg/mL kanamycin for *Nt*DBR, 50 µg/mL kanamycin and 50 µg/mL chloramphenicol for *Ri*DBR and *At*DBR) was inoculated with a single colony and incubated overnight at 37 °C and 180 rpm. 1% v/v pre-culture in TB medium (50 µg/mL of kanamycin for *Nt*DBR, and 50 µg/mL of kanamycin and 50 µg/mL of chloramphenicol for *Ri*DBR and *At*DBR) was incubated at 37 °C and 180 rpm. When an OD_{600} of 0.6 was reached (~3 h), 400 µM of IPTG (*Nt*DBR) and 500 µM of IPTG (*Ri*DBR, *At*DBR) was added while cells were on ice. After induction, cultures were incubated for 18 h at 18 °C and 180 rpm. Cells were harvested by centrifugation (15 min, 4 °C, 4,500 × *g*). For cell disruption, cell pellets were washed in Tris-HCl (50 mM, pH 8) and transferred to 50 mL falcon tubes, centrifuged (15 min, 4 °C, 4,500 × *g*) and stored at -80 °C. On ice, the cells were re-suspended with 1:4 ratio cells to lysis buffer (50 mM Tris-HCl pH 8, 300 mM NaCl, 10 mM imidazole, 10% v/v glycerol, EDTA-free complete protease inhibitor pill, $MgCl_2$ (0.5 mM), DNase(0.1 mg/mL) and one spatula tip of lysozyme. The cells were disrupted at 1.7 kbar and centrifuged (40 min, 4 °C, 20,000 × *g*). For IMAC purification the supernatant was filtered (0.22 µm) and loaded on the column at 4 °C. Elution buffer was 50 mM Tris-HCl pH 8, 300 mM NaCl, 500 mM imidazole, 10% v/v glycerol. The collected fractions were concentrated with a 10 kDa Amicon filter, then passed through a PD-10 desalting column with buffer (50 mM Tris-HCl pH 8, 10% v/v glycerol), flash frozen in liquid nitrogen and stored at -80 °C (**Figure S10**).

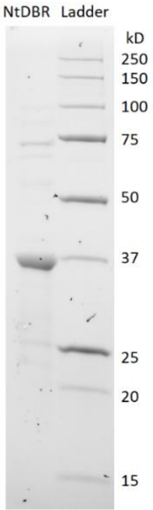

Figure S10. SDS-PAGE gel of purified *Nt*DBR.

The thermostable double mutant ***Bs*GDH E170K/Q252L** was recombinantly produced in *E. coli* BL21 Gold(DE3) competent cells with a pET-28a(+) vector harboring an *N*-terminal His-tag. A pre-culture (10 mL) of LB medium containing kanamycin (50 µg/mL) was inoculated with a single colony and incubated overnight at 37 °C, 180 rpm. TB medium (1.5 L in a 5 L shake-flask) containing kanamycin (50 µg/mL) and incubated at 37 °C, 180 rpm until an OD_{600} of 0.7 was reached. 500 µM of IPTG was added and incubated for 18 h at 25 °C and 180 rpm. Cells were harvested centrifugation (10,000 × *g*, 4 °C, 20 min), washed (50 mM Na Pi pH 8), centrifuged (4000 × *g*, 4 °C, 10 min) and stored at -20 °C (13 g wet cell pellet, 9 g/L). Cells were thawed, resuspended in lysis buffer (50 mM NaPi pH 8, 300 mM NaCl, 25 mM imidazole, EDTA-free complete protease inhibitor pill, $MgCl_2$ (0.5 mM), DNase (0.1 mg/mL) and lysozyme (~0.1 mg/mL)). The cells were disrupted at 1.35 kbar at 4 °C. The disrupted cells were centrifuged (10 min, 4 °C, 11,693 × *g*). The supernatant to be used for purification was collected and put into falcon tubes for heat bath (2 × 17.5 mL) at 60 °C for 1 h. The contents of the falcon tubes were combined and centrifuged (30 min, 4 °C, 11,693 × *g*). We noted that it was yellowish. The cell free extract was filtered (0.22 µm). To the supernatant was added 1.35 mL of buffer B (50 mM NaPi pH 8, 500 mM imidazole, 300 mM NaCl) such that the total amount of imidazole amounted to a concentration of buffer A (25 mM imidazole). Purification was carried out using a Bio-Rad NGC system, on a His-Trap FF crude (5 mL) column. After loading the sample, the column was washed with buffer A (10 CV). Elution started at 20% of buffer B (50 mM NaPi pH 8, 500 mM imidazole, 300 mM NaCl). The fractions were collected and washed and concentrated with a 15 mL 10 kDa Amicon filter with storage buffer (50 mM NaPi pH 8, 300 mM NaCl, 25 mM imidazole). Protein concentration was estimated with a BCA assay. The concentration was 391 µM (14.1 mg/mL, 32 mg/L culture). Aliquots were flash frozen in liquid nitrogen and stored at -80 °C (**Figure S11**).

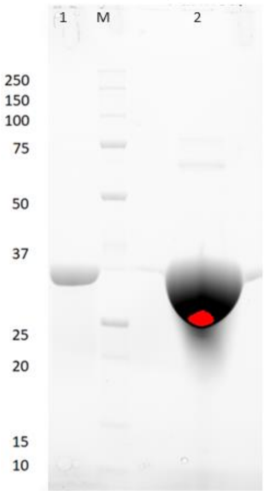

Figure S11. SDS-PAGE gel of *Bs*GDH E170K/Q252L double purification. Lanes 1 and 2 are the same double purification but at different concentrations to see purity, **M:** protein marker.

Enzyme sequences

GluER
MGSSHHHHHHSSGLVPRGSHMPTLFDPIDFGPIHAKNRIVMSPLTRGRADKEAVPTPIMAEYYAQRASAGLIITEATGISREGLG
WPFAPGIWSDAQVEAWKPIVAGVHAKGGKIVCQLWHMGRMVHSSVTGTQPVSSSATTAPGEVHTYEGKKPFEQARAIDAA
DISRILNDYENAARNAIRAGFDGVQIHAANGYLIDEFLRNGTNHRTDEYGGVPENRIRFLKEVTERVIAAIGADRTGVRLSPNGDT
QGCIDSAPETVFVPAAKLLQDLGVAWLELREPGPNGTFGKTDQPKLSPQIRKVFLRPLVLNQDYTFEAAQTALAEGKADAIAFG
RKFISNPDLPERFARGIALQPDDMKTWYSQGPEGYTDYPSATSGPN

ATGGGCAGCAGCCATCATCATCATCATCACAGCAGCGGCCTGGTGCCGCGCGGCAGCCATATGCCTACCCTGTTCGACCCG
ATCGACTTCGGTCCGATCCACGCTAAAAACCGTATCGTTATGTCTCCGCTGACCCGTGGCCGTGCGGATAAAGAAGCGGTG
CCGACCCCGATCATGGCTGAATACTACGCGCAGCGCGCGTCCGCGGGCCTGATCATCACTGAAGCAACCGGTATCTCTCGT
GAAGGCCTGGGTTGGCCGTTCGCGCCGGGTATCTGGTCTGACGCGCAGGTTGAAGCCTGGAAACCGATCGTTGCTGGCGT

TCACGCTAAAGGCGGTAAAATCGTTTGCCAGCTGTGGCACATGGGCCGTATGGTACACTCTTCTGTGACCGGCACCCAGCC
AGTTTCCTCCTCTGCTACCACTGCGCCGGGTGAAGTACACACTTATGAAGGCAAAAAACCGTTCGAACAGGCTCGTGCGAT
CGACGCGGCAGACATTTCTCGTATCCTGAACGATTATGAAAACGCTGCGCGTAACGCAATCCGCGCTGGTTTCGATGGCGT
TCAGATCCACGCAGCGAACGGTTACCTGATTGACGAGTTCCTGCGTAACGGCACCAACCACCGCACCGATGAATACGGTG
GCGTACCGGAAAACCGTATCCGTTTCCTGAAAGAAGTGACTGAACGTGTGATCGCAGCTATCGGTGCGGATCGTACCGGT
GTTCGTCTGTCTCCGAACGGTGACACCCAGGGTTGCATTGACTCTGCGCCGGAAACCGTGTTCGTTCCGGCGGCTAAACTG
CTGCAGGATCTGGGTGTTGCGTGGCTGGAACTGCGTGAACCGGGTCCGAACGGTACTTTCGGTAAAACCGATCAGCCGAA
ACTGTCTCCGCAGATCCGTAAAGTTTTCCTGCGTCCGCTGGTTCTGAACCAGGACTACACCTTCGAAGCAGCGCAGACCGC
TCTGGCGGAAGGTAAAGCTGACGCGATCGCTTTCGGTCGTAAATTCATCTCTAACCCGGACCTGCCGGAACGTTTCGCGCG
TGGTATCGCGCTGCAGCCGGACGACATGAAAACCTGGTACTCCCAGGGTCCGGAAGGTTACACCGACTACCCGTCCGCGA
CCAGCGGCCCGAACTAA

***Le*OPR1**
MENKVVEEKQVDKIPLMSPCKMGKFELCHRVVLAPLTRQRSYGYIPQPHAILHYSQRSTNGGLLIGEATVISETGIGYKDVPGIW
TKEQVEAWKPIVDAVHAKGGIFFCQIWHVGRVSNKDFQPNGEDPISCTDRGLTPQIRSNGIDIAHFTRPRRLTTDEIPQIVNEFR
VAARNAIEAGFDGVEIHGAHGYLIDQFMKDQVNDRSDKYGGSLENRCRFALEIVEAVANEIGSDRVGIRISPFAHYNEAGDTNP
TALGLYMVESLNKYDLAYCHVVEPRMKTAWEKIECTESLVPMRKAYKGTFIVAGGYDREDGNRALIEDRADLVAYGRLFISNPD
LPKRFELNAPLNKYNRDTFYTSDPIVGYTDYPFLETMTLEHHHHHH

ATGGAAAATAAAGTCGTTGAAGAGAAACAAGTAGACAAGATCCCTCTAATGAGCCCTTGTAAAATGGGAAAGTTTGAGTT
ATGTCATAGAGTTGTATTGGCACCATTAACAAGGCAAAGATCTTATGGTTATATTCCTCAACCACATGCTATACTTCATTACT
CACAAAGAAGTACAAATGGTGGCCTTCTAATAGGAGAGGCCACAGTAATATCTGAGACTGGCATAGGGTACAAAGATGTA
CCTGGTATATGGACAAAAGAGCAAGTGGAGGCTTGGAAACCAATTGTAGATGCAGTTCATGCTAAAGGAGGAATCTTCTT
TTGCCAAATTTGGCATGTTGGTAGAGTTTCCAACAAAGATTTTCAGCCCAATGGAGAGGATCCTATCTCCTGCACAGACAG
AGGACTAACACCTCAAATTCGTTCCAATGGCATAGATATTGCACACTTTACACGACCTAGACGGTTGACAACAGATGAAAT
TCCTCAAATTGTTAACGAATTTCGAGTTGCTGCTAGAAACGCAATTGAAGCTGGATTTGATGGGGTTGAGATCCACGGAGC
TCATGGCTATCTAATTGATCAGTTTATGAAAGATCAAGTTAACGATCGAAGTGATAAATATGGAGGGTCTTTAGAGAATCG
TTGTAGATTTGCACTTGAAATAGTGGAAGCAGTTGCAAATGAGATTGGATCTGACCGAGTTGGTATAAGGATATCCCCATT
TGCGCATTATAATGAAGCAGGGGACACGAACCCGACTGCTTTGGGACTTTACATGGTGGAATCGTTGAACAAGTATGATC
TCGCGTATTGCCATGTGGTTGAGCCTAGGATGAAAACAGCTTGGGAAAAAATTGAATGTACTGAAAGCCTTGTACCGATG
AGGAAGGCATATAAAGGTACTTTTATAGTAGCTGGTGGTTACGATAGAGAAGATGGAAACAGAGCTTTGATTGAAGATCG
AGCTGATCTTGTTGCGTATGGACGTTTATTCATATCTAATCCAGATTTACCAAAGCGATTTGAGCTAAATGCTCCTCTTAACA
AGTATAACAGAGACACATTTTATACTTCTGATCCAATTGTTGGCTATACTGATTATCCATTTCTAGAAACCATGACACTCGA
GCACCACCACCACCACCACTGA

NCR [2]
MPSLFDPIRFGAFTAKNRIWMAPLTRGRATRDHVPTEIMAEYYAQRASAGLIISEATGISQEGLGWPYAPGIWSDAQVEAWLPI
TQAVHDAGGLIFAQLWHMGRMVPSNVSGMQPVAPSASQAPGLGHTYDGKKPYDVARALRLDEIPRLLDDYEKAARHALKAG
FDGVQIHAANGYLIDEFIRDSTNHRHDEYGGAVENRIRLLKDVTERVIATIGKERTAVRLSPNGEIQGTVDSHPEQVFIPAAKMLS
DLDIAFLGMREGAVDGTFGKTDQPKLSPEIRKVFKPPLVLNQDYTFETAQAALDSGVADAISFGRPFIGNPDLPRRFFEKAPLTKD
VIETWYTQTPKGYTDYPLLGDLEHHHHHH

ATGCCGTCACTGTTCGATCCAATCCGCTTTGGGGCTTTCACTGCAAAAAATCGTATCTGGATGGCGCCGTTAACACGGGGT
CGGGCAACCCGTGACCATGTCCCAACAGAGATAATGGCTGAATACTATGCCCAACGCGCATCCGCGGGCTTGATCATCAG
CGAGGCGACCGGGATCAGCCAAGAGGGCCTGGGCTGGCCCTATGCACCAGGAATCTGGAGTGATGCGCAGGTCGAGGC
ATGGTTACCCATAACCCAAGCGGTACACGATGCCGGAGGTTTGATATTTGCACAACTGTGGCACATGGGGCGTATGGTGC
CTTCCAACGTTTCTGGAATGCAACCTGTCGCACCTAGCGCTTCACAAGCGCCCGGCTTGGGCCATACTTATGATGGCAAAA
AGCCATACGATGTAGCCAGAGCATTGAGACTTGACGAGATCCCACGGCTGCTGGACGACTATGAAAAGGCAGCTCGGCAC
GCACTGAAAGCTGGGTTCGATGGAGTTCAGATTCATGCTGCCAACGGATACCTGATTGACGAGTTCATCCGGGATTCAAC
AAATCATAGACACGACGAATACGGGGGGGCGGTTGAGAACAGAATACGGTTATTGAAGGATGTCACTGAGCGGGTTATC
GCAACCATCGGAAAGGAGCGCACAGCAGTGCGTTTAAGTCCGAATGGAGAGATACAAGGCACAGTAGACTCGCATCCAG
AACAGGTATTTATCCCGGCTGCAAAGATGTTATCTGATTTAGATATCGCGTTCCTTGGGATGCGCGAGGGTGCTGTAGACG
GGACATTTGGCAAAACAGACCAGCCCAAACTTTCGCCCGAGATCCGTAAAGTTTTCAAGCCACCCCTTGTTCTGAATCAAG
ATTACACTTTCGAGACTGCCCAGGCTGCGTTAGATTCGGGTGTAGCCGATGCAATCAGTTTTGGTCGTCCATTCATTGGGA
ATCCCGACTTACCGAGAAGATTCTTTGAAAAGGCACCGTTAACTAAGGACGTAATTGAGACTTGGTACACTCAGACTCCCA
AAGGTTACACCGACTATCCACTGTTAGGTGATCTCGAGCACCACCACCACCACCACTGA

NerA
MTSLFEPAQAGDIALANRIVMAPLTRNRSPGAIPNNLNATYYEQRATAGLIVTEGTPISQQGQGYADVPGLYKREAIEGWKKIT
DGVHSAGGKIVAQIWHVGRISHTSLQPHGGQPVAPSAITAKSKTYIINDDGTGAFAETSEPRALTIDDIGLILEDYRSGARAALEA
GFDGVEIHAANGYLIEQFLKSSTNQRTDDYGGSIENRARFLLEVVDAVAEEIGAGRTGIRLSPVTPANDIFEADPQPLYNYVVEQL
GKRNLAFIHVVEGATGGPRDFKQGDKPFDYASFKAAYRNAGGKGLWIANNGYDRQSAIEAVESGKVDAVAFGKAFIANPDLV
RRLKNDAPLNAPNQPTFYGGGAEGYTDYPALAQRPHHHHHH

ATGACCAGCCTGTTTGAACCGGCACAGGCAGGCGATATTGCACTGGCAAATCGCATTGTTATGGCACCGCTGACCCGTAAT
CGTTCTCCGGGTGCAATTCCGAATAATCTGAATGCCACCTATTATGAACAGCGTGCAACCGCAGGCCTGATTGTTACCGAA
GGCACCCCGATTAGCCAGCAGGGTCAGGGTTATGCAGATGTTCCGGGTCTGTATAAACGTGAAGCCATTGAAGGCTGGAA
AAAAATTACCGATGGTGTTCATAGCGCAGGCGGTAAAATTGTTGCACAGATTTGGCATGTTGGTCGTATTAGCCATACCAG
CCTGCAGCCGCATGGTGGTCAGCCTGTGGCTCCTAGCGCAATTACCGCAAAAAGCAAAACCTACATTATTAATGATGATGG
AACAGGTGCCTTTGCAGAAACCAGCGAACCGCGTGCACTGACCATTGATGATATTGGCCTGATTCTGGAAGATTATCGTAG
CGGTGCACGTGCAGCACTGGAAGCCGGTTTTGATGGTGTTGAAATCCATGCAGCCAATGGCTATCTGATTGAACAGTTTCT
GAAAAGCAGCACCAATCAGCGTACCGATGATTATGGTGGATCTATTGAGAATCGCGCTCGCTTTCTGCTGGAAGTTGTTGA
TGCAGTTGCCGAAGAAATTGGTGCCGGTCGTACCGGTATTCGTCTGTCTCCGGTTACACCGGCAAACGATATTTTTGAAGC
AGATCCGCAGCCGCTGTATAATTATGTTGTGGAACAGCTGGGTAAACGTAATCTGGCCTTTATTCATGTTGTTGAAGGTGC
AACCGGTGGTCCGCGTGATTTTAAACAGGGCGATAAACCGTTTGATTATGCCAGCTTTAAAGCAGCATATCGTAATGCCGG
TGGTAAAGGTCTGTGGATTGCCAATAATGGTTATGATCGTCAGAGCGCAATTGAAGCAGTTGAAAGCGGTAAAGTTGATG
CCGTTGCATTTGGCAAAGCCTTTATTGCAAATCCGGATCTGGTTCGTCGTCTGAAAAATGATGCACCGCTGAATGCACCGA
ATCAGCCGACCTTTTATGGTGGTGGTGCAGAAGGTTATACCGATTATCCGGCACTGGCACAGCGGCCGCATCATCACCATC
ATCACTAA

PETNR
MSAEKLFTPLKVGAVTAPNRVFMAPLTRLRSIEPGDIPTPLMGEYYRQRASAGLIISEATQISAQAKGYAGAPGLHSPEQIAAWK
KITAGVHAEDGRIAVQLWHTGRISHSSIQPGGQAPVSASALNANTRTSLRDENGNAIRVDTTTPRALELDEIPGIVNDFRQAVA
NAREAGFDLVELHSAHGYLLHQFLSPSSNQRTDQYGGSVENRARLVLEVVDAVCNEWSADRIGIRVSPIGTFQNVDNGPNEEA
DALYLIEELAKRGIAYLHMSETDLAGGKPYSEAFRQKVRERFHGVIIGAGAYTAEKAEDLIGKGLIDAVAFGRDYIANPDLVARLQK
KAELNPQRPESFYGGGAEGYTDYPSLHHHHHH

ATGTCCGCTGAAAAGCTGTTTACCCCACTGAAAGTGGGTGCCGTTACTGCCCCAAACCGCGTGTTTATGGCCCCACTTACCC
GTCTGCGCAGCATCGAGCCGGGCGATATCCCAACGCCATTGATGGGTGAGTATTACCGCCAGCGCGCCAGCGCGGGCCTG
ATTATCTCCGAAGCCACGCAGATTTCTGCTCAGGCAAAAGGCTACGCCGGTGCACCGGGTCTGCACAGCCCGGAACAGAT
CGCCGCGTGGAAAAAAATCACCGCAGGCGTGCATGCTGAAGATGGCCGTATTGCGGTTCAGCTGTGGCACACCGGTCGTA
TCTCACACAGCAGCATCCAGCCTGGCGGTCAGGCGCCGGTTTCTGCCTCTGCCCTGAACGCCAATACCCGCACTTCCCTGC
GCGATGAAAACGGTAATGCGATCCGCGTCGACACCACCACGCCACGCGCGCTGGAGCTGGACGAGATCCCGGGTATCGT
GAATGATTTCCGTCAGGCCGTCGCCAACGCCCGGGAAGCGGGCTTCGACCTGGTTGAGCTTCACTCTGCGCACGGTTACCT
GCTGCATCAGTTCCTGTCCCCGTCTTCCAACCAGCGTACCGACCAGTACGGCGGCAGCGTTGAAAACCGCGCGCGTCTGGT
GCTTGAAGTGGTGGATGCTGTCTGTAATGAGTGGAGCGCAGACCGCATTGGTATTCGTGTCTCCCCGATCGGTACTTTCCA
GAACGTCGACAACGGTCCGAACGAAGAAGCAGACGCGCTGTATCTGATTGAAGAGCTGGCGAAACGCGGTATCGCCTAT
CTGCACATGTCCGAGACGGACTTGGCAGGCGGCAAGCCTTACAGTGAAGCCTTCCGTCAGAAAGTGCGCGAGCGCTTCCA
CGGCGTGATTATCGGGGCGGGTGCGTATACGGCAGAGAAAGCCGAGGATTTGATCGGTAAAGGCCTGATCGACGCCGTG
GCCTTTGGCCGTGACTACATTGCTAACCCGGATCTGGTTGCCCGTTTGCAGAAAAAAGCCGAACTGAACCCGCAGCGTCCT
GAAAGCTTCTATGGCGGCGGCGCGGAAGGTTATACCGACTACCCTTCACTGCACCACCACCACCACCACTGA

XenB
MATIFDPIKLGDLELSNRIIMAPLTRCRADEGRVPNALMAEYYVQRASAGLILSEATSVTPMGVGYPDTPGIWSNDQVRGWTNI
TKAVHAAGGKIVLQLWHVGRISHPLYLNGEAPVAPSAIQPKGHVSLVRPLADYPTPRALETAEIAEIVEAYRTGAENAKAAGFDG
VEIHGANGYLLDQFLQSSTNQRTDNYGGSLENRARLLLEVTDAAIDVWGAGRVGVHLAPRADSHDMGDDNLAETFTYVAREL
GKRGIAFICSREKEGADSLGPQLKEAFGGAYIANERFTKDSANAWLAEGKADAVAFGVPFIANPDLPARLKADAPLNEPRPELFY
GKGPVGYIDYPTLRPHHHHHH

ATGGCCACCATCTTTGATCCGATTAAACTGGGTGATCTGGAACTGAGCAACCGTATTATTATGGCACCGCTGACCCGTTGT
CGTGCAGATGAAGGTCGTGTTCCGAATGCACTGATGGCCGAATATTATGTTCAGCGTGCAAGCGCAGGCCTGATTCTGAG
CGAAGCAACCAGCGTTACCCCGATGGGTGTTGGTTATCCGGATACACCGGGTATTTGGAGCAATGATCAGGTTCGTGGTT
GGACCAATATTACCAAAGCAGTTCATGCAGCCGGTGGTAAAATTGTTCTGCAGCTGTGGCATGTTGGTCGTATTAGCCATC
CGCTGTATCTGAATGGTGAAGCACCGGTTGCACCGAGCGCAATTCAGCCGAAAGGTCATGTTAGCCTGGTTCGTCCGCTG

GCAGATTATCCGACTCCTCGCGCTCTGGAAACCGCAGAAATTGCCGAAATTGTGGAAGCATATCGTACCGGTGCAGAAAA
TGCCAAAGCAGCCGGTTTTGATGGTGTTGAAATCCATGGTGCCAATGGTTATCTGCTGGATCAGTTTCTGCAGAGCAGCAC
CAATCAGCGTACCGATAATTATGGTGGCAGCCTGGAAAATCGTGCACGTCTGCTGCTGGAAGTTACCGATGCAGCAATTG
ATGTTTGGGGTGCCGGTCGTGTTGGTGTTCATCTGGCACCGCGTGCAGATAGCCATGATATGGGTGATGATAATCTGGCA
GAAACCTTTACCTATGTTGCACGTGAACTGGGTAAACGTGGCATTGCATTTATTTGCAGCCGTGAAAAAGAAGGTGCCGAT
AGCCTGGGTCCGCAGCTGAAAGAAGCATTTGGCGGAGCCTATATTGCCAATGAACGCTTTACCAAAGATAGCGCAAATGC
ATGGCTGGCAGAAGGTAAAGCAGACGCAGTTGCATTTGGTGTTCCGTTTATTGCAAATCCGGATCTGCCTGCACGTCTGAA
AGCCGATGCACCGCTGAATGAACCGCGTCCGGAACTGTTTTATGGTAAAGGTCCGGTGGGCTATATTGACTACCCTACATT
ACGGCCGCATCATCACCATCATCACTAA

OYE2
MGSSHHHHHHSSGLVPRGSHNMPFVKDFKPQALGDTNLFKPIKIGNNELLHRAVIPPLTRMRAQHPGNIPNRDWAVEYYAQR
AQRPGTLIITEGTFPSPQSGGYDNAPGIWSEEQIKEWTKIFKAIHENKSFAWVQLWVLGWAAFPDTLARDGLRYDSASDNVYM
NAEQEEKAKKANNPQHSITKDEIKQYVKEYVQAAKNSIAAGADGVEIHSANGYLLNQFLDPHSNNRTDEYGGSIENRARFTLEV
VDAVVDAIGPEKVGLRLSPYGVFNSMSGGAETGIVAQYAYVLGELERRAKAGKRLAFVHLVEPRVTNPFLTEGEGEYNGGSNKF
AYSIWKGPIIRAGNFALHPEVVREEVKDPRTLIGYGRFFISNPDLVDRLEKGLPLNKYDRDTFYKMSAEGYIDYPTYEEALKLGWD
KN

ATGGGCAGCAGCCATCATCATCATCATCACAGCAGCGGCCTGGTGCCGCGCGGCAGCCATAATATGCCATTTGTTAAGGA
CTTTAAGCCACAAGCTTTGGGTGACACCAACTTATTCAAACCAATCAAAATTGGTAACAATGAACTTCTACACCGTGCTGTC
ATTCCTCCATTGACTAGAATGAGAGCCCAACATCCAGGTAATATTCCAAACAGAGACTGGGCCGTTGAATACTACGCTCAA
CGTGCTCAAAGACCAGGAACCTTGATTATCACTGAAGGTACCTTTCCCTCTCCACAATCTGGGGGTTACGACAATGCTCCA
GGTATCTGGTCCGAAGAACAAATTAAAGAATGGACCAAGATTTTCAAGGCTATTCATGAGAATAAATCGTTCGCATGGGTC
CAATTATGGGTTCTAGGTTGGGCTGCTTTCCCAGACACCCTTGCTAGGGATGGTTTGCGTTACGACTCCGCTTCTGACAACG
TGTATATGAATGCAGAACAAGAAGAAAAGGCTAAGAAGGCTAACAACCCACAACACAGTATAACAAAGGATGAAATTAA
GCAATACGTCAAAGAATACGTCCAAGCTGCCAAAAACTCCATTGCTGCTGGTGCCGATGGTGTTGAAATCCACAGCGCTAA
CGGTTACTTGTTGAACCAGTTCTTGGACCCACACTCCAATAACAGAACCGATGAGTATGGTGGTTCCATCGAAAACAGAGC
CCGTTTCACCTTGGAAGTGGTTGATGCAGTTGTCGATGCTATTGGCCCTGAAAAAGTCGGTTTGAGATTGTCTCCCTATGG
TGTCTTCAACAGTATGTCTGGTGGTGCTGAAACCGGTATTGTTGCTCAATATGCTTATGTCTTAGGTGAACTAGAAAGAAG
AGCTAAAGCTGGCAAGCGTTTGGCTTTCGTCCATCTAGTTGAACCTCGTGTCACCAACCCATTTTTAACTGAAGGTGAAGG
TGAATACAATGGAGGTAGCAACAAATTTGCTTATTCTATCTGGAAGGGCCCAATTATTAGAGCTGGTAACTTTGCTCTGCA
CCCAGAAGTTGTCAGAGAAGAGGTGAAGGACCCTAGAACATTGATCGGTTACGGTAGATTTTTTATCTCTAATCCAGATTT
GGTTGATCGTTTGGAAAAAGGGTTACCATTAAACAAATATGACAGAGACACTTTCTACAAAATGTCAGCTGAGGGATACAT
TGACTACCCTACGTACGAAGAAGCTCTAAAACTCGGTTGGGACAAAAATTAA

OYE3
MGSSHHHHHHSSGLVPRGSHNMPFVKGFEPISLRDTNLFEPIKIGNTQLAHRAVMPPLTRMRATHPGNIPNKEWAAVYYGQR
AQRPGTMIITEGTFISPQAGGYDNAPGIWSDEQVAEWKNIFLAIHDCQSFAWVQLWSLGWASFPDVLARDGLRYDCASDRVY
MNATLQEKAKDANNLEHSLTKDDIKQYIKDYIHAAKNSIAAGADGVEIHSANGYLLNQFLDPHSNKRTDEYGGTIENRARFTLEV
VDALIETIGPERVGLRLSPYGTFNSMSGGAEPGIIAQYSYVLGELEKRAKAGKRLAFVHLVEPRVTDPSLVEGEGEYSEGTNDFAYS
IWKGPIIRAGNYALHPEVVREQVKDPRTLIGYGRFFISNPDLVYRLEEGLPLNKYDRSTFYTMSAEGYTDYPTYEEAVDLGWNKN

ATGGGCAGCAGCCATCATCATCATCATCACAGCAGCGGCCTGGTGCCGCGCGGCAGCCATAATATGCCATTTGTAAAAGG
TTTTGAGCCGATCTCCCTAAGAGACACAAACCTTTTTGAACCAATTAAGATTGGTAACACTCAGCTTGCACATCGTGCGGTT
ATGCCCCCATTGACCAGAATGAGGGCCACTCACCCCGGAAATATTCCAAATAAGGAGTGGGCTGCTGTGTATTATGGTCA
GCGTGCTCAAAGACCTGGTACCATGATCATCACGGAAGGTACGTTTATTTCCCCTCAAGCCGGCGGCTATGACAACGCCCC
TGGGATTTGGTCTGATGAGCAGGTCGCTGAGTGGAAGAATATCTTTTTAGCCATCCATGATTGTCAGTCGTTCGCGTGGGT
ACAACTTTGGTCTTTAGGCTGGGCATCCTTCCCAGACGTATTGGCAAGAGACGGGTTACGCTATGACTGTGCATCTGACAG
AGTGTATATGAATGCTACGTTACAAGAAAAGGCCAAAGATGCGAATAATCTCGAACATAGTTTGACTAAAGACGACATTA
AACAGTATATCAAGGATTACATCCATGCGGCTAAGAATTCTATCGCGGCTGGCGCCGATGGTGTAGAAATTCATAGCGCC
AATGGGTACTTGTTGAATCAGTTCTTGGATCCACATTCTAATAAGAGGACCGACGAATACGGCGGAACGATCGAAAACAG
GGCCCGCTTTACACTGGAGGTTGTCGATGCTCTTATCGAAACTATCGGTCCTGAACGGGTGGGTTTGAGGTTGTCGCCGTA
CGGCACTTTTAACAGTATGTCTGGGGGTGCTGAACCAGGTATTATCGCTCAATATTCGTATGTTTTGGGTGAATTAGAGAA
GAGGGCAAAGGCTGGTAAGCGTTTGGCCTTTGTGCACCTCGTTGAACCACGTGTCACGGACCCATCGTTGGTGGAGGGCG
AAGGAGAATATTCCGAGGGTACTAACGATTTTGCCTACTCTATATGGAAGGGTCCAATCATCAGAGCTGGTAATTACGCTC
TTCATCCAGAAGTGGTTAGAGAACAAGTAAAGGATCCCAGAACCTTGATAGGCTATGGTAGATTCTTCATCTCTAACCCAG

ATTTAGTCTACCGTTTAGAGGAGGGCCTGCCATTGAACAAGTATGACAGAAGTACCTTCTACACCATGTCCGCGGAAGGTT
ATACCGACTACCCAACATATGAAGAGGCAGTAGATTTAGGTTGGAACAAGAACTGA

OYE3 Y197F
MGSSHHHHHHSSGLVPRGSHMPFVKGFEPISLRDTNLFEPIKIGNTQLAHRAVMPPLTRMRATHPGNIPNKEWAAVYYGQRA
QRPGTMIITEGTFISPQAGGYDNAPGIWSDEQVAEWKNIFLAIHDCQSFAWVQLWSLGWASFPDVLARDGLRYDCASDRVY
MNATLQEKAKDANNLEHSLTKDDIKQYIKDYIHAAKNSIAAGADGVEIHSANGFLLNQFLDPHSNKRTDEYGGTIENRARFTLEV
VDALIETIGPERVGLRLSPYGTFNSMSGGAEPGIIAQYSYVLGELEKRAKAGKRLAFVHLVEPRVTDPSLVEGEGEYSEGTNDFAYS
IWKGPIIRAGNYALHPEVVREQVKDPRTLIGYGRFFISNPDLVYRLEEGLPLNKYDRSTFYTMSAEGYTDYPTYEEAVDLGWNKN
LEHHHHHH

ATGGGCAGCAGCCATCATCATCATCATCACAGCAGCGGCCTGGTGCCGCGCGGCAGCCATATGCCGTTCGTTAAAGGTTTC
GAACCGATCTCCCTGCGTGACACCAACCTGTTCGAACCGATCAAAATCGGTAACACCCAGCTGGCTCACCGTGCTGTTATG
CCGCCGCTGACCCGTATGCGTGCGACCCATCCGGGCAACATCCCGAACAAAGAATGGGCAGCTGTGTACTACGGCCAGCG
TGCGCAGCGTCCAGGTACCATGATCATCACCGAAGGTACTTTCATCTCTCCGCAGGCGGGTGGTTACGATAACGCGCCGG
GTATCTGGTCTGATGAACAGGTTGCTGAATGGAAAAACATCTTCCTGGCTATCCACGATTGTCAGTCTTTCGCATGGGTTCA
GCTGTGGTCCCTGGGCTGGGCGTCTTTCCCGGATGTTCTGGCCCGTGACGGCCTGCGCTATGATTGTGCTAGCGATCGTGT
TTACATGAACGCGACCCTGCAGGAAAAAGCTAAAGACGCGAACAACCTGGAACACTCTCTGACCAAAGACGACATCAAAC
AGTACATCAAAGACTACATCCACGCTGCTAAAAACTCCATCGCGGCTGGTGCCGATGGTGTTGAAATCCACTCCGCGAACG
GCTTCCTGCTGAACCAGTTCCTGGACCCGCACTCTAACAAACGCACCGACGAATACGGTGGCACCATTGAAAACCGTGCAC
GTTTCACCCTGGAAGTTGTGGACGCGCTGATCGAAACCATCGGCCCGGAACGTGTTGGTCTGCGTCTGTCTCCGTACGGTA
CCTTCAACAGCATGTCCGGCGGCGCGGAACCGGGTATCATCGCGCAGTACTCTTACGTTCTGGGTGAACTGGAAAAACGT
GCGAAAGCGGGTAAACGTCTGGCGTTCGTTCACCTGGTTGAACCGCGTGTTACTGACCCATCTCTGGTTGAAGGTGAAGG
CGAATACTCTGAGGGCACCAACGACTTCGCTTACTCTATCTGGAAAGGTCCGATTATCCGTGCGGGTAACTACGCTCTGCA
CCCGGAAGTGGTTCGTGAACAGGTTAAAGACCCGCGTACCCTGATCGGTTACGGTCGTTTCTTCATCTCTAACCCGGACCT
GGTTTACCGTCTGGAAGAAGGCCTGCCGCTGAACAAATACGACCGTTCCACCTTCTACACCATGTCTGCAGAAGGTTACAC
CGACTACCCGACCTATGAAGAAGCTGTTGACCTGGGTTGGAACAAAAACCTCGAGCACCACCACCACCACCACTGA

EBP1
MGSSHHHHHHSSGLVPRGSHMTIESTNSFVVPSDTKLIDVTPLGSTKLFQPIKVGNNVLPQRIAYVPTTRFRASKDHIPSDLQLN
YYNARSQYPGTLIITEATFASERGGIDLHVPGIYNDAQAKSWKKINEAIHGNGSFSSVQLWYLGRVANAKDLKDSGLPLIAPSAVY
WDENSEKLAKEAGNELRALTEEEIDHIVEVEYPNAAKHALEAGFDYVEIHGAHGYLLDQFLNLASNKRTDKYGCGSIENRARLLLR
VVDKLIEVVGANRLALRLSPWASFQGMEIEGEEIHSYILQQLQQRADNGQQLAYISLVEPRVTGIYDVSLKDQQGRSNEFAYKIW
KGNFIRAGNYTYDAPEFKTLINDLKNDRSIIGFSRFFTSNPDLVEKLKLGKPLNYYNREEFYKYYNYGYNSYDESEKQVIGKPLALEH
HHHHH

ATGGGCAGCAGCCATCATCATCATCATCACAGCAGCGGCCTGGTGCCGCGCGGCAGCCATATGACCATCGAATCTACTAAC
TCTTTCGTTGTGCCGTCTGACACCAAACTGATCGACGTTACCCCGCTGGGTTCTACCAAACTGTTCCAGCCGATCAAAGTTG
GTAACAACGTTCTGCCGCAGCGTATCGCATACGTTCCGACCACCCGTTTCCGTGCGTCTAAAGACCACATTCCGTCTGACCT
GCAGCTGAACTACTACAACGCACGTTCTCAGTACCCAGGTACCCTGATCATCACCGAAGCGACTTTCGCTTCTGAACGCGG
CGGTATCGATCTGCACGTTCCGGGCATCTACAACGATGCGCAGGCGAAATCTTGGAAAAAGATCAACGAAGCTATCCACG
GTAACGGTTCTTTCTCTTCTGTTCAGCTGTGGTACCTGGGTCGTGTAGCTAACGCAAAAGACCTGAAAGACTCTGGTCTGCC
GCTGATCGCTCCGTCTGCTGTTTACTGGGATGAAAACTCTGAAAAACTGGCGAAAGAAGCTGGCAACGAACTGCGTGCTC
TGACCGAAGAAGAAATCGACCACATTGTGGAAGTTGAATACCCGAACGCTGCGAAACACGCGCTGGAAGCAGGTTTCGAT
TACGTTGAAATCCACGGTGCGCATGGTTACCTGCTGGATCAGTTCCTGAACCTGGCTTCCAACAAACGTACCGATAAATAT
GGTTGCGGCTCTATTGAAAACCGTGCGCGTCTGCTGCTGCGTGTTGTTGATAAACTGATCGAAGTTGTGGGTGCGAACCG
TCTGGCGCTGCGTCTGTCTCCGTGGGCATCCTTCCAGGGCATGGAAATCGAAGGTGAAGAAATCCACTCTTACATCCTGCA
GCAGCTGCAGCAGCGTGCTGATAACGGCCAGCAGCTGGCTTACATCTCCCTGGTTGAACCGCGTGTTACCGGTATCTACGA
TGTTTCCCTGAAAGACCAGCAGGGTCGTTCTAACGAATTCGCATACAAAATTTGGAAAGGTAACTTCATCCGTGCTGGTAA
CTACACCTACGACGCGCCGGAATTCAAAACCCTGATCAACGACCTGAAAAACGACCGTTCTATCATCGGTTTCTCTCGTTTC
TTCACCTCTAACCCGGACCTGGTTGAAAAACTGAAACTGGGTAAACCGCTGAACTACTATAACCGTGAAGAATTCTACAAA
TACTACAACTACGGTTACAACAGCTACGATGAATCTGAAAAACAGGTTATCGGTAAACCGCTGGCTCTCGAGCACCACCAC
CACCACCACTGA

***Gk*OYE**
MGSSHHHHHHSSGLVPRGSHMNTMLFSPYTIRGLTLKNRIVMSPMCMYSCDTKDGAVRTWHKIHYPARAVGQVGLIIVEAT
GVTPQGRISERDLGIWSDDHIAGLRELVGLVKEHGAAIGIQLAHAGRKSQVPGEIIAPSAVPFDDSSPTPKEMTKADIEETVQAF

QNGARRAKEAGFDVIEIHAAHGYLINEFLSPLSNRRQDEYGGSPENRYRFLGEVIDAVREVWDGPLFVRISASDYHPDGLTAKDY
VPYAKRMKEQGVDLVDVSSGAIVPARMNVYPGYQVPFAELIRREADIPTGAVGLITSGWQAEEILQNGRADLVFLGRELLRNPY
WPYAAARELGAKISAPVQYERGWRF

ATGGGCAGCAGCCATCATCATCATCATCACAGCAGCGGCCTGGTGCCGCGCGGCAGCCATATGAACACCATGCTGTTCTCT
CCGTACACCATCCGTGGTCTGACCCTGAAAAACCGTATCGTTATGTCTCCGATGTGCATGTACTCTTGCGATACCAAAGACG
GTGCTGTTCGTACCTGGCACAAAATCCACTACCCGGCTCGTGCGGTTGGTCAGGTTGGTCTGATTATCGTTGAAGCGACCG
GTGTTACCCCGCAGGGCCGTATCTCTGAACGCGACCTGGGTATCTGGTCTGATGACCACATCGCGGGTCTGCGTGAACTG
GTTGGTCTGGTTAAAGAACACGGTGCAGCGATCGGTATCCAGCTGGCGCACGCGGGTCGTAAATCTCAGGTTCCGGGCGA
AATCATCGCGCCGTCTGCAGTTCCGTTCGACGATTCCTCCCCGACCCCGAAAGAAATGACCAAAGCTGACATCGAAGAAAC
TGTTCAGGCATTCCAGAACGGTGCGCGTCGTGCTAAAGAAGCGGGTTTCGACGTTATCGAGATCCACGCGGCGCACGGTT
ACCTGATCAACGAGTTCCTGTCTCCGCTGTCTAACCGCCGTCAGGATGAATACGGTGGTTCTCCGGAAAACCGTTACCGTTT
CCTGGGTGAAGTAATCGATGCTGTTCGCGAAGTTTGGGACGGCCCGCTGTTCGTTCGTATCAGCGCGTCCGATTACCACCC
GGACGGTCTGACCGCGAAAGACTACGTGCCGTATGCTAAACGTATGAAAGAACAGGGTGTTGACCTGGTTGATGTGTCCT
CTGGTGCTATCGTTCCGGCACGTATGAACGTTTATCCGGGTTACCAGGTTCCGTTCGCGGAACTGATCCGTCGTGAAGCTG
ACATCCCGACCGGTGCGGTTGGTCTGATCACCTCTGGTTGGCAGGCGGAAGAAATCCTGCAGAACGGTCGTGCGGATCTG
GTTTTCCTGGGTCGTGAACTGCTGCGTAACCCGTACTGGCCGTACGCGGCAGCGCGTGAACTGGGTGCTAAAATCTCTGCT
CCGGTTCAGTACGAACGTGGTTGGCGTTTCTAA

***T*OYE**
MSILHMPLKIKDITIKNRIMMSPMCMYSASTDGMPNDWHIVHYATRAIGGVGLIMQEATAVESRGRITDHDLGIWNDEQVKE
LKKIVDICKANGAVMGIQLAHAGRKCNISYEDVVGPSPIKAGDRYKLPRELSVEEIKSIVKAFGEAAKRANLAGYDVVEIHAAHGY
LIHEFLSPLSNKRKDEYGNSIENRARFLIEVIDEVRKNWPENKPIFVRVSADDYMEGGINIDMMVEYINMIKDKVDLIDVSSGGLL
NVDINLYPGYQVKYAETIKKRCNIKTSAVGLITTQELAEEILSNERADLVALGRELLRNPYWVLHTYTSKEDWPKQYERAFKKLEH
HHHHH

ATGAGCATTCTGCACATGCCGCTGAAAATCAAAGACATCACCATCAAAAACCGCATCATGATGTCCCCGATGTGCATGTAT
TCTGCCTCTACCGATGGTATGCCAAACGACTGGCATATCGTTCACTATGCGACTCGTGCGATCGGTGGTGTAGGTCTGATT
ATGCAGGAGGCTACTGCTGTTGAATCTCGTGGTCGTATTACGGATCACGATCTGGGTATCTGGAACGACGAACAGGTCAA
AGAGCTGAAAAAAATCGTTGACATCTGCAAAGCCAACGGTGCAGTTATGGGTATTCAGCTGGCGCATGCAGGTCGCAAAT
GTAACATCTCCTACGAAGATGTCGTTGGTCCGTCTCCGATTAAAGCAGGTGACCGTTACAAACTGCCGCGTGAACTGTCCG
TTGAGGAAATCAAAAGCATCGTGAAAGCGTTTGGTGAGGCTGCGAAACGTGCTAACCTGGCTGGTTACGACGTTGTAGAA
ATCCATGCGGCACATGGTTACCTGATCCATGAATTCCTGTCTCCGCTGTCCAACAAACGTAAAGACGAGTACGGCAACTCC
ATTGAAAACCGTGCTCGTTTCCTGATCGAAGTGATCGATGAAGTGCGCAAAAACTGGCCGGAAAACAAACCAATCTTCGTT
CGTGTGTCTGCGGATGATTATATGGAGGGCGGCATTAACATCGACATGATGGTGGAATACATCAACATGATCAAAGACAA
AGTAGATCTGATCGATGTCAGCTCTGGCGGTCTGCTGAACGTAGATATCAACCTGTACCCGGGTTACCAGGTGAAATACGC
CGAAACGATCAAAAAACGCTGCAACATCAAAACCTCTGCAGTAGGTCTGATCACTACCCAGGAACTGGCAGAAGAAATCC
TCTCTAACGAACGTGCTGACCTGGTTGCGCTGGGTCGTGAACTGCTGCGTAACCCGTATTGGGTTCTGCACACCTATACCA
GCAAAGAAGACTGGCCGAAACAGTACGAACGTGCCTTCAAAAAACTCGAGCACCACCACCACCACCACT

***Ts*OYE**
MGSSHHHHHHSSGLVPRGSHMALLFTPLELGGLRLKNRLAMSPMCQYSATLEGEVTDWHLLHYPTRALGGVGLILVEATAVEP
LGRISPYDLGIWSEDHLPGLKELARRIREAGAVPGIQLAHAGRKAGTARPWEGGKPLGWRVVGPSPIPFDEGYPVPEPLDEAG
MERILQAFVEGARRALRAGFQVIELHMAHGYLLSSFLSPLSNQRTDAYGGSLENRMRFPLQVAQAVREVVPRELPLFVRVSATD
WGEGGWSLEDTLAFARRLKELGVDLLDCSSGGVVLRVRIPLAPGFQVPFADAVRKRVGLRTGAVGLITTPEQAETLLQAGSADL
VLLGRVLLRDPYFPLRAAKALGVAPEVPPQYQRGF

ATGGGCAGCAGCCATCATCATCATCATCACAGCAGCGGCCTGGTGCCGCGCGGCAGCCATATGGCCTTGCTCTTCACCCCC
CTGGAACTCGGCGGCCTCCGGCTGAAAAACCGCCTGGCCATGTCCCCCATGTGCCAGTACTCCGCCACCTTGGAGGGAGA
GGTAACCGACTGGCACCTCCTCCACTACCCCACGCGGGCCCTTGGGGGCGTGGGGCTCATTCTGGTGGAGGCCACCGCCG
TGGAACCTTTGGGCCGTATCAGCCCCTATGACCTGGGCATCTGGTCGGAGGATCACCTTCCGGGCCTGAAGGAGCTCGCC
CGGAGGATCCGGGAAGCTGGAGCGGTGCCGGGGATCCAGCTGGCCCACGCCGGGCGCAAGGCGGGGACCGCCAGGCCC
TGGGAAGGGGGAAAGCCCCTGGGCTGGCGGGTGGTGGGGCCAAGCCCCATTCCCTTTGACGAGGGCTACCCGGTACCCG
AACCCCTGGACGAAGCAGGGATGGAGCGCATCCTCCAGGCCTTCGTGGAAGGAGCCAGACGTGCCCTTAGGGCAGGCTT
TCAGGTGATCGAGCTCCACATGGCCCATGGCTACCTCCTTTCCTCCTTCCTCTCCCCCCTTTCCAACCAGCGCACCGACGCCT
ACGGGGGAAGCCTGGAAAACCGCATGCGCTTTCCCCTCCAGGTGGCCCAGGCAGTGCGGGAGGTGGTGCCCAGGGAGCT
TCCCCTTTTCGTGCGGGTCTCCGCCACGGACTGGGGGGAAGGAGGATGGAGCCTCGAGGACACCCTGGCCTTCGCCCGGA

GGCTTAAGGAGCTGGGGGTGGACCTTTTGGACTGCTCCTCGGGCGGGGTGGTGCTCAGGGTGCGGATTCCCCTGGCCCC
GGGCTTTCAGGTGCCCTTCGCCGACGCCGTGCGCAAGAGGGTGGGCCTGCGAACGGGAGCCGTGGGCCTCATCACCACC
CCCGAGCAGGCGGAAACCCTCCTGCAGGCGGGAAGCGCCGATCTGGTGCTTCTGGGCCGGGTTCTCCTCAGGGACCCCTA
CTTCCCCTTACGGGCTGCCAAGGCCTTGGGCGTGGCCCCGGAGGTACCCCCCCAGTACCAAAGGGGGTTTTAG

XenA
MGSSHHHHHHSSGLVPRGSHMSALFEPYTLKDVTLRNRIAIPPMCQYMAEDGLINDWHQVHYASMARGGAGLLVVEATAVA
PEGRITPGCAGIWSDAHAQAFVPVVQAIKAAGSVPGIQIAHAGRKASANRPWEGDDHIGADDARGWETIAPSAIAFGAHLPN
VPRAMTLDDIARVKQDFVDAARRARDAGFEWIELHFAHGYLGQSFFSEHSNKRTDAYGGSFDNRSRFLLETLAAVREVWPENL
PLTARFGVLEYDGRDEQTLEESIELARRFKAGGLDLLSVSVGFTIPETNIPWGPAFMGPIAERVRREAKLPVTSAWGFGTPQLAE
AALQANQLDLVSVGRAHLADPHWAYFAAKELGVEKASWTLPAPYAHWLERYR

ATGGGCAGCAGCCATCATCATCATCATCACAGCAGCGGCCTGGTGCCGCGCGGCAGCCATATGTCCGCACTGTTCGAACC
CTACACCCTCAAAGACGTCACCCTGCGCAACCGCATCGCCATTCCGCCCATGTGCCAGTACATGGCCGAGGATGGCCTGAT
CAACGACTGGCACCAGGTGCATTACGCCAGCATGGCCCGTGGCGGTGCCGGCCTGCTGGTGGTCGAAGCCACGGCGGTG
GCACCGGAAGGGCGCATCACCCCTGGTTGCGCCGGAATCTGGAGCGATGCCCACGCCCAGGCTTTCGTGCCGGTGGTGCA
GGCCATCAAGGCGGCCGGTTCGGTGCCGGGCATCCAGATTGCGCACGCCGGCCGCAAGGCCAGCGCCAACCGCCCGTGG
GAGGGTGACGACCACATTGGTGCTGATGACGCGCGTGGCTGGGAGACCATCGCCCCGTCTGCCATTGCCTTTGGCGCGCA
CTTGCCAAACGTGCCGCGCGCAATGACCCTGGACGACATCGCCCGGGTCAAGCAGGACTTCGTCGATGCCGCCCGCCGTG
CGCGTGATGCCGGCTTCGAGTGGATCGAACTGCACTTCGCCCATGGCTACCTGGGCCAGAGCTTTTTCTCCGAGCACTCCA
ACAAGCGTACCGACGCGTACGGTGGCAGCTTCGACAACCGCAGTCGTTTCCTGCTGGAAACCCTGGCAGCGGTACGTGAG
GTGTGGCCAGAAAACCTGCCGCTGACCGCACGCTTTGGTGTGCTGGAATACGACGGCCGCGACGAGCAGACCCTGGAAG
AGTCGATCGAGCTGGCGCGCCGCTTCAAGGCGGGTGGCCTGGACCTGCTGAGCGTGAGTGTCGGTTTCACCATTCCCGAG
ACCAACATCCCGTGGGGCCCGGCGTTCATGGGGCCGATCGCCGAACGCGTGCGCCGTGAGGCGAAGCTGCCGGTGACCT
CGGCGTGGGGCTTTGGTACGCCGCAACTGGCTGAAGCCGCGTTGCAGGCCAACCAGTTGGACCTGGTGTCGGTAGGGCG
CGCGCATTTGGCTGATCCGCACTGGGCGTACTTTGCGGCCAAGGAGCTGGGCGTGGAAAAAGCCTCCTGGACCTTGCCGG
CGCCGTATGCGCACTGGCTTGAGCGCTATCGCTAA

YqjM
MGSSHHHHHHSSGLVPRGSHMARKLFTPITIKDMTLKNRIVMSPMCMYSSHEKDGKLTPFHMAHYISRAIGQVGLIIVEASAV
NPQGRITDQDLGIWSDEHIEGFAKLTEQVKEQGSKIGIQLAHAGRKAELEGDIFAPSAIAFDEQSATPVEMSAEKVKETVQEFKQ
AAARAKEAGFDVIEIHAAHGYLIHEFLSPLSNHRTDEYGGSPENRYRFLREIIDEVKQVWDGPLFVRVSASDYTDKGLDIADHIGF
AKWMKEQGVDLIDCSSGALVHADINVFPGYQVSFAEKIREQADMATGAVGMITDGSMAEEILQNGRADLIFIGRELLRDPFFA
RTAAKQLNTEIPAPVQYERGW

ATGGGCAGCAGCCATCATCATCATCATCACAGCAGCGGCCTGGTGCCGCGCGGCAGCCATATGGCCAGAAAATTATTTAC
ACCTATTACAATTAAAGATATGACGTTAAAAAACCGCATTGTCATGTCGCCAATGTGCATGTATTCTTCTCATGAAAAGGAC
GGAAAATTAACACCGTTCCACATGGCACATTACATATCGCGCGCAATCGGCCAGGTCGGACTGATTATTGTAGAGGCGTC
AGCGGTTAACCCTCAAGGACGAATCACTGACCAAGACTTAGGCATTTGGAGCGACGAGCATATTGAAGGCTTTGCAAAAC
TGACTGAGCAGGTCAAAGAACAAGGTTCAAAAATCGGCATTCAGCTTGCCCATGCCGGACGTAAAGCTGAGCTTGAAGGA
GATATCTTCGCTCCATCGGCGATTGCGTTTGACGAACAATCAGCAACACCTGTAGAAATGTCAGCAGAAAAAGTAAAAGA
AACGGTCCAGGAGTTCAAGCAAGCGGCTGCCCGCGCAAAAGAAGCCGGCTTTGATGTGATTGAAATTCATGCGGCGCAC
GGATATTTAATTCATGAATTTTTGTCTCCGCTTTCCAACCATCGAACAGATGAATATGGCGGCTCACCTGAAAACCGCTATC
GTTTCTTGAGAGAGATCATTGATGAAGTCAAACAAGTATGGGACGGTCCTTTATTTGTCCGTGTATCTGCTTCTGACTACAC
TGATAAAGGCTTAGACATTGCCGATCACATCGGTTTTGCAAAATGGATGAAGGAGCAGGGTGTTGACTTAATTGACTGCA
GCTCAGGCGCCCTTGTTCACGCAGACATTAACGTATTCCCTGGCTATCAGGTCAGCTTCGCTGAGAAAATCCGTGAACAGG
CGGACATGGCTACTGGTGCCGTCGGCATGATTACAGACGGTTCAATGGCTGAAGAAATTCTGCAAAACGGACGTGCCGAC
CTCATCTTTATCGGCAGAGAGCTTTTGCGGGATCCATTTTTTGCAAGAACTGCTGCGAAACAGCTCAATACAGAGATTCCG
GCCCCTGTTCAATACGAAAGAGGCTGGTAA

***Nt*DBR**
MGSSHHHHHHSSGLVPRGSHMAEEVSNKQVILKNYVTGYPKESDMEIKNVTIKLKVPEGSNDVVVKNLYLSCDPYMRSRMRKI
EGSYVESFAPGSPITGYGVAKVLESGDPKFQKGDLVWGMTGWEEYSIITPTQTLFKIHDKDVPLSYYTGILGMPGMTAYAGFHE
VCSPKKGETVFVSAASGAVGQLVGQFAKMLGCYVVGSAGSKEKVDLLKSKFGFDEAFNYKEEQDLSAALKRYFPDGIDIYFENV
GGKMLDAVLVNMKLYGRIAVCGMISQYNLEQTEGVHNLFCLITKRIRMEGFLVFDYYHLYPKYLEMVIPQIKAGKVVYVEDVAH
GLESAPTALVGLFSGRNIGKQVVMVSRE

ATGGGCAGCAGCCATCATCATCATCATCACAGCAGCGGCCTGGTGCCGCGCGGCAGCCATATGGCTGAAGAAGTTTCTAA
CAAACAGGTTATCCTGAAAAACTACGTTACCGGTTACCCGAAAGAATCTGACATGGAAATCAAAAACGTTACCATCAAACT
GAAAGTTCCGGAAGGCTCTAACGACGTTGTTGTTAAAAACCTGTACCTGTCTTGCGACCCGTACATGCGTTCTCGTATGCG
TAAAATCGAAGGTTCCTACGTTGAATCTTTCGCGCCGGGCTCCCCGATCACCGGTTACGGCGTTGCGAAAGTGCTGGAATC
TGGTGACCCGAAATTCCAGAAAGGTGACCTGGTTTGGGGTATGACCGGTTGGGAAGAATACTCTATCATCACCCCGACCC
AGACCCTGTTCAAAATCCACGACAAAGACGTTCCGCTGTCCTACTACACCGGTATCCTGGGTATGCCGGGTATGACCGCGT
ACGCGGGTTTCCACGAAGTTTGCTCTCCGAAAAAAGGTGAAACCGTTTTCGTTAGCGCTGCGTCTGGTGCGGTTGGTCAGC
TGGTTGGTCAGTTCGCGAAAATGCTGGGTTGCTACGTTGTTGGTTCTGCGGGTTCCAAAGAAAAAGTTGACCTGCTGAAAT
CTAAATTCGGTTTCGACGAAGCGTTCAACTACAAAGAAGAACAGGACCTGTCTGCGGCACTGAAACGTTACTTCCCGGACG
GCATCGACATCTACTTCGAAAACGTTGGTGGCAAAATGCTGGACGCTGTGCTGGTTAACATGAAACTGTACGGTCGTATCG
CGGTTTGCGGCATGATCTCCCAGTACAACCTGGAACAGACCGAAGGCGTTCACAACCTGTTCTGCCTGATCACCAAACGTA
TCCGTATGGAAGGCTTCCTGGTTTTCGACTACTACCACCTGTACCCGAAATACCTGGAAATGGTTATCCCGCAGATCAAAG
CGGGTAAAGTTGTTTACGTTGAAGATGTTGCGCACGGTCTGGAATCTGCTCCGACCGCTCTGGTTGGTCTGTTCTCTGGTC
GTAACATCGGTAAACAGGTTGTTATGGTTTCTCGTGAATAA

***At*DBR**
MGSSHHHHHHSSGLVPRGSHMTATNKQVILKDYVSGFPTESDFDFTTTTVELRVPEGTNSVLVKNLYLSCDPYMRIRMGKPDP
STAALAQAYTPGQPIQGYGVSRIIESGHPDYKKGDLLWGIVAWEEYSVITPMTHAHFKIQHTDVPLSYYTGLLGMPGMTAYAGF
YEVCSPKEGETVYVSAASGAVGQLVGQLAKMMGCYVVGSAGSKEKVDLLKTKFGFDDAFNYKEESDLTAALKRCFTNGIDIYFE
NVGGKMLDAVLVNMNMHGRIAVCGMISQYNLENQEGVHNLSNIIYKRIRIQGFVVSDFYDKYSKFLEFVLPHIREGKITYVEDV
ADGLEKAPEALVGLFHGKNVGKQVVVVARE

ATGGGCAGCAGCCATCATCATCATCATCACAGCAGCGGCCTGGTGCCGCGCGGCAGCCATATGACTGCGACCAACAAACA
GGTTATCCTGAAAGACTACGTTTCTGGTTTCCCGACCGAATCTGATTTCGATTTCACCACCACCACCGTTGAACTGCGTGTT
CCGGAAGGTACTAACTCTGTTCTGGTTAAAAACCTGTACCTGTCTTGCGATCCGTACATGCGCATCCGTATGGGTAAACCG
GACCCGTCTACCGCTGCTCTGGCTCAGGCGTACACTCCGGGTCAGCCGATCCAGGGTTACGGTGTGTCTCGTATCATCGAA
TCCGGCCACCCGGACTACAAAAAAGGTGACCTGCTGTGGGGTATCGTTGCGTGGGAAGAATACTCTGTTATCACCCCGAT
GACCCACGCGCACTTCAAAATCCAGCATACCGACGTTCCGCTGTCTTACTACACCGGCCTGCTGGGTATGCCGGGCATGAC
CGCGTACGCAGGTTTCTACGAAGTTTGCTCTCCGAAAGAAGGTGAAACCGTTTACGTTTCTGCGGCATCCGGTGCTGTTGG
TCAGCTGGTAGGTCAGCTGGCTAAAATGATGGGCTGCTACGTTGTTGGTAGCGCTGGTTCTAAAGAAAAAGTTGACCTGC
TGAAAACCAAATTCGGTTTCGATGACGCGTTCAACTACAAAGAAGAATCTGACCTGACCGCGGCGCTGAAACGTTGCTTCA
CCAACGGTATCGACATCTACTTCGAAAACGTTGGTGGTAAAATGCTGGACGCTGTTCTGGTTAACATGAACATGCACGGCC
GTATCGCTGTTTGTGGTATGATCTCTCAGTACAACCTGGAAAACCAGGAAGGTGTTCACAACCTGTCTAACATCATCTACAA
ACGTATCCGTATCCAGGGTTTCGTTGTTTCTGACTTCTACGATAAATACTCTAAATTCCTGGAGTTCGTTCTGCCGCACATCC
GTGAAGGTAAAATCACCTACGTTGAAGATGTTGCGGACGGTCTGGAAAAAGCGCCGGAAGCGCTGGTTGGTCTGTTCCAC
GGTAAAAACGTTGGTAAACAGGTTGTGGTTGTTGCGCGTGAATAAC

***Ri*DBR**
MGSSHHHHHHSSGLVPRGSHMASGGEMQVSNKQVIFRDYVTGFPKESDMELTTRSITLKLPQGSTGLLLKNLYLSCDPYMRAR
MTNHHRLSYVDSFKPGSPIIGYGVARVLESGNPKFNPGDLVWGFTGWEEYSVITATESLFKIHNTDVPLSYYTGLLGMPGMTAY
AGFYEICSPKKGETVYVSAASGAVGQLVGQFAKLTGCYVVGSAGSKEKVDLLKNKFGFDEAFNYKEEADLDAALRRYFPDGIDIYF
ENVGGKMLDAVLPNMRPKGRIAVCGMISQYNLEQPEGVRNLMALIVKQVRMEGFMVFSYYHLYGKFLETVLPYIKQGKITYVE
DVVDGLDNAPAALIGLYSGRNVGKQVVVVSRE

ATGGGCAGCAGCCATCATCATCATCATCACAGCAGCGGCCTGGTGCCGCGCGGCAGCCATATGGCTTCTGGTGGTGAAAT
GCAGGTTTCCAACAAACAGGTTATCTTCCGTGACTACGTTACCGGTTTCCCGAAAGAATCTGATATGGAACTGACCACCCG
TTCTATCACCCTGAAACTGCCGCAGGGTTCTACCGGTCTGCTGCTGAAAAACCTGTACCTGTCTTGTGACCCGTATATGCGT
GCTCGTATGACTAACCACCACCGTCTGTCTTATGTTGATTCTTTCAAACCGGGTTCTCCGATCATCGGCTACGGTGTTGCTC
GTGTGCTGGAATCTGGTAACCCGAAATTCAACCCAGGTGACCTGGTTTGGGGCTTCACTGGCTGGGAAGAATACTCTGTTA
TCACCGCGACCGAATCTCTGTTCAAAATCCACAACACCGACGTGCCGCTGAGCTACTACACCGGTCTGCTGGGCATGCCGG
GCATGACCGCTTACGCAGGTTTCTACGAAATTTGCAGCCCGAAAAAAGGTGAAACCGTTTACGTGAGCGCAGCATCTGGC
GCAGTTGGTCAGCTGGTTGGTCAGTTCGCGAAACTGACCGGTTGCTACGTGGTTGGCTCCGCAGGTTCTAAAGAAAAAGT
TGATCTGCTGAAAAACAAATTCGGTTTCGATGAAGCATTCAACTACAAAGAAGAAGCGGACCTGGATGCGGCTCTGCGTC
GTTACTTCCCGGACGGTATCGACATCTACTTCGAAAACGTTGGTGGTAAAATGCTGGACGCGGTTCTGCCGAACATGCGTC
CGAAAGGTCGTATTGCTGTTTGCGGTATGATCTCTCAGTACAACCTGGAACAACCGGAAGGCGTTCGTAACCTGATGGCG
CTGATCGTGAAACAGGTTCGTATGGAAGGTTTCATGGTTTTCTCTTACTACCACCTGTATGGTAAATTCCTGGAAACCGTAC

TGCCGTACATCAAACAGGGTAAAATCACCTACGTTGAAGATGTTGTTGATGGTCTGGATAACGCTCCGGCTGCGCTGATCG
GTCTGTACTCTGGCCGTAACGTTGGTAAACAGGTTGTTGTTGTTAGCCGTGAATAA

***Bs*GDH E170K/Q252L**
MGSSHHHHHHSSGLVPRGSHMYPDLKGKVVAITGAASGLGKAMAIRFGKEQAKVVINYYSNKQDPNEVKEEVIKAGGEAVVV
QGDVTKEEDVKNIVQTAIKEFGTLDIMINNAGLENPVPSHEMPLKDWDKVIGTNLTGAFLGSREAIKYFVENDIKGNVINMSSV
HEVIPWPLFVHYAASKGGIKLMTKTLALEYAPKGIRVNNIGPGAINTPINAEKFADPKQKADVESMIPMGYIGEPEEIAAVAAWL
ASKEASYVTGITLFADGGMTLYPSFQAGRG

ATGGGCAGCAGCCATCATCATCATCATCACAGCAGCGGCCTGGTGCCGCGCGGCAGCCATATGTATCCGGATTTAAAAGG
AAAAGTCGTCGCTATTACAGGAGCTGCTTCAGGGCTCGGAAAGGCGATGGCCATTCGCTTCGGCAAGGAGCAGGCAAAA
GTGGTTATCAACTATTATAGTAATAAACAAGATCCGAACGAGGTAAAAGAAGAGGTCATCAAGGCGGGCGGTGAAGCTG
TTGTCGTCCAAGGAGATGTCACGAAAGAGGAAGATGTAAAAAATATCGTGCAAACGGCAATTAAGGAGTTCGGCACACTC
GATATTATGATTAATAATGCCGGTCTTGAAAATCCTGTGCCATCTCACGAAATGCCGCTCAAGGATTGGGATAAAGTCATC
GGCACGAACTTAACGGGTGCCTTTTTAGGAAGCCGTGAAGCGATTAAATATTTCGTAGAAAACGATATCAAGGGAAATGT
CATTAACATGTCCAGTGTGCACGAAGTGATTCCTTGGCCGTTATTTGTCCACTATGCGGCAAGTAAAGGCGGGATAAAGCT
GATGACAAAGACATTAGCGTTGGAATACGCGCCGAAGGGCATTCGCGTCAATAATATTGGGCCAGGTGCGATCAACACGC
CAATCAATGCTGAAAAATTCGCTGACCCTAAACAGAAAGCTGATGTAGAAAGCATGATTCCAATGGGATATATCGGCGAA
CCGGAGGAGATCGCCGCAGTAGCAGCCTGGCTTGCTTCGAAGGAAGCCAGCTACGTCACAGGCATCACGTTATTCGCGGA
CGGCGGTATGACACTCTATCCTTCATTCCAGGCAGGCCGCGGTTAA

Enzyme activity

The specific activity of all enzymes used was measured with a model substrate (**Table S2**), by monitoring NADPH consumption at 340 nm (ε_{340} = 6.22 $M^{-1}cm^{-1}$) on a Cary 60 spectrophotometer.

Table S2. Enzyme specific activity on model substrates and substrate scope.

**Substrate**		**Enzyme**	**Spec. act. (U/mg)**
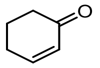	cyclohexenone	OYE2	3.4 ± 0.1
		OYE3	2.6 ± 0.2
		OYE3 Y197F	0.05 ± 0.00
		GluER	6.7 ± 0.5
		*Ts*OYE[a]	7.0 ± 0.3
		YqjM	8.3 ± 0.4
		*Nt*DBR	n.d.
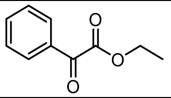	ethyl benzoylformate	OYE3	n.d.
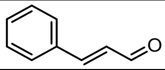	*trans*-cinnamaldehyde	*Nt*DBR	2.2 ± 0.1
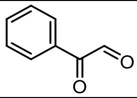	phenylglyoxal	*Nt*DBR	0.06 ± 0.01
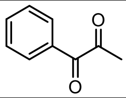	1-phenyl-1,2-propanedione	*Nt*DBR	n.d.

Conditions: 50 mM MOPS-NaOH pH 7, 10 mM substrate (from 0.5 M DMSO stock), 0.2 mM NADPH, 10 U/mL glucose oxidase, 20 mM Glc, enzyme concentrations in order top to bottom: 0.61 µM, 0.87 µM, 0.25 µM, 0.09 µM, 0.43 µM, 0.56 µM, 2.63 µM, 1.8 µM, 0.27 µM , 2.61 µM, 4.11 µM, 30 °C. n.d. represents not detected. [a] *N*-terminal His-tagged *Ts*OYE has a slightly lower specific activity than the published *Ts*OYE wt without His-tag (12 U/mg). n.d. = not detected.

Bioconversions

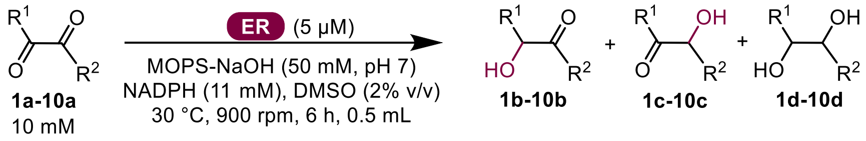

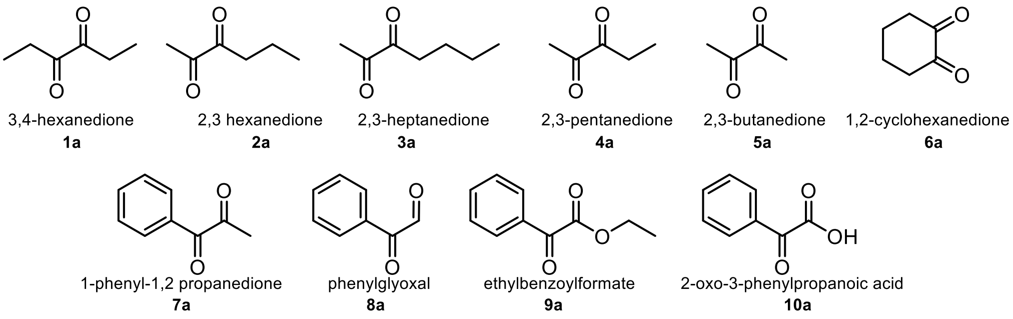

Figure S12. Substrate scope screened.

Table S3. Screening of six EREDs on substrates **1a-10a**

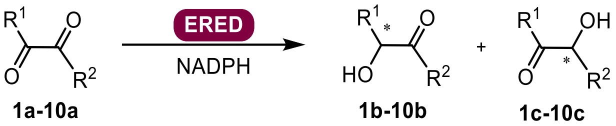

Product		Conversion (%)					
		GluER	OYE3	OYE2	YqjM	*Ts*OYE	*Nt*DBR
**1b**	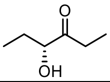	8<br>*ee*: 92%	0	0	0	0	0
**2b**	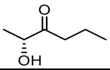	19<br>*ee*: 83%	0	0	0	0	0
**3b**	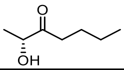	17<br>*ee*: 67%	0	0	0	0	0
**4b**	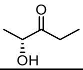	<1[a]	0	0	0	0	0
**5b**	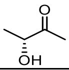	<1[a]	0	0	0	0	0
**6b**	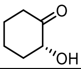	0	0	2	0	2	0
**7b[b]**	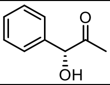	37<br>*ee*: 79%	91<br>*ee*: >99.9%	79<br>*ee*: 99%	2	14<br>*ee*: 74%	6<br>*ee*: 21%
**7c[b]**	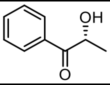	28<br>*ee*: 28%	0	0	0	0	<1
**8c**	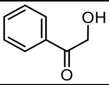	93	86	62	55	47	30
**9b**	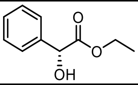	0	0	0	0	0	0
**10b**	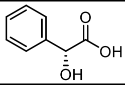	0	0	0	0	0	0

Conditions: 50 mM MOPS-NaOH pH 7, 5 µM ERED, 11 mM NADPH, 10 mM compound **1a-10a** from a 0.5 M DMSO stock (2% v/v), 0.5 mL volume, 6 h, 30 °C, 900 rpm, duplicated experiments, measured on GC. Positive *ee* represents (*R*), negative (*S*). [a] traces of product could be detected however large mass balance issues prevented accurate conversions. [b] measured on HPLC.

Table S4. ERED screening of 1-phenyl-1,2-propanedione **7a**.

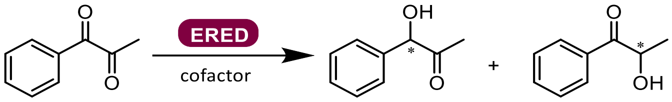

7a **7b** **7c**

class	ERED	7a	7b		7c		Conv.
		%	%	*ee* (%)[a]	%	*ee* (%)[a]	%
Ia	XenB	25.9	53.4	54	20.6	-100	74
	PETNR	73.9	26.1	8	0		26
Ib	LeOPR1	6.3	92	82	1.7	-100	94
Ic	GluER	34.8	37.1	79	28	28	65
	NCR	85.3	14.7	37	0		15
	NerA	83.2	13.6	85	3.2	-100	17
II	OYE3	8.9	91.1	100	0		91
	OYE2	20.9	79.1	99	0		79
	EPB1	39.7	60.3	84	0		60
III	*Gk*OYE[b]	82.2	11.3	49	6.6	-83	18
	*Ts*OYE	85.8	14.2	74	0		14
	XenA	82.9	17.1	91	0		17
	*T*OYE	94.2	5.8	81	0		6
	YqjM	98.3	1.7	60	0		2
JM	Ene-101	6.9	82.3	89	10.8	-89	93
	Ene-102	23.5	47.9	79	28.6	-99	76
	Ene-103	22.1	52.4	70	25.6	-97	78
	Ene-105	32.5	32.7	55	34.9	-99	68
	Ene-107	13.1	72.6	79	14.3	-99	87
	Ene-108	6.6	64.6	60	28.8	-99	93
	Ene-109	34.6	38.4	68	27	-100	65
DBR	*At*DBR	90.3	9.5	38	0.2		10
	*Ri*DBR	91.9	7.8	47	0.3		8
	*Nt*DBR	94	5.5	21	0.5		6
no ER	control	100					0

Conditions: 50 mM MOPS-NaOH pH 7, 5 µM ERED, 11 mM NADPH, 10 mM **7a** 1-phenyl-1,2-propanedione in 0.5 M stock in DMSO, 2% v/v DMSO , 0.5 mL volume, 6 h, 30 °C, 900 rpm, duplicated experiments. [a] positive represents (*R*), negative represents (*S*), [b] heat purified. Measured on HPLC normal phase column **E** CHIRALCEL OD, 9:1 heptane:isopropyl alcohol (IPA).

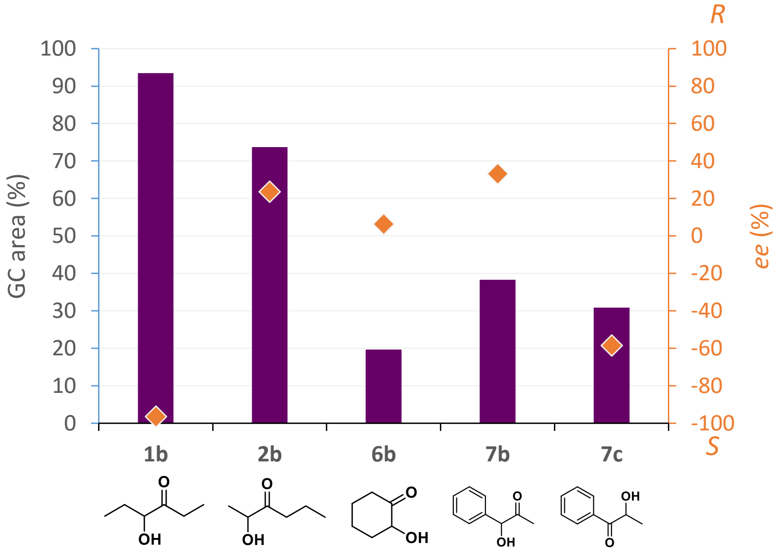

Figure S13. Dicarbonyl reduction with *Bs*GDH. Reaction conditions: 50 mM MOPS-NaOH pH 7, 10 U/mL *Bs*GDH, 0.2 mM $NADP^+$, 30 mM Glc (**1b**, **2b**, **6b**), 20 mM Glc (**7b**,**c**), 10 mM substrate, 1% v/v DMSO, 1 mL volume, 6 h, 30 °C, 900 rpm. Analysis on GC. Note the GC accuracy for **7b** (see section GC Accuracy) is questionable, such that the *ee* may be higher than what was measured, also the isomerization to **7c** may be less than what is shown.

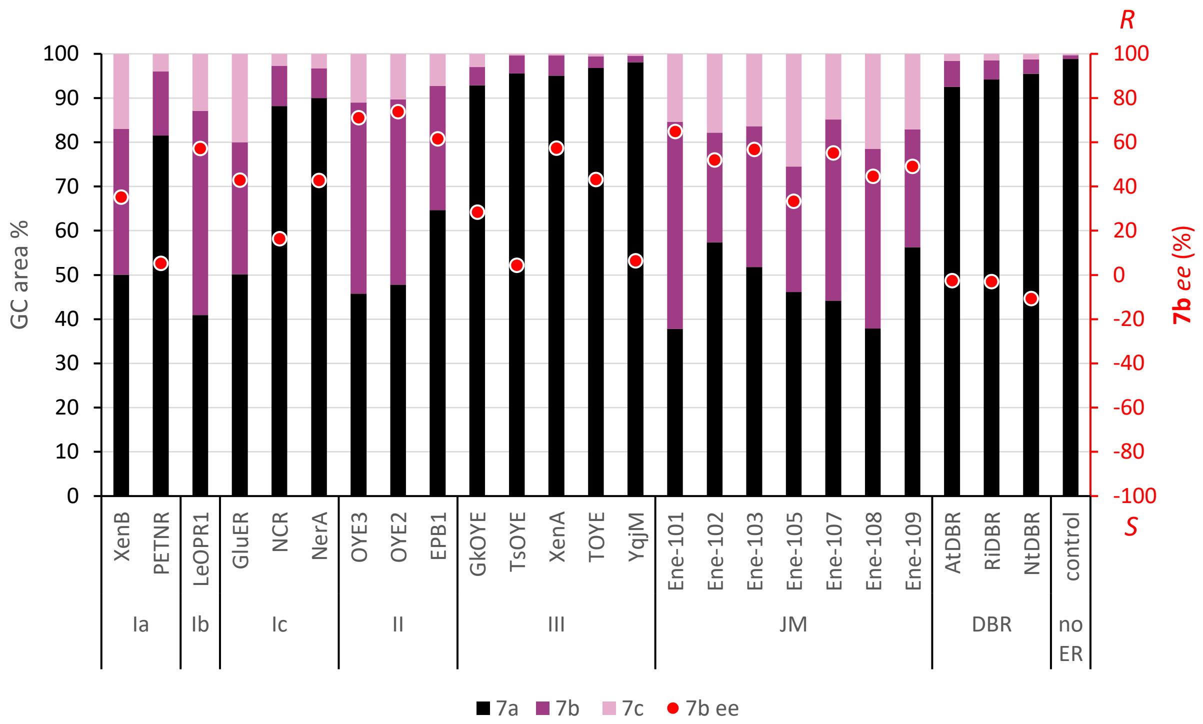

Figure S14. GC analysis for ERED screening of 1-phenyl-1,2-propanedione **7a**. Conditions: 50 mM MOPS-NaOH pH 7, 5 µM ERED, 11 mM NADPH, 10 mM 1-phenyl-1,2-propanedione in 0.5 M DMSO, 2% v/v DMSO, 0.5 mL volume, 6 h, 30 °C, 900 rpm, duplicated experiments with GC analysis. *Gk*OYE was heat purified.

Kinetic parameters

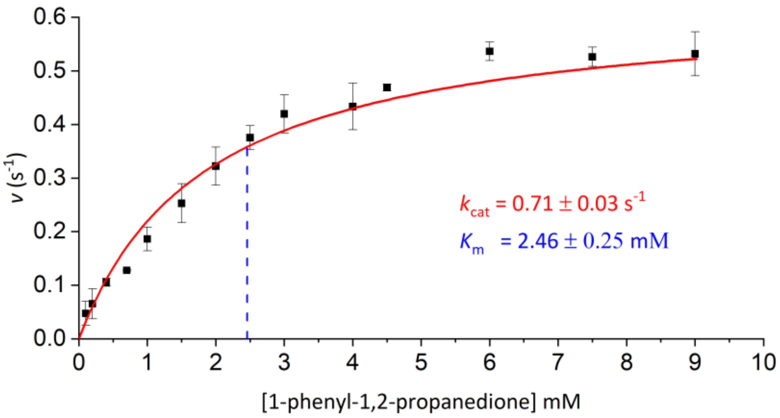

Figure S15. Kinetic parameters of the OYE3-catalysed reduction of 1-phenyl-1,2-propanedione **7a**. Conditions: anaerobic (Coy chamber), in a UV cuvette, 50 mM MOPS-NaOH pH 7 buffer, 10 µM OYE3, 1-phenyl-1,2-propanedione **7a** (from 1 M DMSO stock), 0.2 mM NADPH, 1 mL volume, measured at 340 nm at 25 °C. k_{cat}/K_m = 0.29 mM^{-1} s^{-1}.

Analytic methods

EPR spectroscopy

EPR spectra were recorded using a Bruker EMXplus X-band spectrometer equipped with a helium-flow cryostat operating at a temperature of 20K.[3,4] EPR spectroscopy was carried out for the reaction with OYE3. Both the reaction and blank samples show no clear radical signal. There are a few very small broad features, which are not clearly attributable, and are virtually identical in the blank (enzyme only) and reaction samples (**Figure S16b**). The TEMPO sample at 1.5 times the concentration of OYE3 showed

a very clear radical signal (**Figure S16a**) using the same settings as for the OYE samples. 4-hydroxy-TEMPO showed only a broad isotropic signal centered at g = 2.00 at low temperature (20K).[5] At room temperature TEMPO and hydroxy-TEMPO have the very characteristic nitroxide radical three-line pattern.[6]

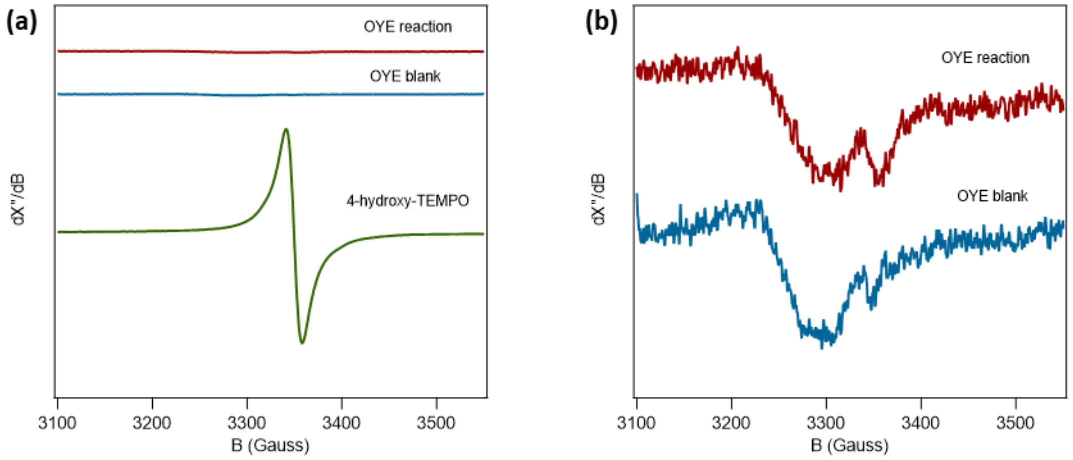

Figure S16. EPR spectroscopy of OYE3 conversion of 1-phenyl-1,2-propanedione with NADPH. a) overview of the conversion and control samples which shows no significant radical formation as compared to the 4-hydroxyTEMPO control sample, b) blowup of the reaction and blank samples showing they are highly similar. Samples: OYE Blank: 20 µM OYE3, 11 mM NADPH, 50 mM MOPS-NaOH pH 7; OYE Reaction: 1.2 min reaction; 20 µM OYE3, 11 mM NADPH, 50 mM MOPS-NaOH pH 7, substrate: 10 mM 1-phenyl-1,2-propanedione; 4-hydroxy-TEMPO 30 µM 4-hydroxy-TEMPO in water. EPR conditions: Microwave frequency, 9.4096 GHz; microwave power, 2 mW; modulation frequency, 100 kHz; modulation amplitude, 10 Gauss; temperature, 20 K. The spectra were the result of 4x averaging.

A 10-fold higher OYE3 concentration did not result in a significant increase of a radical signal (not shown). Although small signals close to the background could be indicative of a minor radical species, similar signals were observed in the OYE blank sample. Based on the 4-hydroxy-TEMPO signal we can assume that there was less than 0.15% radical in the OYE samples. In conclusion, no significant radical species in OYE3 during the turnover (steady state) was observed, and any radical species was at least below circa 0.15% enzyme concentration.

GC analyses

The following columns and conditions were used: **Column A**: Agilent J&W CP-Sil 8 CB (25 m × 0.25 mm × 1.20 µm), nitrogen carrier gas, injector temp set to 340 °C, detector set to 360 °C. **Column B**: Agilent CP-Chirasil-Dex CB (25 m × 0.32 mm × 0.25 µm), helium carrier gas, injector temp set to 250 °C, detector set to 250 °C. **Column C**: Macherey-Nagel Hydrodex™ β-TBDM (50 m × 0.25 mm × 0.15 µm), helium carrier gas, injection of 1 µL sample at 250 °C, detector set to 275 °C. Instrument oven method written as: initial temperature (°C) / hold time (min) / rate (°C/min) / temperature (°C) / hold time (min) / rate (°C/min) / temperature (°C) / hold time (min).

Table S5. GC column oven methods and compound retention times.

Column	Oven method	Compound		Ret. time (min)
**A**	**(1)** 50/5/20/345/1	**1a**	3,4-hexanedione	6.7
**CP-Sil 8**	split 50	**1b**	4-hydroxyhexan-3-one	8.1
**CB**	initial linear velocity 30.0	**2a**	2,3-hexanedione	6.5
	cm/s	**2b**	2-hydroxyhexan-3-one	8.6
		**3a**	2,3-heptanedione	7.9
		**4a**	2,3-pentanedione	5.1
		**4b**	2-hydroxypentan-3-one	7.1
		**7a**	1-phenyl-1,2-propanedione	15.2
		**7b**	phenylacetylcarbinol	15.8
		**7c**	2-hydroxy-1-phenylpropan-1-one	16.2
		**8a**	phenylglyoxal	10.2

**B Chirasil-Dex CB**	**(2)** 80/5/20/220/1 split 50 initial linear velocity 30.0 cm/s	**1a**	3,4-hexanedione	3.5
		**1b**	(*R*)-4-hydroxyhexan-3-one	7.2
		**1b**	(*S*)-4-hydroxyhexan-3-one	7.6
		**1d**	(3*S*,4*S*)-hexane-3,4-diol	9.3
		**1d**	(3*R*,4*R*)-hexane-3,4-diol	9.4
		**1d**	(3*R*,4*S*)-hexane-3,4-diol	9.6
		**2a**	2,3-hexanedione	3.3
		**2b**	(*R*)-2-hydroxyhexan-3-one	7.2
		**2b**	(*S*)-2-hydroxyhexan-3-one	7.4
		**3a**	2,3-heptanedione	5.1
		**3b**	(*R*)-2-hydroxyheptan-3-one	8.4
		**3b**	(*S*)-2-hydroxyheptan-3-one 2,3-	8.5
		**4a**	pentanedione	2.5
		**4b**	(*R*)-2-hydroxypentan-3-one	5.7
		**4b**	(S)-2-hydroxypentan-3-one	6.1
		**5a**	2,3-butanedione	2.5
**B**	**(3)** 70/2/15/130/5/15/225 /2 split 100 initial linear velocity 30.0 cm/s	**1a**	3,4-hexanedione	3.9
		**1b**	(*R*)-4-hydroxyhexan-3-one	6.3
		**1b**	(*S*)-4-hydroxyhexan-3-one	6.5
		**1d**	(3*R*,4*R*)-hexane-3,4-diol	9.3
		**1d**	(3*R*,4*S*)-hexane-3,4-diol	9.8
**B**	**(4)** 110/0/3/125/0/25/150 /0, Split 50 ,initial linear velocity 30.0 cm/s	**6a**	1,2-cyclohexanedione	5.0
		**6b**	(*S*)-2-hydroxycyclohexan-1-one	3.3
		**6b**	(*R*)-2-hydroxycyclohexan-1-one	3.6
**C Hydrodex β-TBDM**	**(5)** 100/2/5/160/6/20/245 /1 split 50, initial linear velocity 38 cm/s	**7a**	1-phenyl-1,2-propanedione	12.4
		**7c**	(*R*)-2-hydroxy-1-phenylpropan-1-one	18
		**7b**	(*R*)-phenylacetylcarbinol	18.2
		**7c**	(*S*)-2-hydroxy-1-phenylpropan-1-one	18.5
		**7b**	(*S*)-phenylacetylcarbinol	18.7
		**8a**	phenylglyoxal	10.8
		**8c**	2-hydroxyacetophenone	18.2
		**9a**	ethylbenzoylformate	18.7
		**15a**	acetophenone	11.3
		**15b**	(*R*)-1-phenylethan-1-ol	14.6
		**15b**	(*S*)-1-phenylethan-1-ol	14.8
**C**	**(6)** 50/2/5/110/0/150/0/2 0/245/1 split 50, initial linear velocity 38.0 cm/s	**2b**	(*R*)-2-hydroxyhexan-3-one	15.9
		**2b**	(*S*)-2-hydroxyhexan-3-one	16.5

Accuracy of GC measurements for product 7b

While measuring the standard **7b** on GC, we observed the formation of isomer **7c** (**Figure S17** and **Figure S18**) due to a GC-inlet-catalysed hydride shift. We noticed that after maintenance of our GCs (changing the septum and glass wool in the GC inlet liner) that the isomerization had decreased, implying a dirty septum/liner had caused this hydride shift. The NMR showed no isomer **7c** when measuring the same **7b** standard (**Figure S35**). Although the chiral and non-chiral columns on the GC measurements both showed **7c**, this was not visible from the same **7b** standard on HPLC (**Figure S25**). From our GC analysis of enzymatic reaction OYE3 with **7a** we noticed both racemization as well as isomerization (**Figure S24**), which was not seen on the HPLC (**Figure S25**), suggesting that the GC may also cause racemization, even after maintenance. It is for these reasons we used the HPLC results.

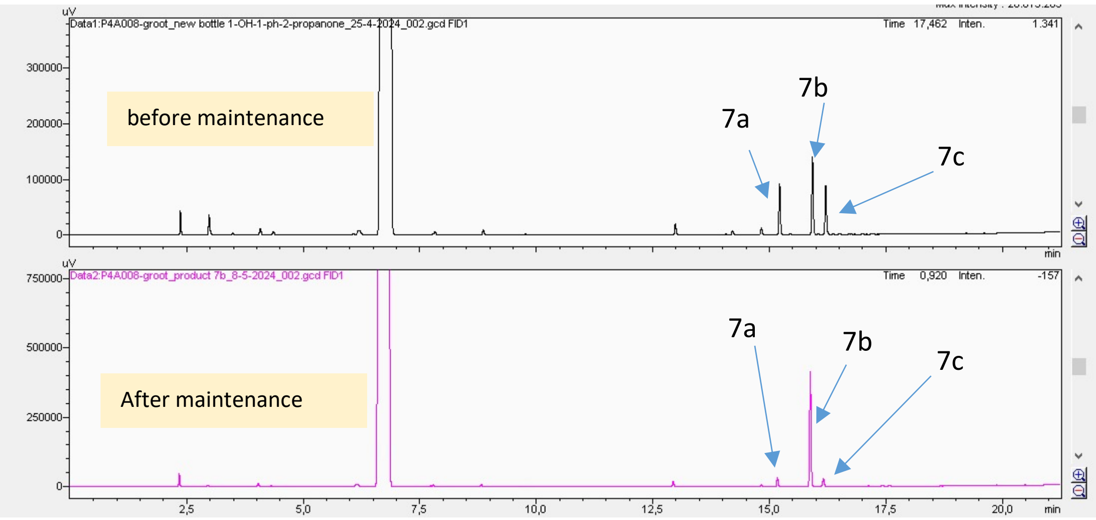

Figure S17. GC-FID chromatogram on achiral column **A** of racemic standard 1-hydroxy-1-phenylpropan-2-one **7b**. The standard **7b** showed a hydride shift isomerization to **7c** and ketone formation to **7a** prior to maintenance. Post maintenance the chromatogram showed only traces of **7a** and **7c**, which matches with the NMR analysis.

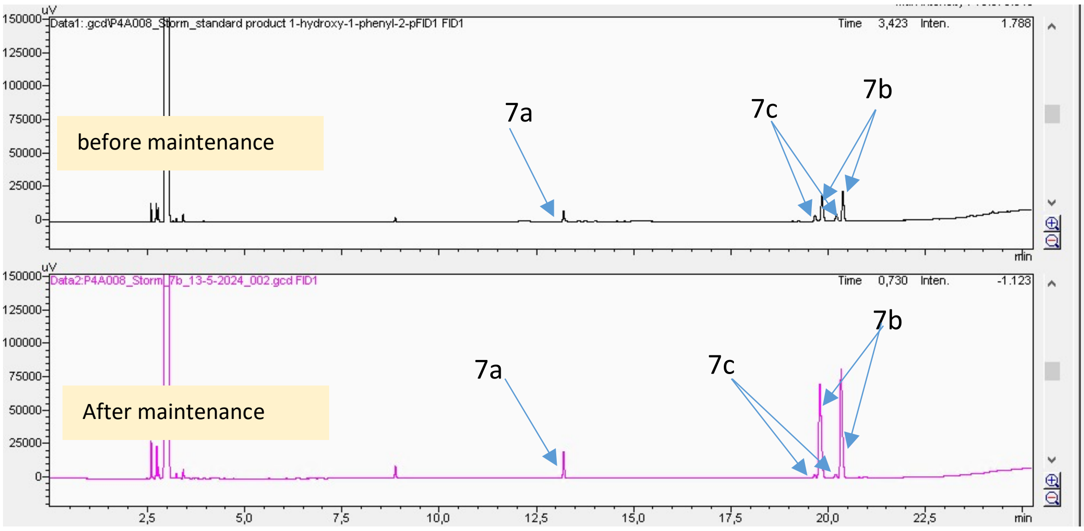

Figure S18. GC-FID chromatogram on chiral column **C** of racemic standard 1-hydroxy-1-phenylpropan-2-one **7b**. Note the retention time have been shifted to the right. The standard **7b** showed hydride shift isomerization to **7c** and ketone formation to **7a** prior to maintenance. Post maintenance the chromatogram showed traces of **7a** and **7c**, which matches with the NMR analysis.

HPLC analyses

Phenylglyoxal **8a** and benzoyl formic acid **10a** were followed by HPLC on **column D** Restek Raptor ARC-18 (150 mm × 4.6 mm × 2.7 µm). Oven temperature was 30 °C, injection volume 2 µL, flow rate of 1 mL/min.

Table S6. HPLC method

Time (min)	H_2O, TFA (0.1%)	MeCN, TFA (0.1%)
0.0	95	5
2.0	95	5
12.0	0	100
14.0	0	100
14.1	95	5
21.0	95	5

Table S7. HPLC column **D** Restek Raptor ARC-18 and compound retention times.

Compound	Ret. time (min)
mandelic acid	6.7

**8a** phenylglyoxal	6.8
**10a** benzoyl formic acid	7.1
**8b** 1-hydroxyacetophenone	7.5
benzyl alcohol	7.5
benzoic acid	8.2
benzaldehyde	9.0
**12b** benzoin	9.9
**12a** benzil	11.6
NAD^+	1.5

1-Phenyl-1,2-propanedione **7a** on normal phase HPLC 95:5 heptane:IPA with **column E** CHIRALCEL OD 0.46 cm × 25 cm, oven temperature 30 °C, injection volume 2 µL, flow rate of 1 mL/min. 0.5 mL samples were extracted with 1 mL 9:1 heptane:IPA with ~5 mg of NaCl, centrifuged for 5 min.

Table S8. HPLC column **E** CHIRALCEL OD and compound retention times.

Compound	Ret. time (min)
**7a** 1-phenyl-1,2-propanedione	4.7
**7c** (*S*)-2-hydroxy-1-phenylpropan-1-one	7.3
**7c** (*R*)-2-hydroxy-1-phenylpropan-1-one	8.2
**7b** (*S*)-phenylacetylcarbinol	10.2
**7b** (*R*)-phenylacetylcarbinol	11.6
**11a** 1-phenylbutan-1,2-dione	4.5
**11c** (*S*)-2-hydroxy-1-phenylbutan-1-one	6.2
**11c** (*R*)-2-hydroxy-1-phenylbutan-1-one	7.3
**11a** (*S*)-1-hydroxy-1-phenylbutan-2-one	8.4
**11a** (*R*)-1-hydroxy-1-phenylbutan-2-one	8.9
**13a** 1-(4-(trifluoromethyl)phenyl)propane-1,2-dione	4.8
**13b** (*R*)-1-hydroxy-1-(4-(trifluoromethyl)phenyl)propan-2-one	8.8
**13b** (*S*)-1-hydroxy-1-(4-(trifluoromethyl)phenyl)propan-2-one	9.3
**14a** 1-(4-methoxyphenyl)propane-1,2-dione	6.0
**14b** (*S*)-1-hydroxy-1-(4-methoxyphenyl)propan-2-one	13.1
**14b** (*R*)-1-hydroxy-1-(4-methoxyphenyl)propan-2-one	14.6

Column F is CHIRALCEL OB-H 0.46 cm × 25 cm, oven temperature 30 °C, injection volume 2 µL, flow rate of 0.75 mL/min. 0.5 mL samples were extracted with 1 mL 9:1 heptane:IPA with ~5 mg of NaCl, centrifuged for 5 min.

Table S9. HPLC column **F** CHIRALCEL OB-H and compound retention times.

Compound	Ret. time (min)
**13a** 1-(4-(trifluoromethyl)phenyl)propane-1,2-dione	8.7
**13b** (*S*)-1-hydroxy-1-(4-(trifluoromethyl)phenyl)propan-2-one	16.4
**13b** (*R*)-1-hydroxy-1-(4-(trifluoromethyl)phenyl)propan-2-one	17.2

HPLC-MS analyses

High-resolution mass analysis was performed using a Q Exactive™ Focus Hybrid Quadrupole-Orbitrap™ Mass Spectrometer (Thermo Scientific, Germany) connected to an Acquity M Class liquid chromatography system (Waters, UK). The chromatographic separation was performed using a 100 × 1.0 mm BEH C18 column (1.7 µm, Acquity, UPLC column) at a constant flow rate of 25 µL/min. Solvent A consisted of H_2O plus 0.1% formic acid and a solvent B was acetonitrile + 0.1% formic acid. After 2.5 min constant at 90% solvent A (10% solvent B), a linear gradient from 10% B to 80% B was applied over 22.5 min. High-resolution mass analysis was performed in positive ionization mode over a mass range of 100–500 m/z. The resolution was set to 70K, the AGC target to 1.0e6 and the maximum injection time of 75 ms. The mass spectrometric analysis included additional all ion fragmentation scans which were acquired between 75-250 m/z using a NCE of 24 and an AGC target of 3e6. Samples were diluted

1:25 with solvents A and B (mixed in starting condition ratio) before injection. An aliquot of 5 µL was injected to LC-MS analysis system. The mass spectrometric raw data were analysed manually using the Xcalibur software tool (Thermo Scientific, Germany), and after conversion to mzXML by msconvert (ProteoWizard),[7]using the GNPS dashboard https://gnps-lcms.ucsd.edu/. [8]

Chromatograms and spectra by substrate

Alcohol dehydrogenases (ADH)-catalysed reactions were carried out to assign isomer and enantiomer peaks of the hydroxyketones. *Lb*ADH from *Lactobacillus brevis* and ADH-A from *Rhodococcus ruber* were used as cell-free extracts (CFE).[9] The general reaction conditions with *Lb*ADH was 5% v/v 2-propanol, 3 U of *Lb*ADH CFE, 1 mM $MgCl_2$, 1 mM NADPH, 10 mM substrate from a 0.5 M DMSO stock. With ADH-A general reaction conditions were 5% v/v 2-propanol, 3 U of ADH-A CFE, 1 mM NADH, 10 mM substrate from a 0.5 M DMSO stock. Both reactions were in 50 mM MOPS-NaOH buffer at pH 7, 1 mL total reaction volume, shaken at 900 rpm, 30 °C for 1 hour.

Bioconversion of 3,4-hexanedione 1a

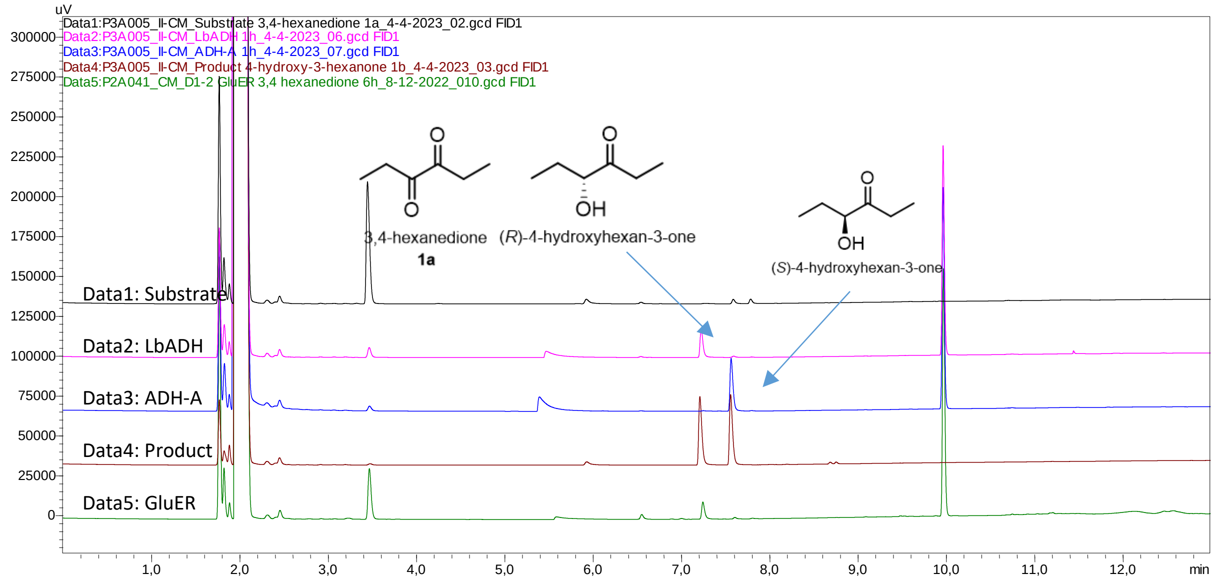

Figure S19. GC-FID chromatogram of bioconversions of 3,4-hexanedione **1a**.
Data1: commercial standard **1a**, **Data2:** reaction **1a** with *Lb*ADH for 1 h, **Data3:** reaction **1a** with ADH-A for 1 h, **Data4:** commercial standard 4-hydroxy-3-hexanone, **Data5:** reaction **1a** with GluER for 6 h. Column **B**, method (2). In literature ADH-A gives the (*S*)-4-hydroxyhexan-3-one product. [10]

Bioconversion of 2,3-hexanedione 2a

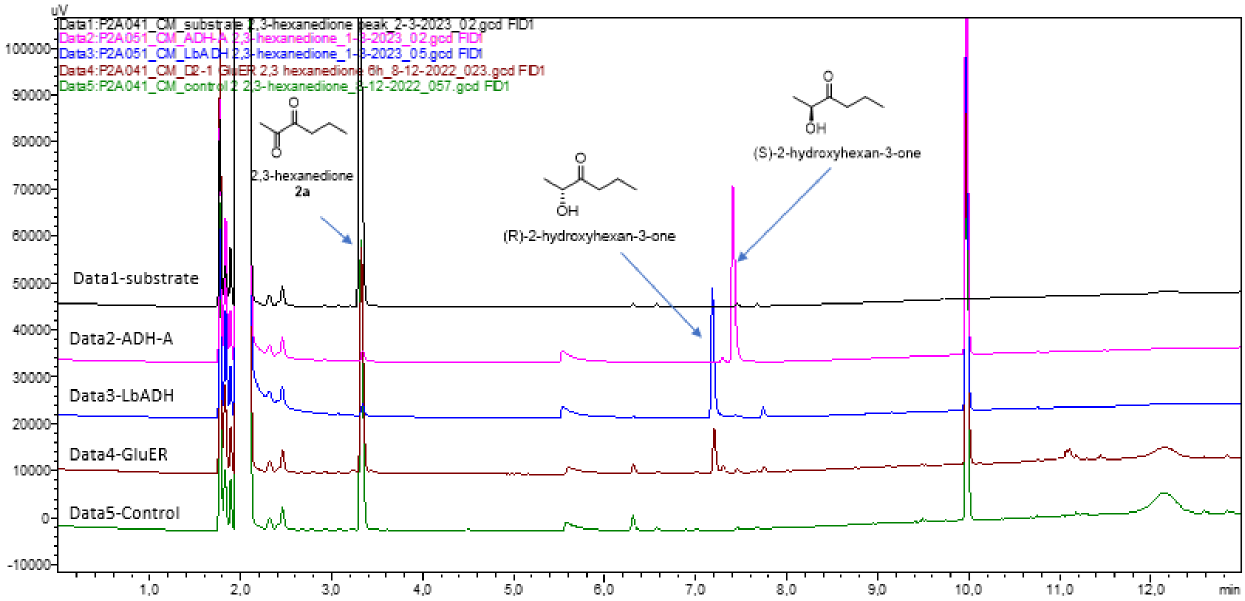

Figure S20. GC-FID chromatogram of bioconversions of 2,3-hexanedione **2a**.
Data1: commercial standard **2a**, **Data2:** reaction **2a** with ADH-A for 1 h, **Data3:** reaction **2a** with *Lb*ADH for 1 h, **Data4:** reaction **2a** with GluER for 6 h, **Data5:** Control reaction without enzyme with **2a** for 6 h. Column **B**, method (2).

For the identification of product peaks without the availability of a commercial standard, reactions with *Lb*ADH and ADH-A were performed and analysis on GC-FID, where column literature referenced Restek Rt-βDEXse (30 m × 0.25 mm × 0.25 µm)[11] is assumed to be similar to our column C (Macherey-Nagel Hydrodex-β-TBDM 50 m × 0.25mm × 0.15 µm, method (6)) and thus have the same order of retention times.

Bioconversion of 2,3-heptanedione 3a

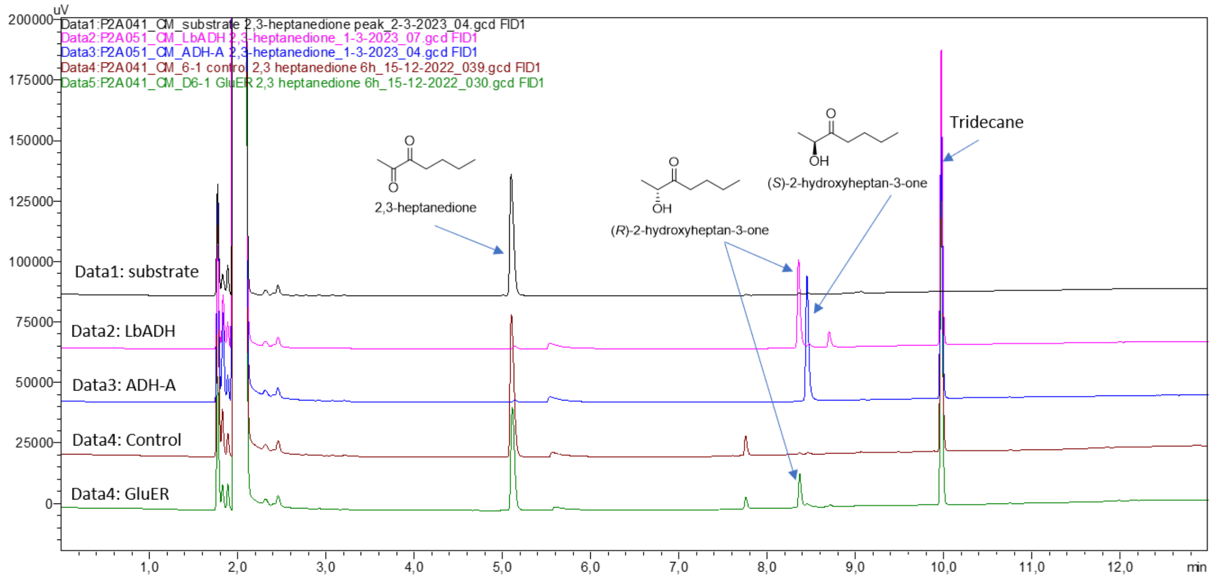

Figure S21. GC-FID chromatogram of bioconversions of 2,3-heptanedione **3a**.
Data1: commercial standard **3a**, **Data2:** reaction **3a** with *Lb*ADH for 1 h, **Data3:** reaction **3a** with ADH-A for 1 h, **Data4:** control reaction without enzyme for 6 h, **Data5:** reaction **3a** with GluER for 6 h. Column **B**, method (2).

Bioconversion of 2,3-pentanedione 4a

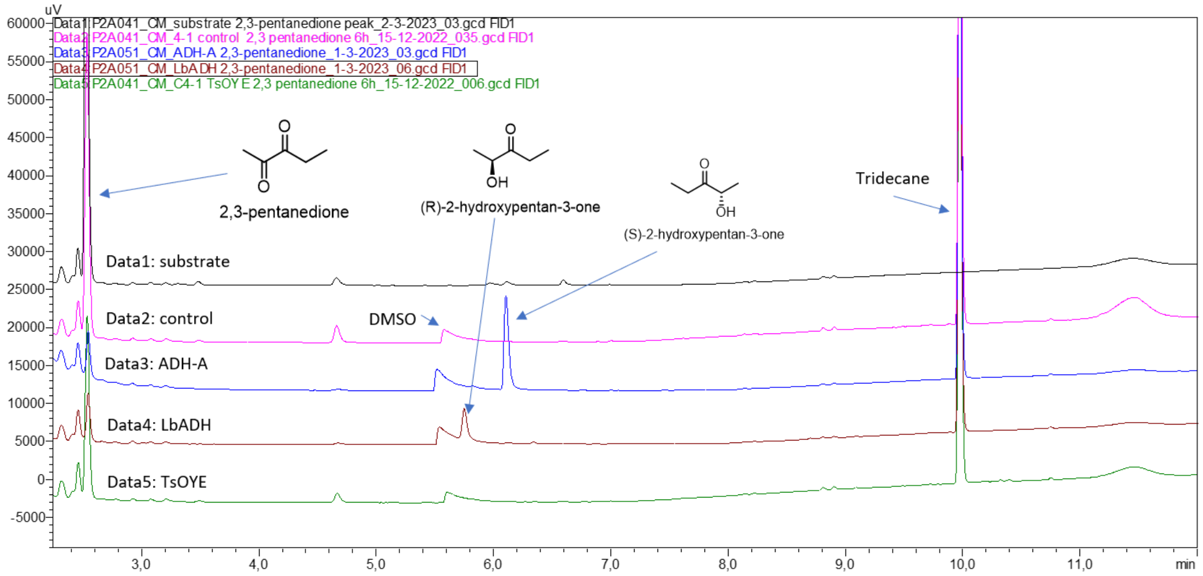

Figure S22. GC-FID chromatogram of bioconversions of 2,3-pentanedione **4a**.
Data1: commercial standard **4a**, **Data2:** control reaction without enzyme with **4a** for 6 h, **Data3:** reaction **4a** with ADH-A for 1 h, **Data4:** reaction **4a** with *Lb*ADH for 1 h, **Data5:** reaction **4a** with *Ts*OYE for 6 h. Column **B**, method (2).

Bioconversion of 1,2-cyclohexanedione 6a

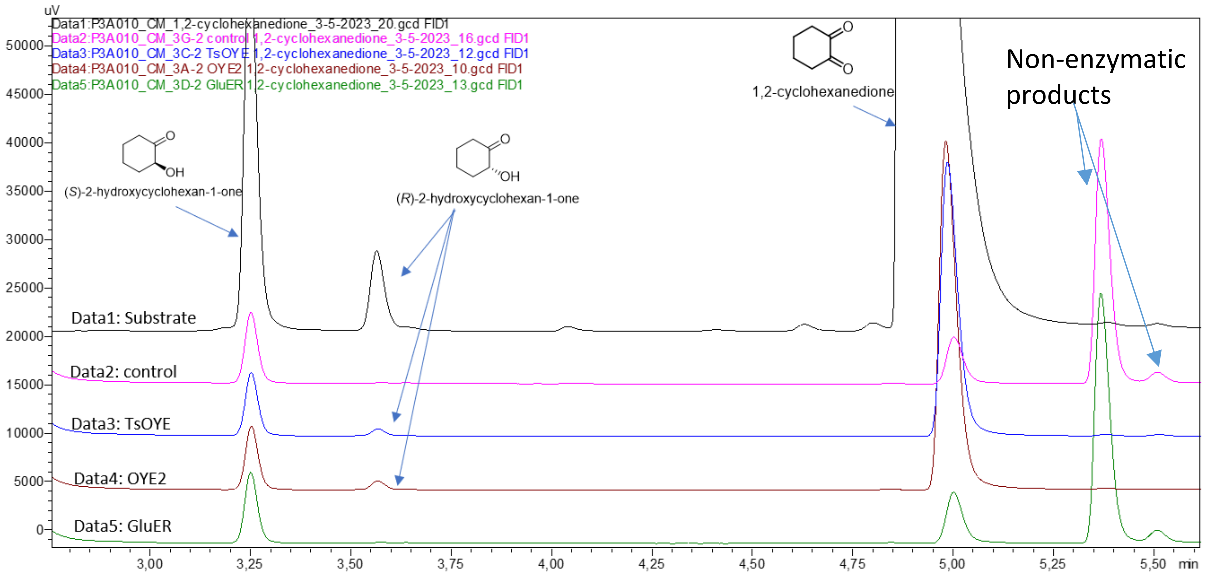

Figure S23. GC-FID chromatogram of bioconversions of 1,2-cyclohexanedione **6a**.
Data1: commercial standard **6a** (with impurities including the products, but overloaded to show clearly the peaks), **Data2:** control reaction without enzyme with **6a** for 6 h, **Data3:** reaction **6a** with *Ts*OYE for 6 h, **Data4:** reaction **6a** with OYE2 for 6 h, **Data5:** reaction **6a** with GluER for 6 h. Column **B**, method (4). Peaks for (*S*)-2-hydroxycyclohexan-1-one and (*R*)-2-hydroxycyclohexan-1-one have been compared to literature using similar column and method.[12]

Bioconversion of 1-phenyl-1,2-propanedione 7a

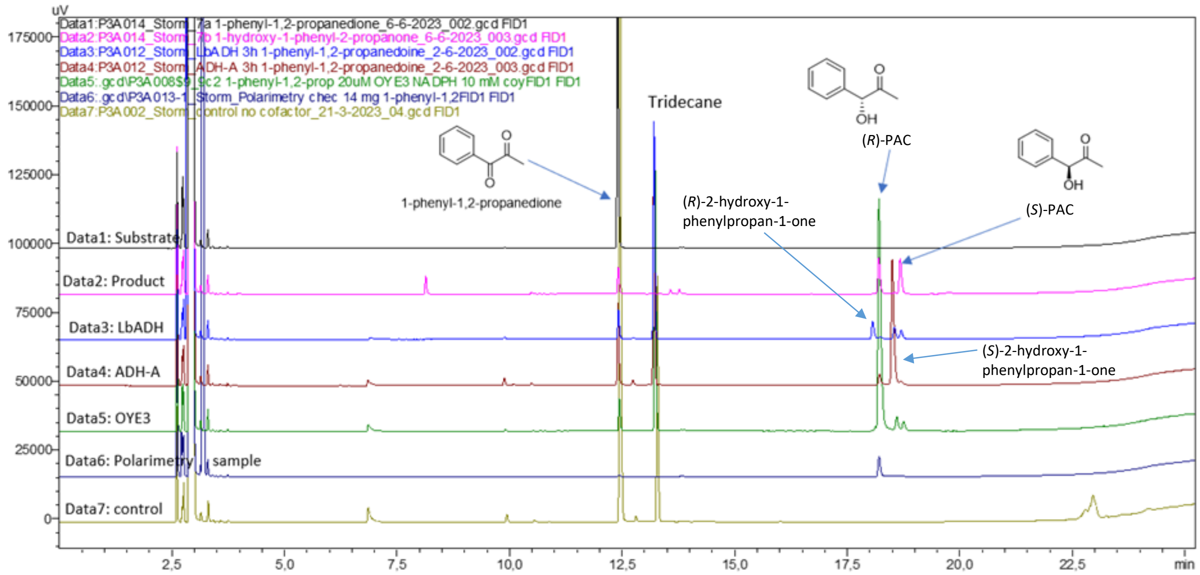

Figure S24. GC-FID chromatogram of bioconversions of 1-phenyl-1,2-propanedione **7a**.
Data1: commercial standard **7a**, **Data2:** commercial standard **7b**, **Data3:** reaction with *Lb*ADH 3 h with **7a**, **Data4:** reaction with ADH-A 3 h with **7a**, **Data5:** reaction with OYE3 (20 µM) for 4 h and 30 mM **7a**, **Data6:** Polarimetry check sample, **Data7:** control reaction without cofactor for 4 h. Column **C**, method (5). A 1.5 mL polarimetry sample in $CDCl_3$ measured angle of rotation was consistent with literature.[13] The (*R*)- and (*S*)-enantiomers for 2-hydroxy-1-phenylpropan-1-one **7c** is assumed to be in this order, where *Lb*ADH is known to be *R*-selective, and ADH-A to be *S*-selective.

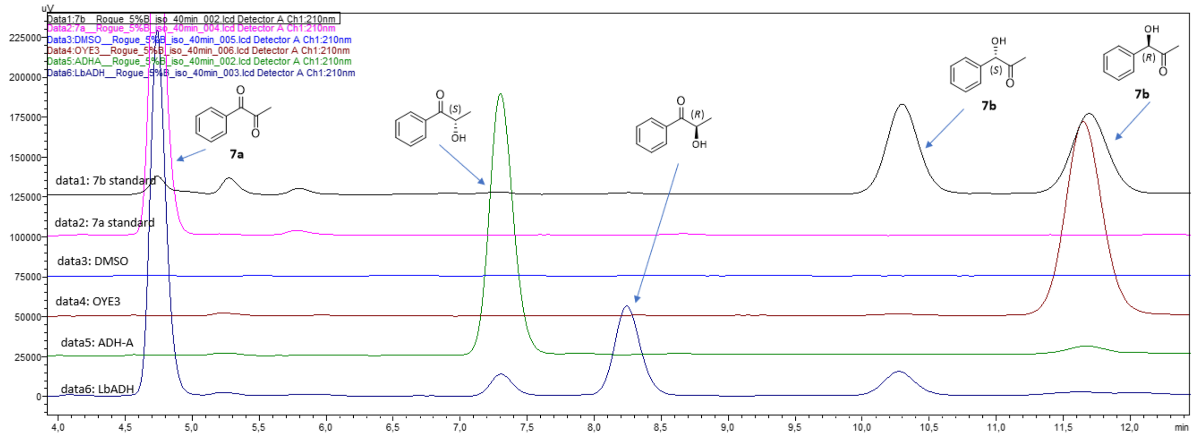

Figure S25. Chiral HPLC chromatogram of bioconversions of 1-phenyl-1,2-propanedione **7a**.
Data1: commercial standard **7b**, **Data2:** commercial standard **7a**, **Data3:** DMSO. **Data4**: anaerobic reaction with OYE3 (20 µM) for 6 h with 10 mM **7a**, 2% v/v DMSO, 11 mM NADPH, 50 mM MOPS-NaOH pH 7, 30 °C, 900 rpm. **Data5:** reaction with **7a** with ADH-A for 2.5 h. **Data6:** reaction with **7a** with *Lb*ADH for 2.5 h. Normal phase HPLC, CHIRALCEL OD, 95:5 heptane:IPA, 30 °C, 210 nm.

NMR spectra of 1-phenyl-1,2-propanedione 7a mechanistic study

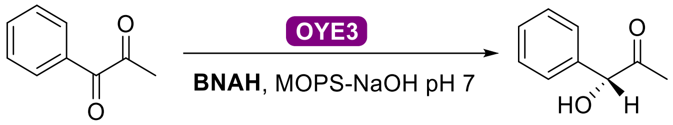

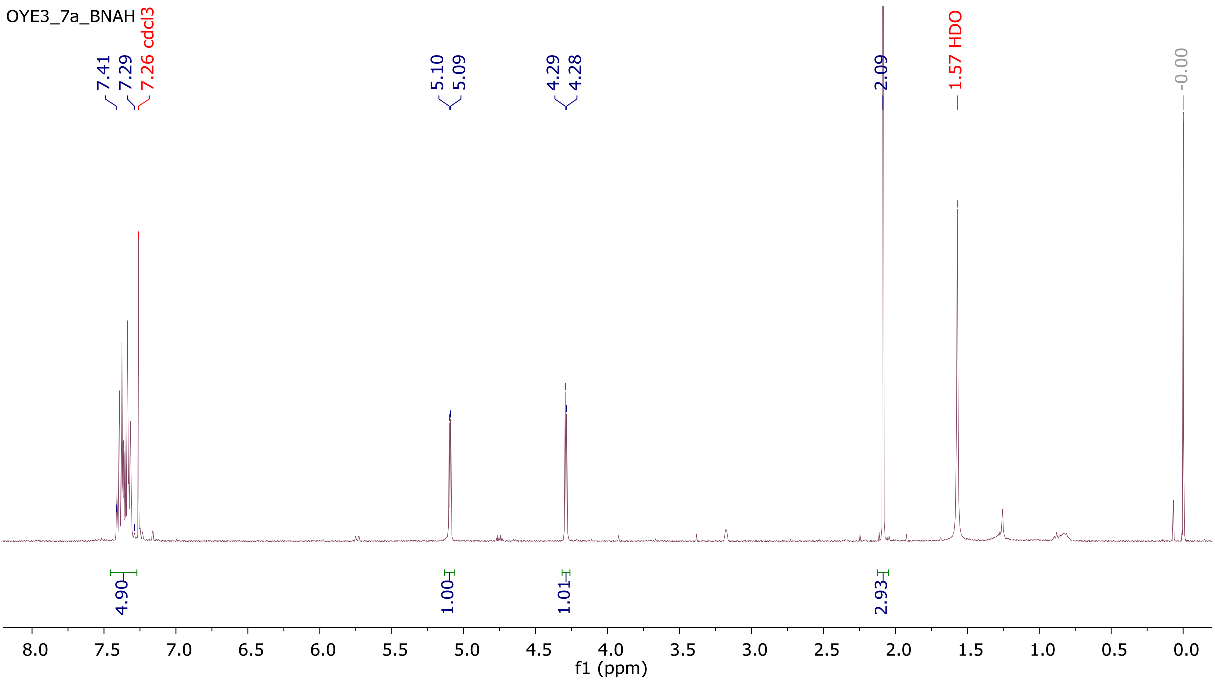

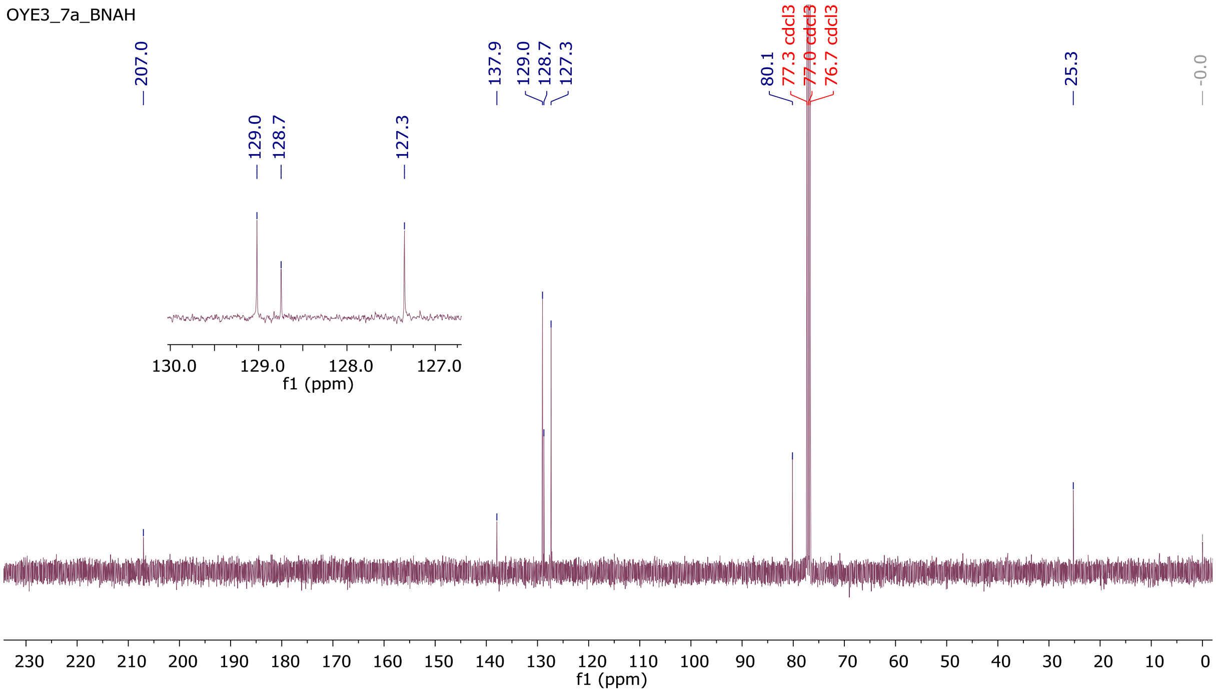

Figure S26. ^{1}H and ^{13}C NMR spectra of an OYE3-catalysed (60 µM) reaction with 30 mM 1-phenyl-1,2-propanedione **7a** and BNAH over 4.5 h in 50 mM MOPS-NaOH pH 7 buffer at 30 °C, 900 rpm, anaerobic without DMSO, extracted in $CDCl_3$. ^{1}H NMR (400 MHz, $CDCl_3$) δ 7.41–7.32 (m, 5H), 5.10 (d, *J* = 4.2 Hz, 1H), 4.29 (d, *J* = 4.2 Hz, 1H), 2.09 (s, 3H). ^{13}C NMR (101 MHz, $CDCl_3$) δ 207.1, 137.9, 129.0, 128.8, 127.4, 80.1, 25.3.

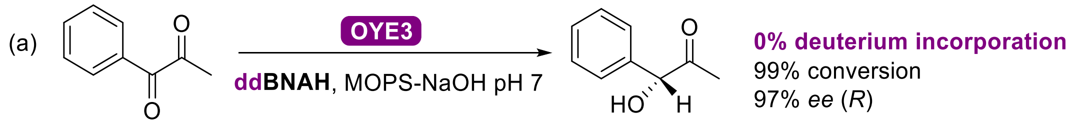

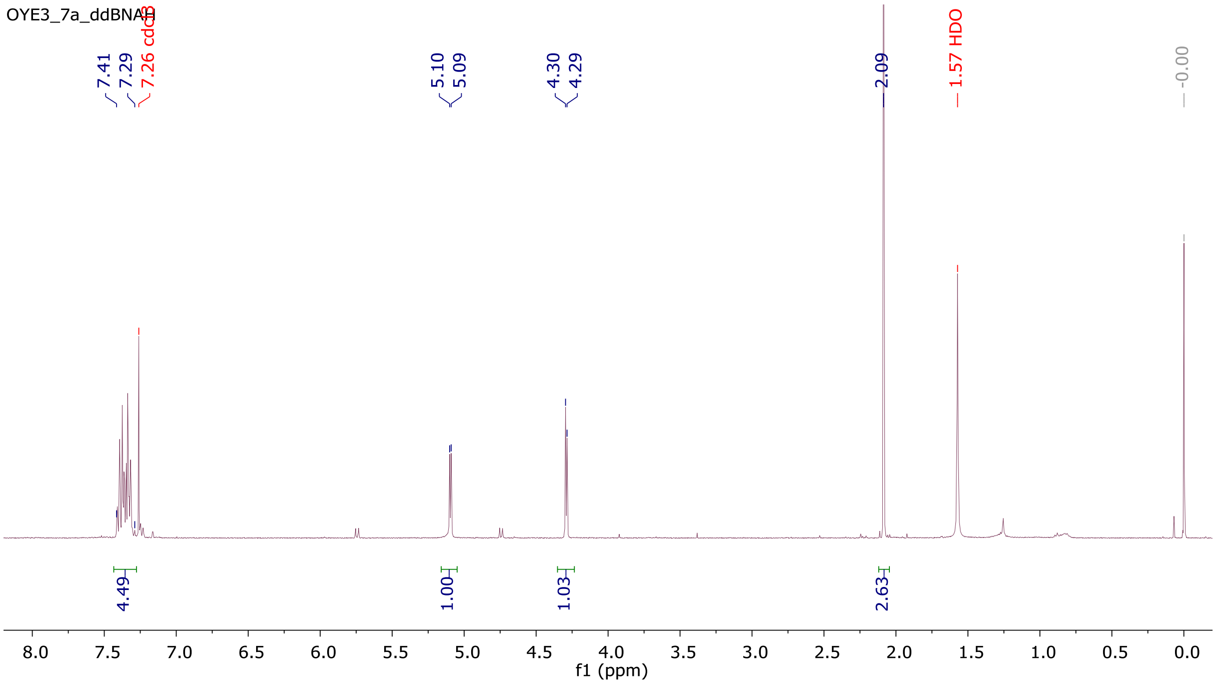

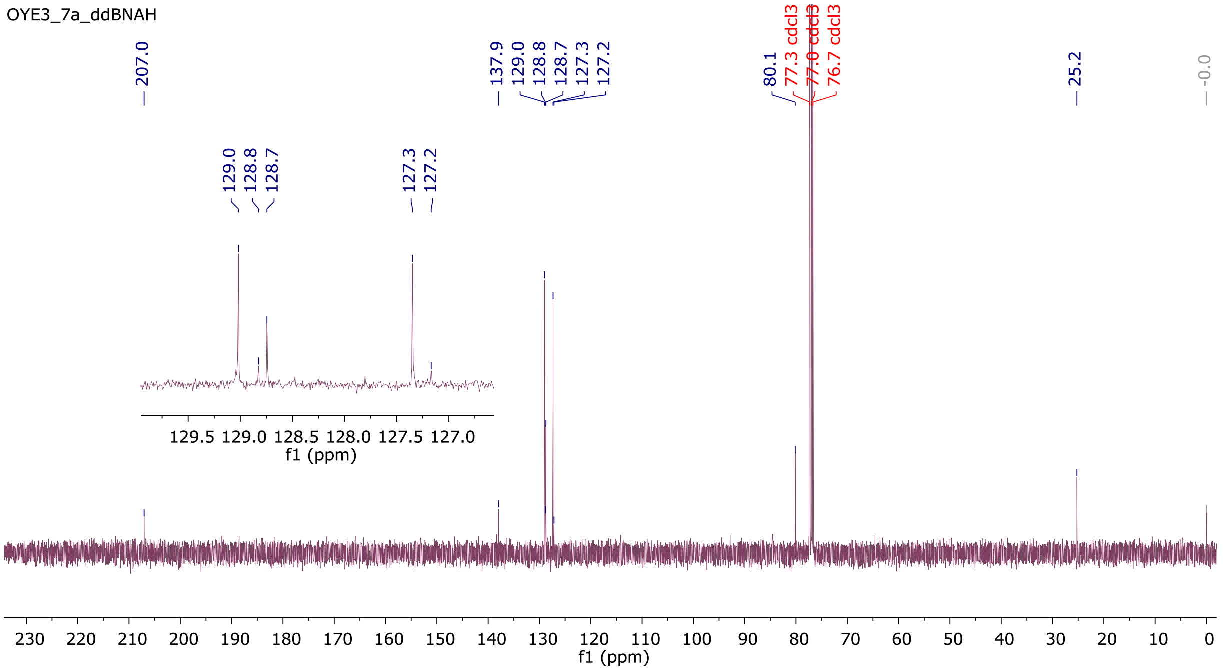

Figure S27. ^{1}H and ^{13}C NMR spectra of an OYE3-catalysed (60 µM) reaction with 30 mM 1-phenyl-1,2-propanedione **7a** and [4-^{2}H]-BNAH over 4.5 h in 50 mM MOPS-NaOH pH 7 buffer at 30 °C, 900 rpm, anaerobic without DMSO, extracted in $CDCl_3$. ^{1}H NMR (400 MHz, $CDCl_3$) δ 7.41–7.29 (m, 5H), 5.10 (d, *J* = 4.2 Hz, 1H), 4.30 (d, *J* = 4.2 Hz, 1H), 2.09 (s, 3H). ^{13}C NMR (101 MHz, $CDCl_3$) δ 207.0, 137.9, 129.0, 128.7, 127.3, 80.1, 25.2.

(b)

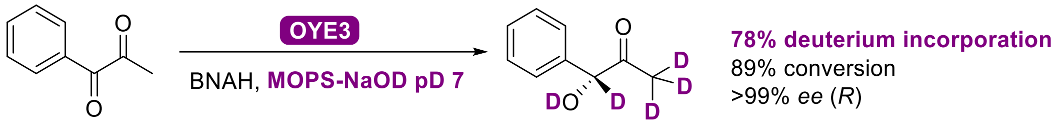

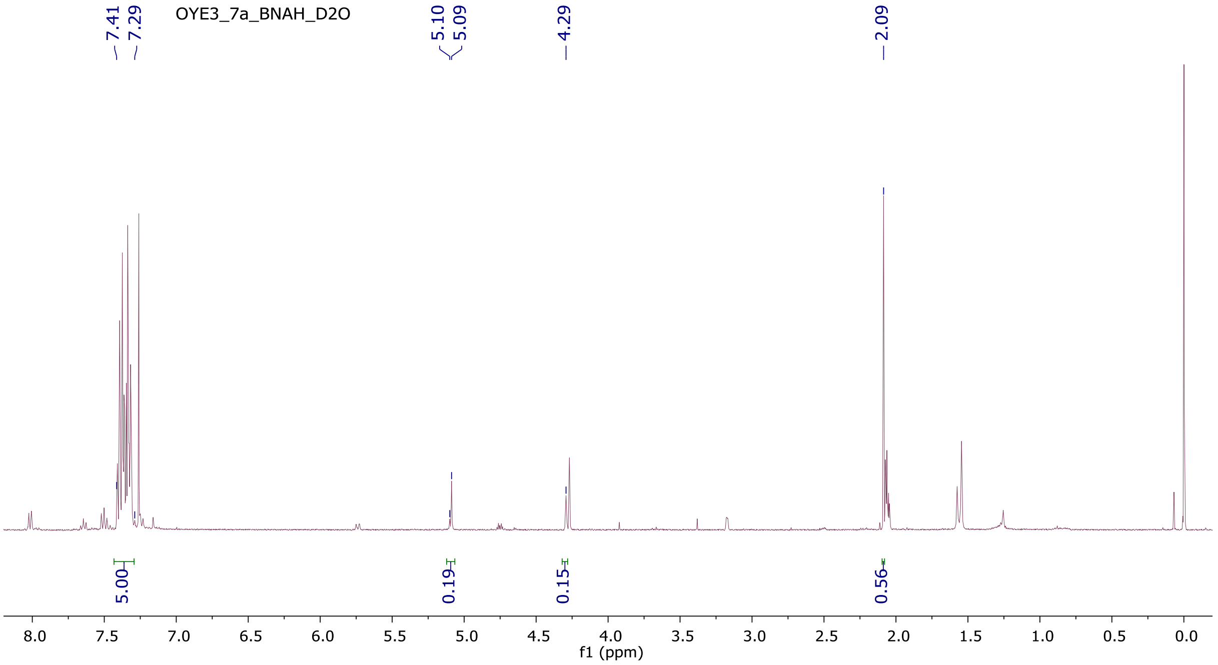

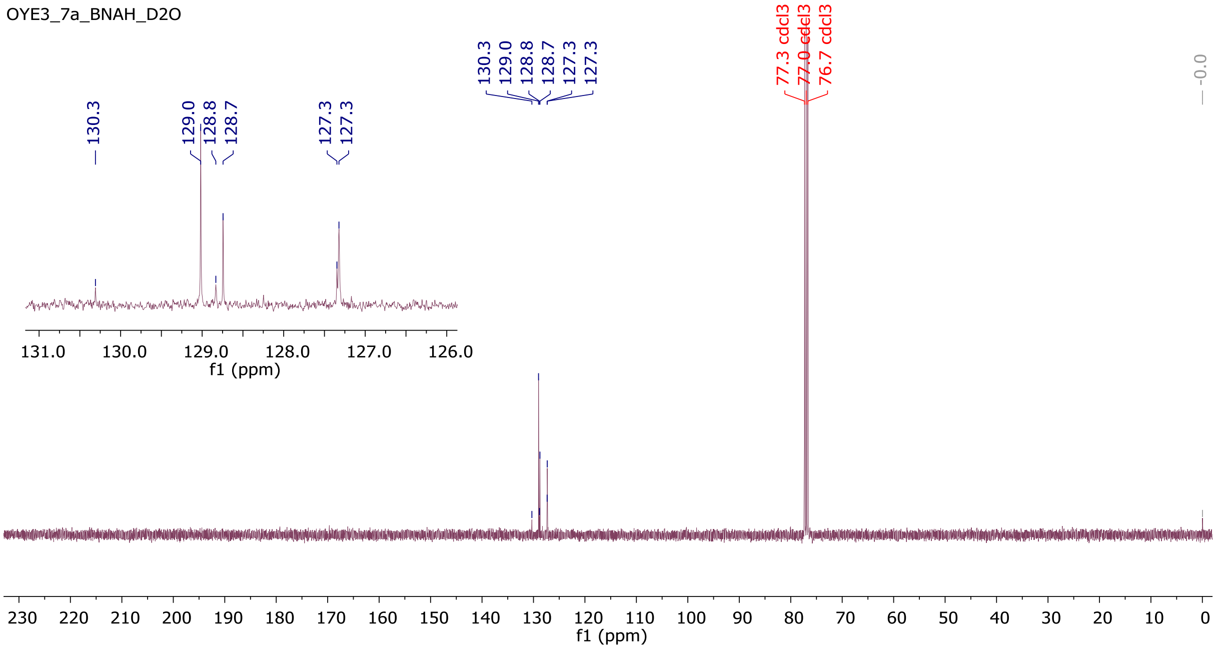

Figure S28. ^{1}H NMR spectrum of an OYE3-catalysed (60 µM) reaction with 30 mM 1-phenyl-1,2-propanedione **7a** with BNAH over 4.5 h in deuterated 50 mM MOPS-NaOD pD 7 buffer at 30 °C, 900 rpm, anaerobic without DMSO, extracted in $CDCl_3$. ^{1}H NMR (400 MHz, $CDCl_3$) δ 7.42–7.30 (m, 5H), 5.09 (s, 0.2H), 4.29 (m, 0.2H), 2.09 (s, 0.6H). ^{13}C NMR (101 MHz, $CDCl_3$) δ 130.3, 129.0, 128.8, 128.7, 127.3, 127.3.

(c)

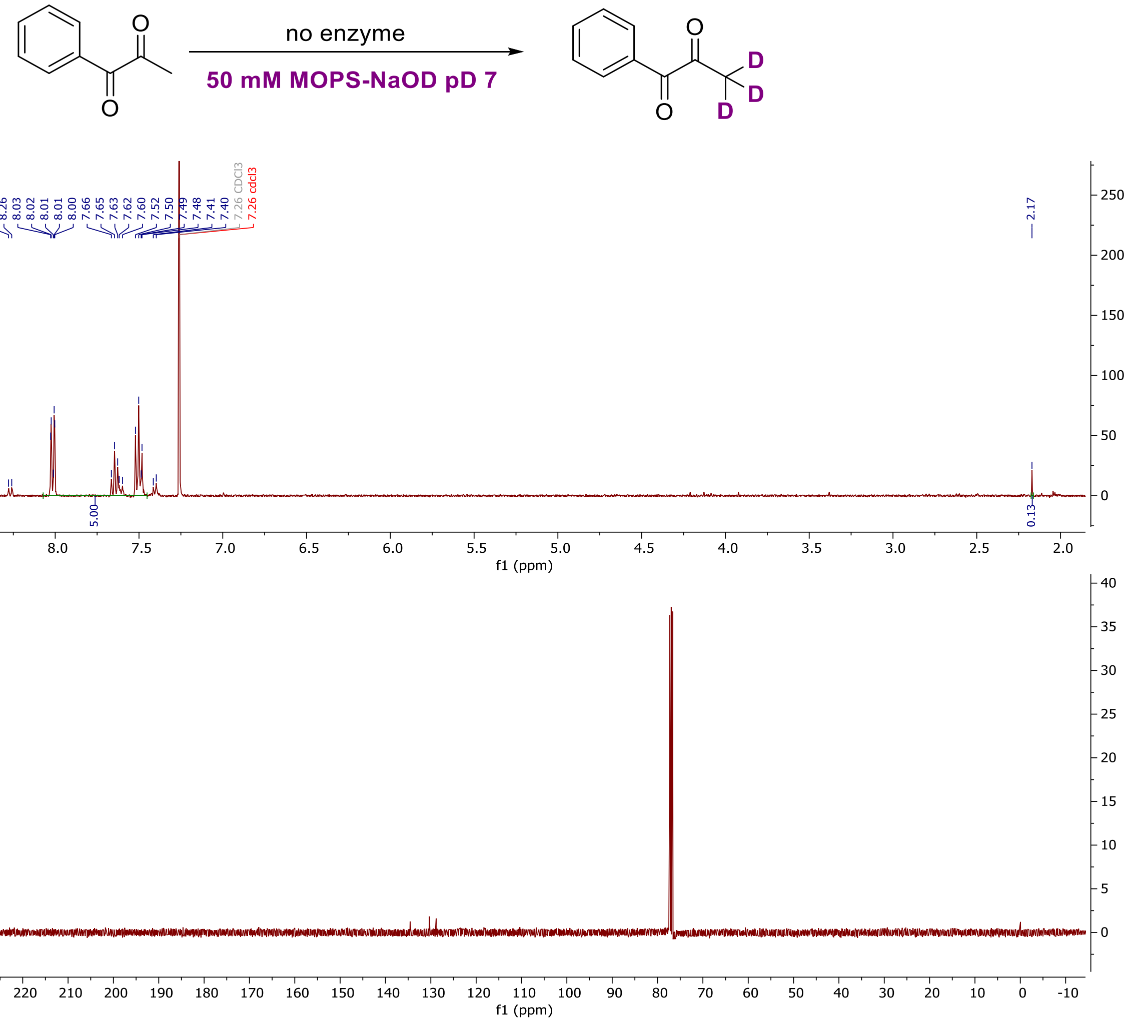

Figure S29. ^{1}H NMR spectrum of 30 mM 1-phenyl-1,2-propanedione **7a** after 4.5 h in deuterated 50 mM MOPS-NaOD pD 7 buffer at 30 °C, 900 rpm, under anaerobic conditions, without DMSO, extracted in $CDCl_3$.

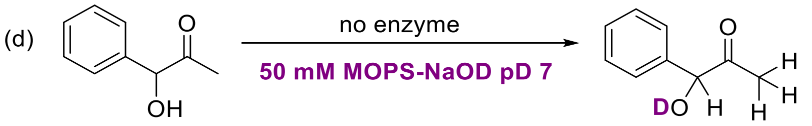

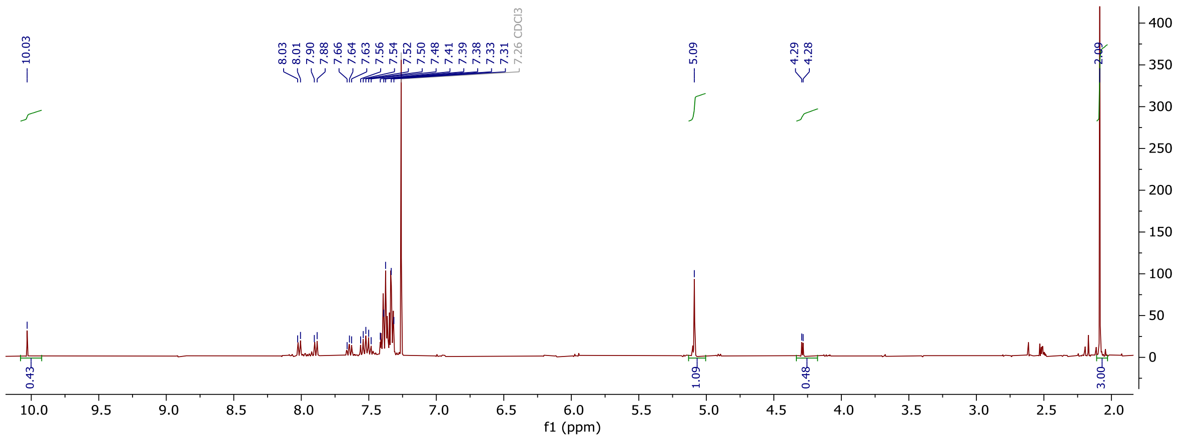

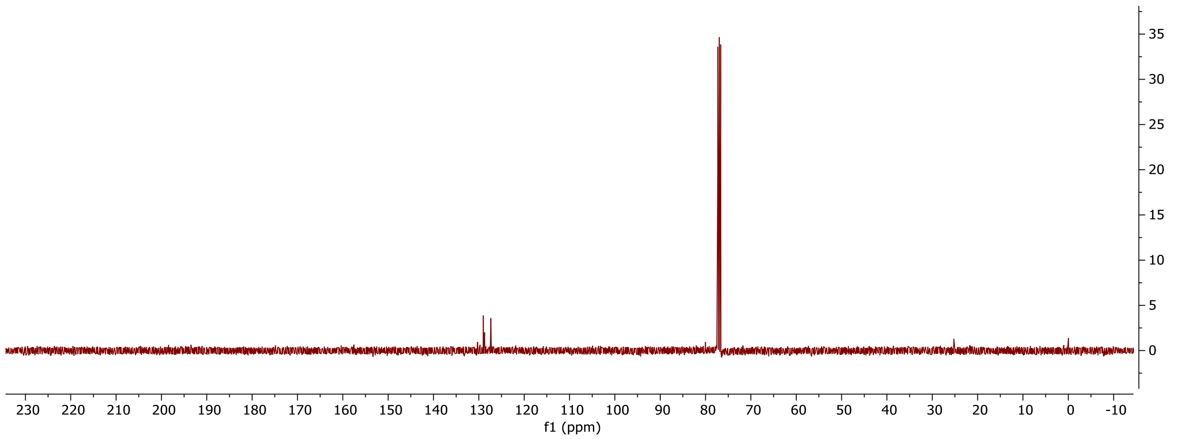

Figure S30. ^{1}H NMR spectrum of 30 mM 1-hydroxy-1-phenylpropan-2-one **7b**/**7c** after 4.5 h in deuterated 50 mM MOPS-NaOD pD 7 buffer at 30 °C, 900 rpm, under anaerobic conditions, without DMSO, extracted in $CDCl_3$.

(e)

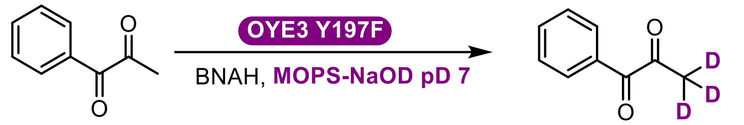

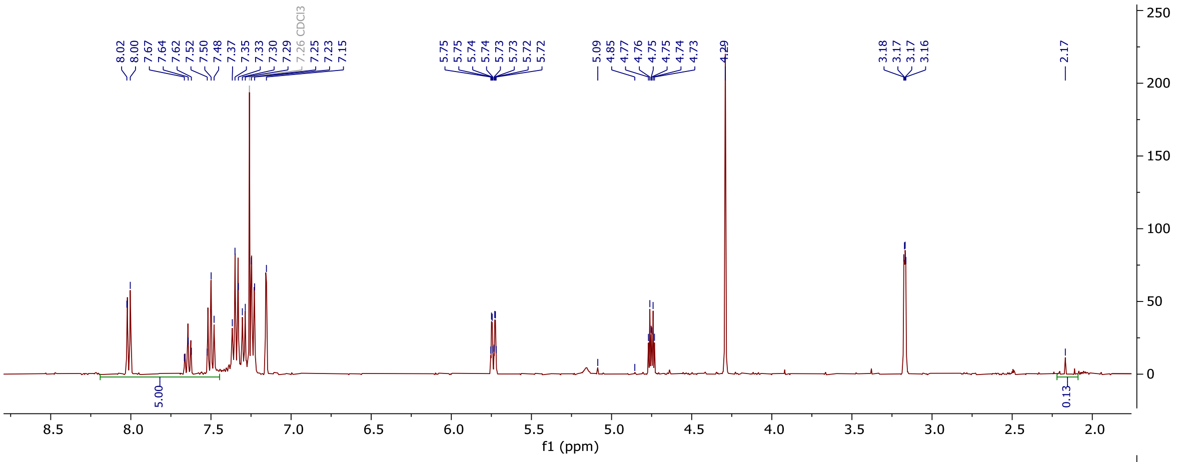

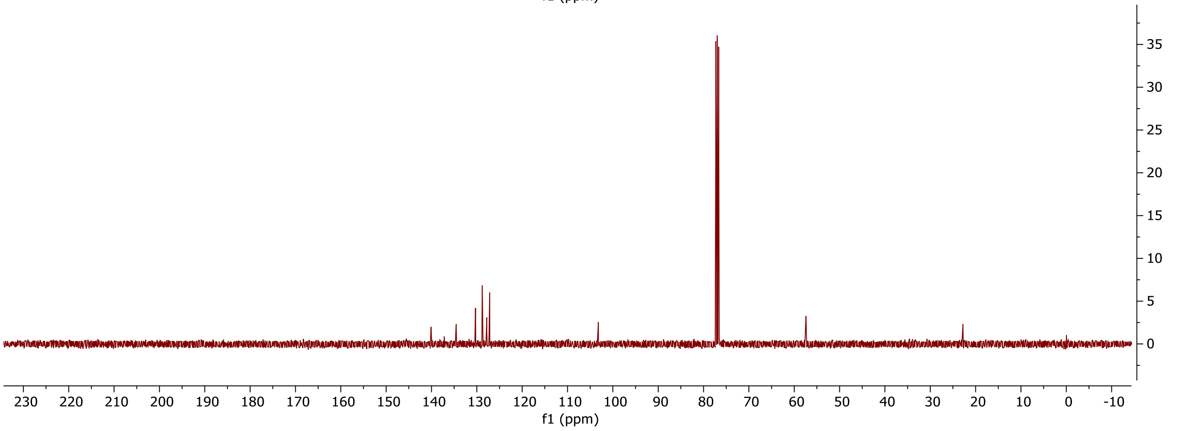

Figure S31. ^{1}H NMR spectrum of a 30 mM 1-phenyl-1,2-propanedione **7a** over 4.5 h in deuterated buffer 50 mM MOPS-NaOD pD 7 buffer at 30 °C, 60 µM OYE3 Y197F enzyme, 30 mM BNAH, 900 rpm, anaerobic without any DMSO, extracted in $CDCl_3$. Peaks correspond to two species, BNAH (7.1-7.6, 5.7, 4.8, 4.3, 3.2 ppm) and substrate 1-phenyl-1,2-propanedione **7a** (7.5-8.1, 2.2).

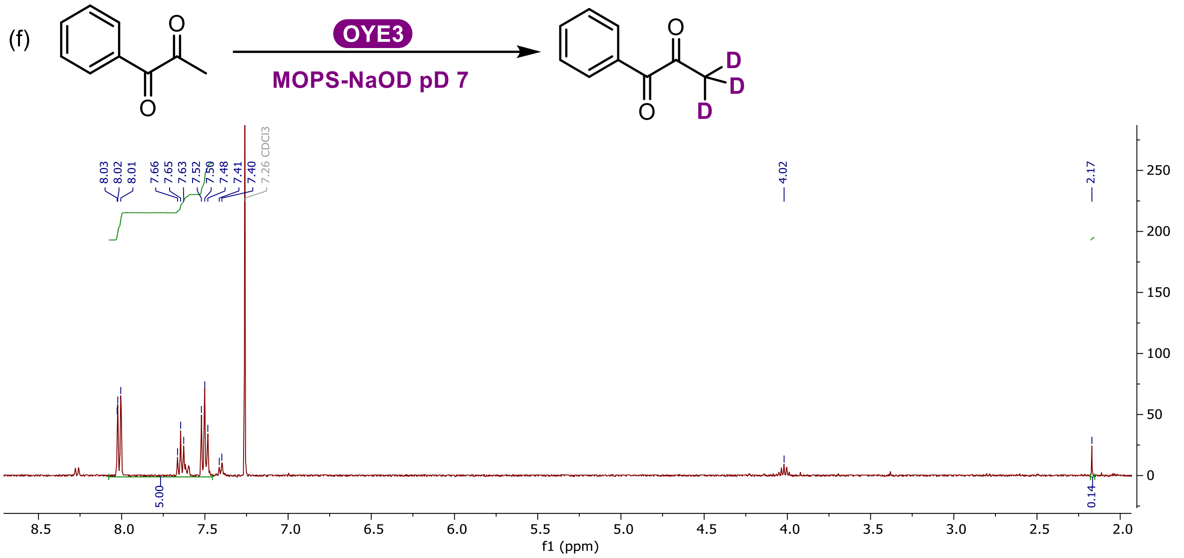

Figure S32. ^{1}H NMR spectrum of a 30 mM 1-phenyl-1,2-propanedione **7a** over 4.5 h in deuterated buffer 50 mM MOPS-NaOD pD 7 buffer at 30 °C, 60 µM OYE3, 900 rpm, anaerobic without DMSO, extracted in $CDCl_3$. The methyl group protons exchanged with deuterium.

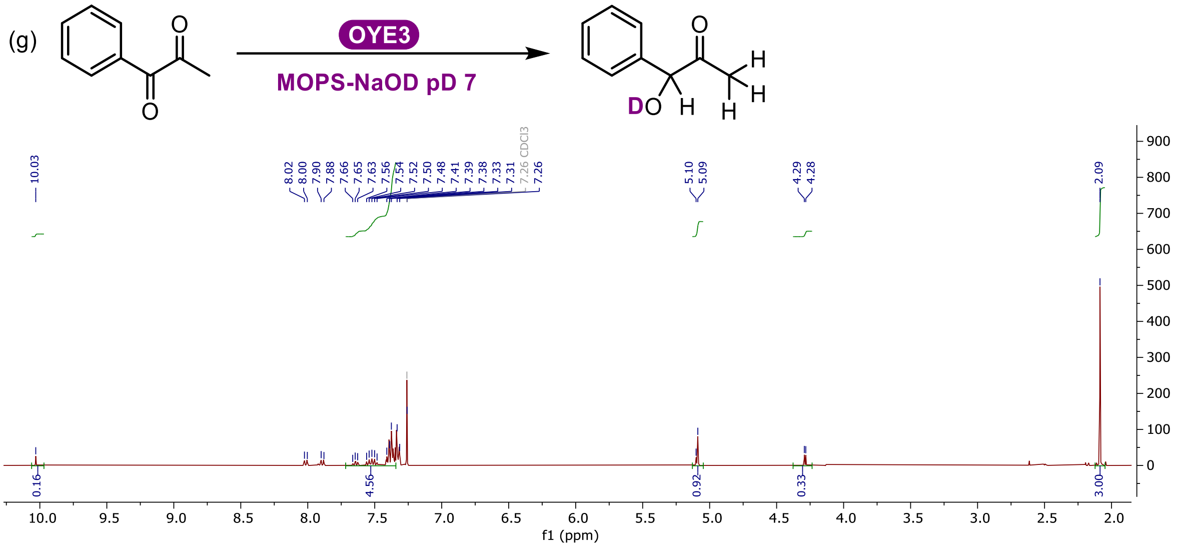

Figure S33. ^{1}H NMR spectrum of a 30 mM 1-hydroxy-1-phenylpropan-2-one **7b**/**7c** over 4.5 h in deuterated buffer 50 mM MOPS-NaOD pD 7 buffer at 30 °C, 60 µM OYE3, 900 rpm, anaerobic without any DMSO, extracted in $CDCl_3$. No deuterium exchange was observed.

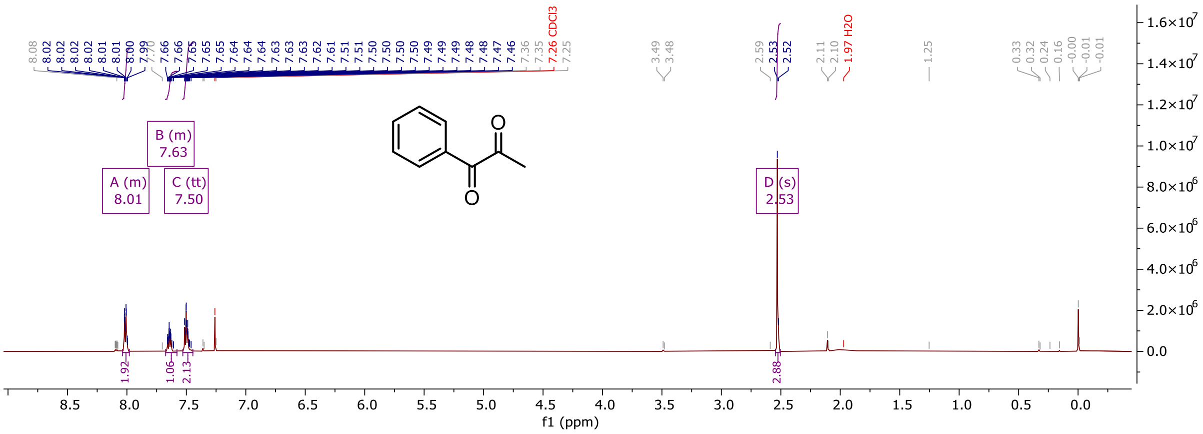

Figure S34. ^{1}H NMR spectrum of a 30 mM 1-phenyl-1,2-propanedione **7a** commercial standard.

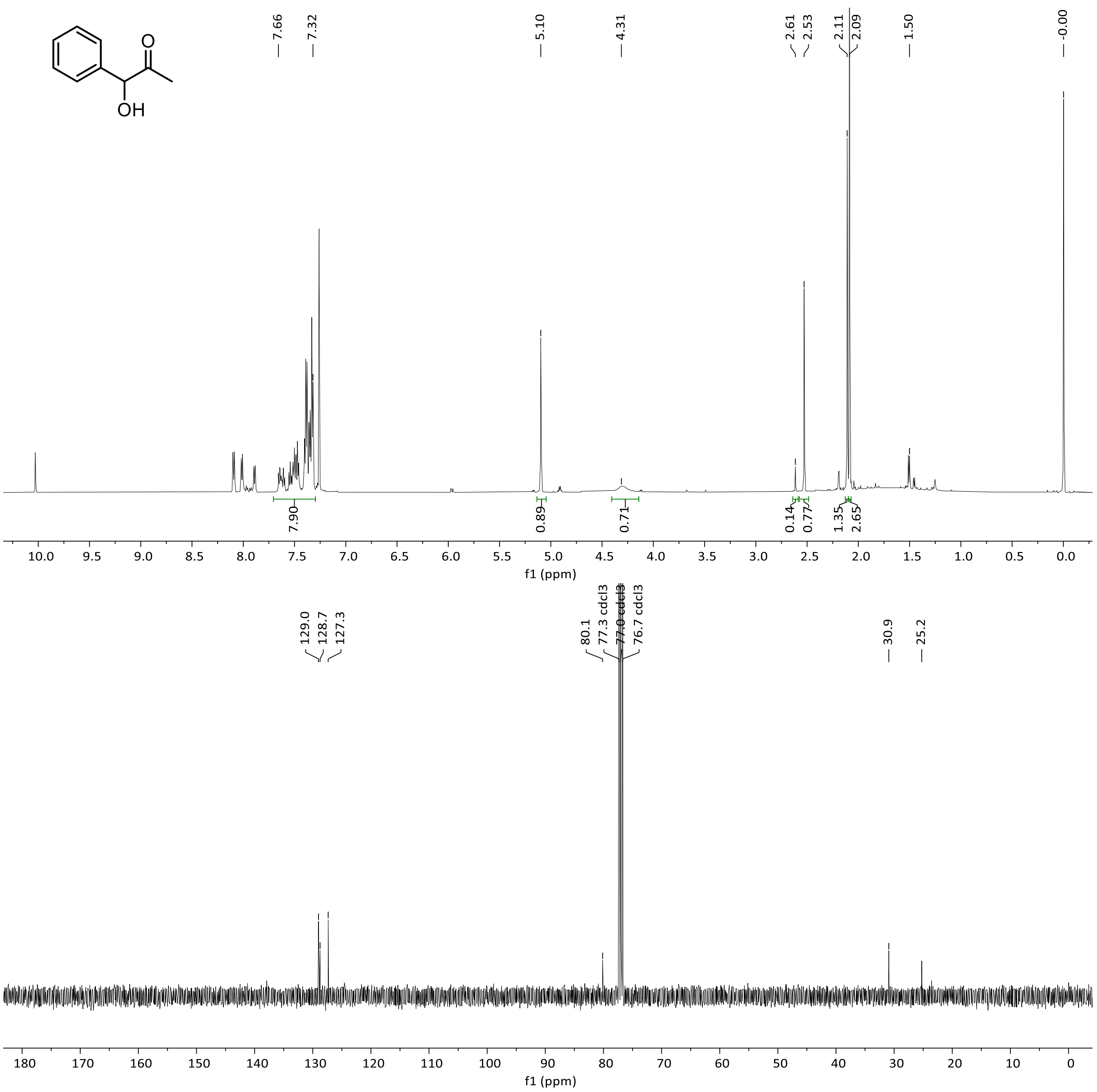

Figure S35. ^{1}H and ^{13}C NMR spectra of a 30 mM PAC **7b** commercial standard.

Preparative scale 1-phenyl-1,2-propanedione 7a

0.776 mmol (31 mM) of 1-phenyl-1,2-propanedione **7a** was placed in a 50 mL Greiner tube with OYE3 (30 µM) and 1.1 eq BNAH in 50 mM MOPS-NaOH pH 7. The reaction was run at 30 °C, 900 rpm without cosolvent and performed anaerobically in a Coy chamber and in the dark (covered in aluminium foil). After 6 h the mixture was extracted three times with 20 mL Et_2O and dried with $MgSO_4$ followed by filtration through a cotton plug that turned pink. Solvent was evaporated (600 mbar, 30-35 °C) and vacuum dried at 40 mbar for 1 h. The crude product, dark reddish brown, had a mass of 154.6 mg, and ^{1}H NMR showed 98% conversion (not shown), while the HPLC showed 99.4% conversion and an *ee* of 97.3% for the (*R*)-enantiomer (**Figure S36**). The crude product was purified by preparative TLC (Sigma Aldrich SiO2-60 F_{254}) using solvent pentane-Et2O(85:15) with two passes. The product band was scraped off and mixed with 30 mL of CH_2Cl_2 and stirred for 10 min until product fully dissolved then filtered over a cotton plug. CH_2Cl_2 was removed by rotary evaporation (300 mbar, 25 °C) then vacuum dried at 40 mbar for 30 min. Purified product amounted to 37.9 mg or 0.252 mmol and verified with ^{1}H NMR (**Figure S37**) giving an isolated yield of 33%. The comparison TLC of standard product showed multiple band separations which suggests the product is unstable on silica, and could then account for the low yield.

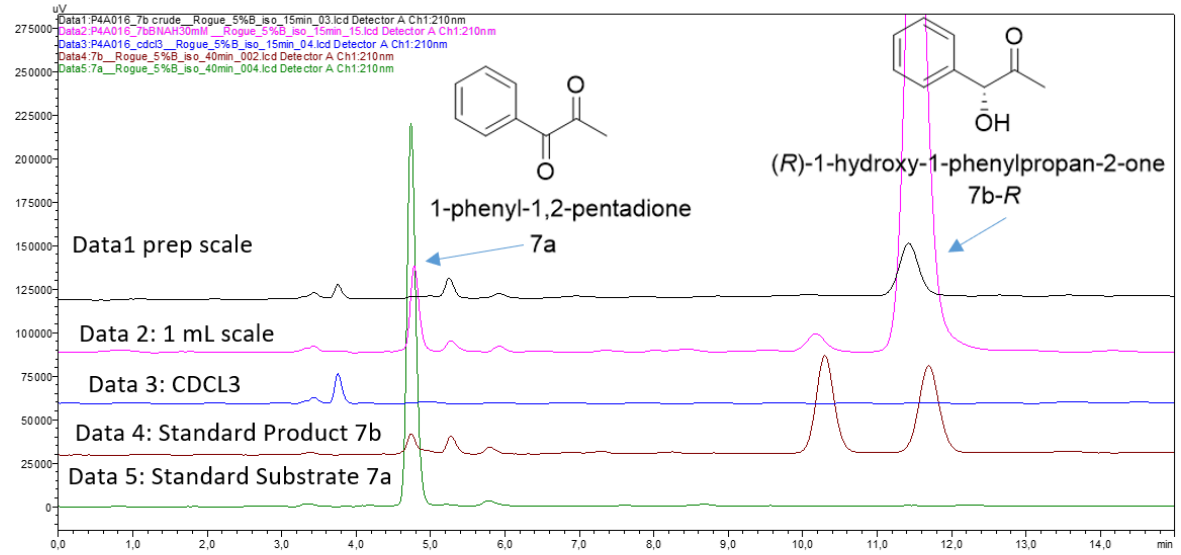

Figure S36. Chiral HPLC chromatogram of preparative scale bioconversion of 1-phenyl-1,2-propanedione **7a**. **Data1**: anaerobic reaction with OYE3 (30 µM) for 6 h with 115 mg **7a** (30 mM), 1.1 eq BNAH, 50 mM MOPS-NaOH pH 7, 30 °C, 900 rpm, no cosolvent, 25 mL volume in 50 mL Greiner tube. **Data2**: anaerobic reaction with OYE3 (20 µM) for 6 h with 30 mM **7a**, 1.1 eq BNAH, 50 mM MOPS-NaOH pH 7, 30 °C, 900 rpm, no cosolvent, 1 mL volume. **Data3:** extracted in $CDCl_3$. **Data4:** commercial standard **7b**, **Data5:** commercial standard **7a**. Normal phase HPLC, column **E** CHIRALCEL OD, 95:5 heptane:IPA at 30 °C, 210 nm.

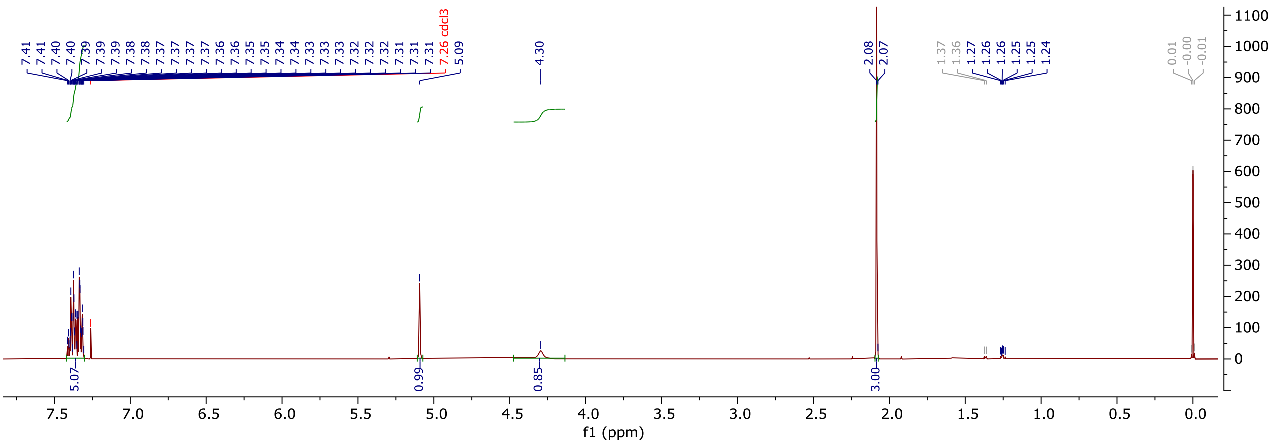

Figure S37. ^{1}H NMR spectrum in $CDCl_3$of preparative scale reaction isolated product (*R*)-1-hydroxy-1-phenylpropan-2-one **7b.**

Bioconversion of phenylglyoxal 8a

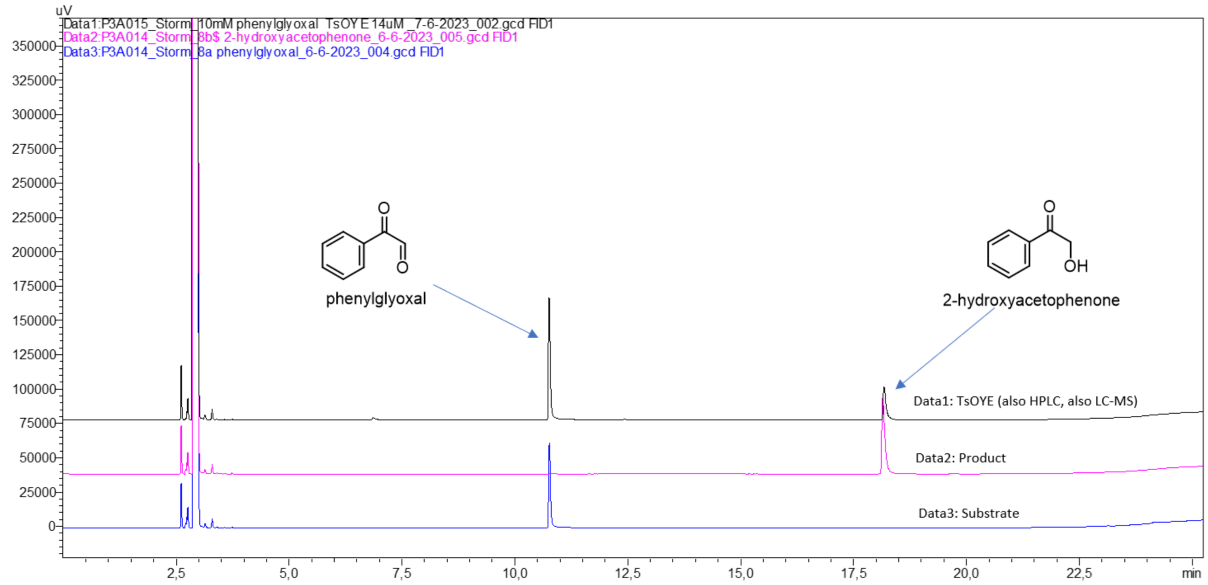

Figure S38. GC-FID chromatogram of bioconversions of phenylglyoxal **8a**.
Data1: reaction with *Ts*OYE (14 µM) for 4 h and 10 mM where also an LC-MS and HPLC sample were taken, **Data2:** commercial standard 2-hydroxyacetophenone **8c**, **Data3:** commercial standard **8a**. Column **C**, method (5).

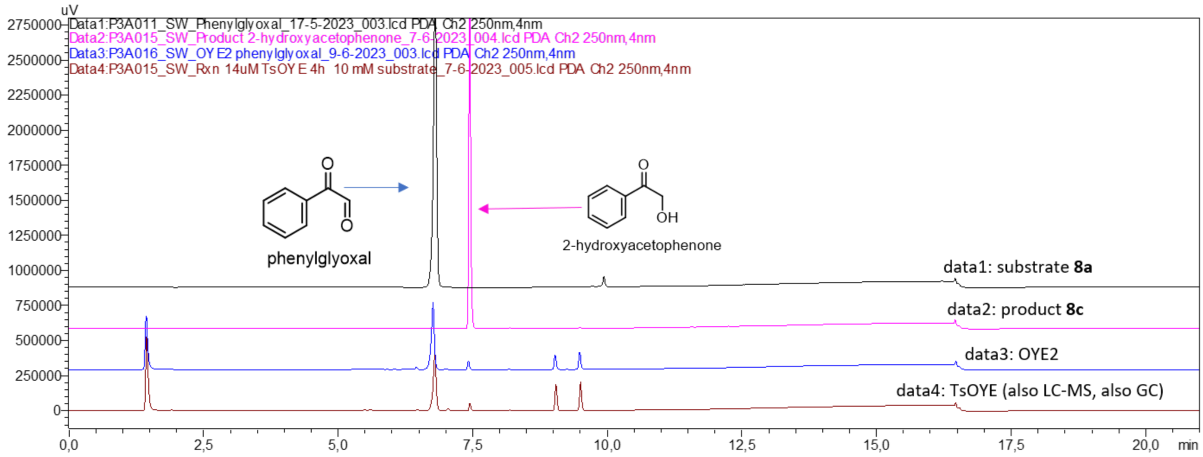

Figure S39. HPLC chromatogram of bioconversions of phenylglyoxal **8a** at 250 nm.
Data1: commercial standard **8a**, **Data2:** commercial standard 2-hydroxyacetophenone **8c**, **Data3:** reaction with OYE2 for 6 h, **Data4:** reaction with *Ts*OYE (14 µM) for 4 h and 10 mM where also an LC-MS and GC sample were taken. Column **D** ARC-18.

LC-MS extracted ion count plot of phenylglyoxal 8a

Two dilutions of the reaction with *Ts*OYE (14 µM) and 10 mM of phenylglyoxal **8a** running for 4 h were measured on LC-MS, showing a species at 271.09 m/z (**Figure S40**). This mass would correspond to 2,3-dihydroxy-1,2-diphenylbutane-1,2-dione:

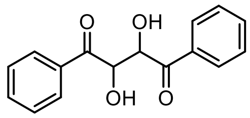

Chemical Formula: $C_{16}H_{14}O_4$
m/z: 270.09 (100.0%), 271.09 (17.5%), 272.10 (1.5%)

This species could be produced by an aldol reaction between **8b** (2-hydroxy-2-phenylacetaldehyde) and substrate **8a**. Perhaps the two unknown HPLC peaks refer to forms of this species.

NMR spectra of phenylglyoxal 8a

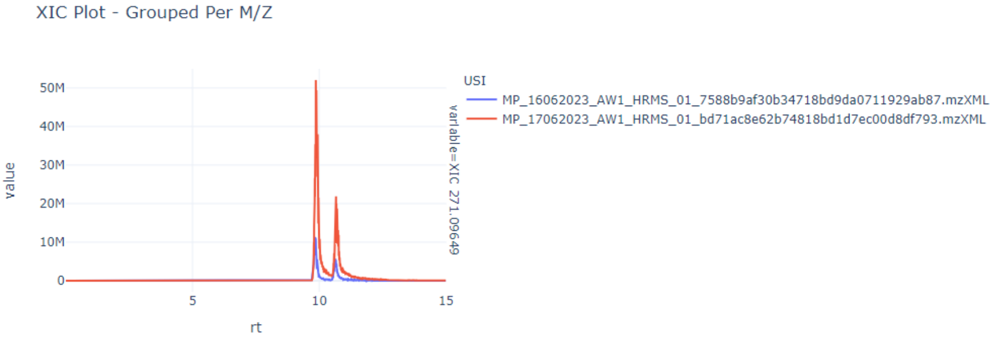

Figure S40. LC-MS XIC plot of the *Ts*OYE-catalysed reaction with phenylglyoxal **8a** after 4 h, 271.09649 m/z.

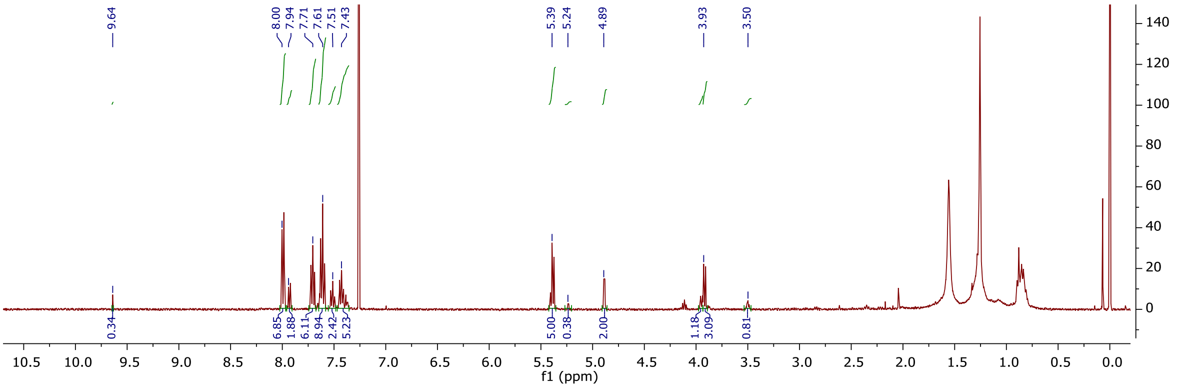

Figure S41. ^{1}H NMR spectrum of OYE3-catalysed reaction with phenylglyoxal **8a** over 5 h, extracted in $CDCl_3$. Peaks correspond to the three species observed on the HPLC: 2-hydroxyacetophenone **8c** (7.9, 7.6, 7.5, 4.9, 3.5 ppm, see **Figure S42**), two aldol products (8, 7.7, 7.6, 7.4, 5.4, 3.9 ppm). Minor peaks seen of the 2-hydroxy-2-phenylacetaldehyde **8b** (9.6, 7.0-7.6, 5.2 ppm). No substrate was observed. Peaks below 2.0 ppm are due to contamination from the plastic tube used in this case with $CDCl_3$.

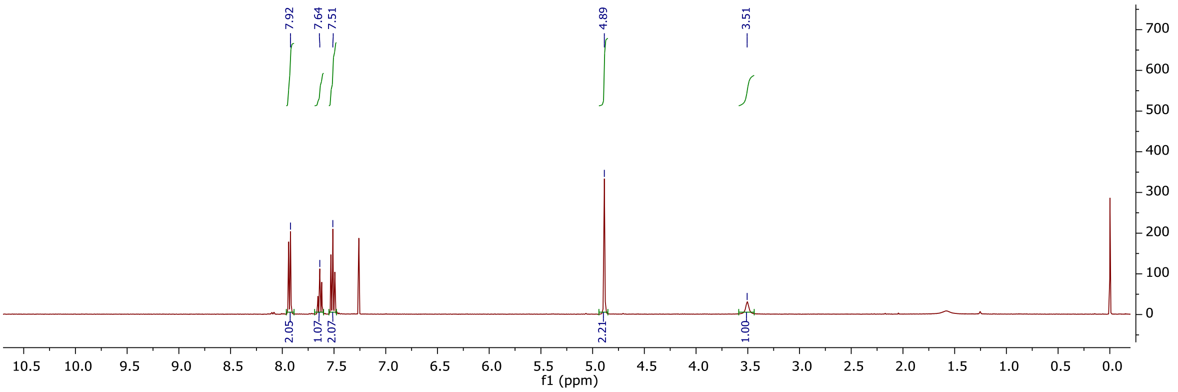

Figure S42. ^{1}H NMR spectrum of 2-hydroxyacetophenone **8c** in $CDCl_3$.

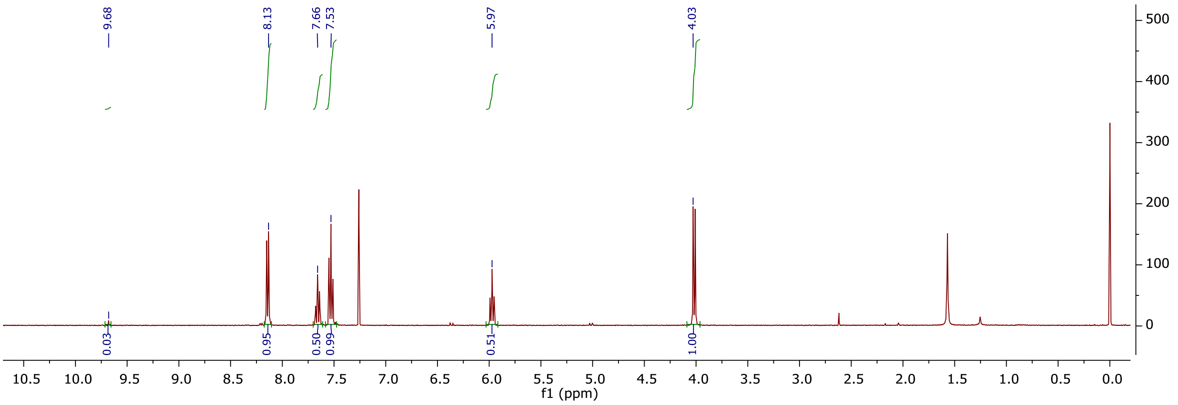

Figure S43. ^{1}H NMR spectrum of substrate phenylglyoxal **8a** in $CDCl_3$.

Bioconversion of ethylbenzoylformate 9a

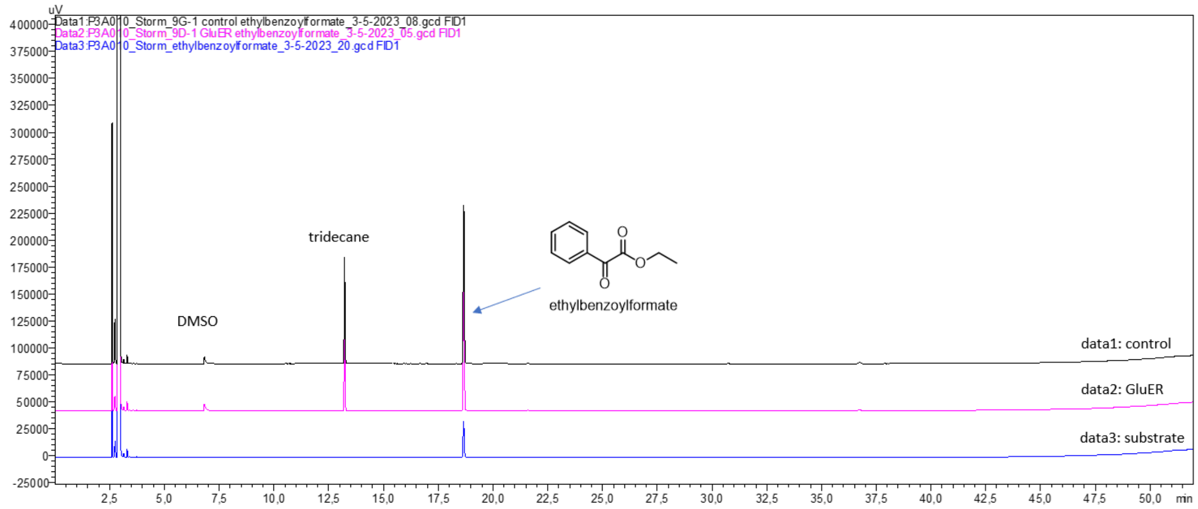

Figure S44. GC-FID chromatogram of bioconversions of ethylbenzoylformate **9a**.
Data1: control reaction without enzyme, **Data2:** reaction with GluER for 6 h, **Data3:** commercial standard **9a.** Column **C**, method (5).

Bioconversion of benzoylformic acid 10a

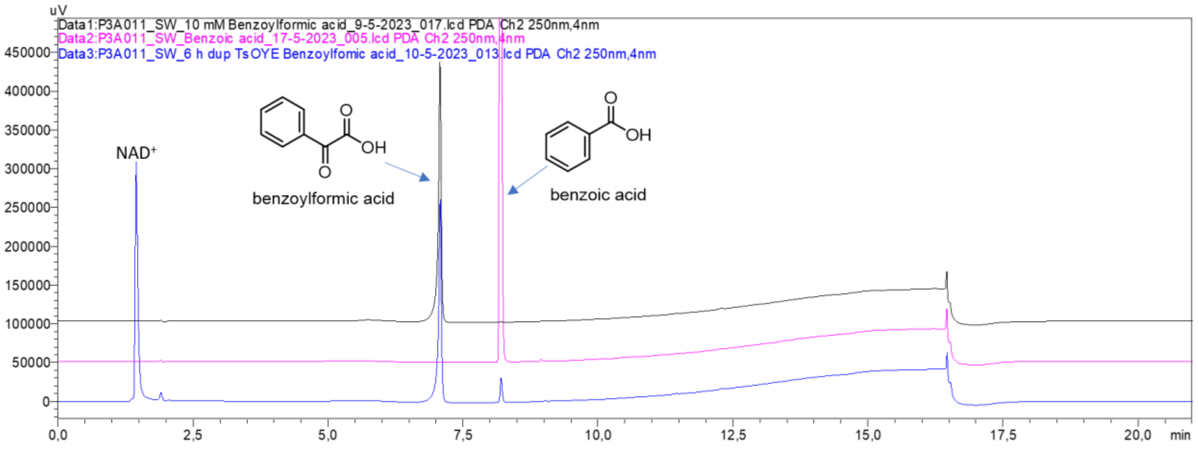

Figure S45. HPLC chromatogram of bioconversions of benzoylformic acid **10a** at 250 nm.
Data1: standard of **10a**, **Data2:** standard of benzoic acid, **Data3:** reaction with *Ts*OYE for 6h with **10a.** Column **D** ARC-18**.**

Bioconversion of 1-phenylbutan-1,2-dione 11a

OYE3 was unable to convert **11a** to **11b**.

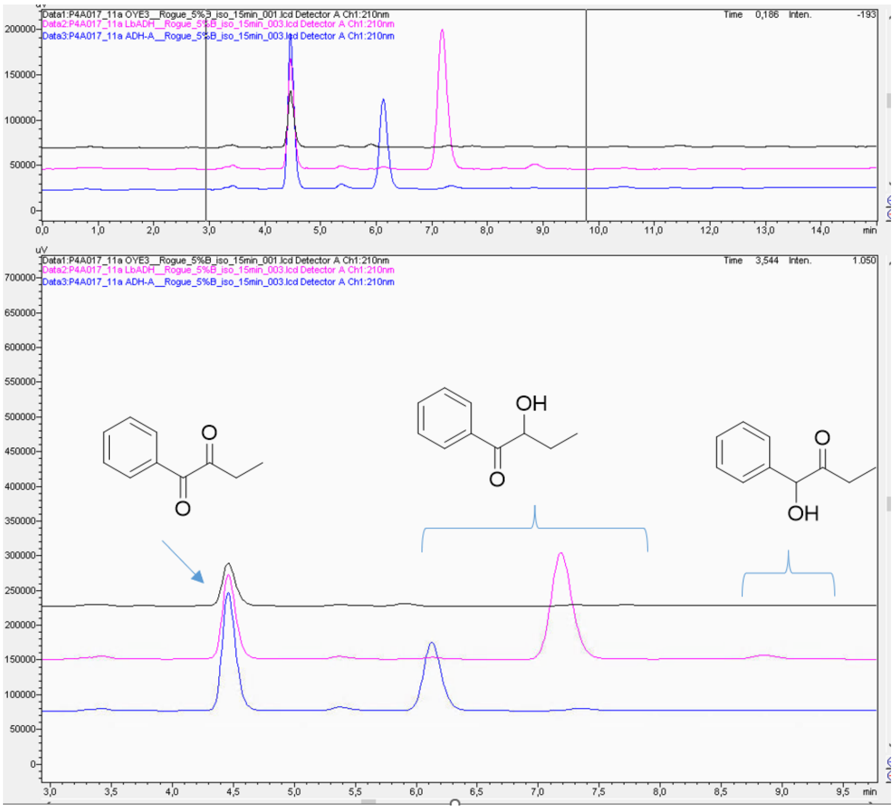

Figure S46. Chiral HPLC chromatogram of bioconversions of 1-phenylbutan-1,2-dione **11a** at 210 nm.
Data1 (black): aerobic reaction with OYE3 (5 µM) for 6h with 10 µM **11a** and 1.1 eq NADPH, 50 mM MOPS-NaOH pH 7, 30 °C, 900 rpm, 2% v/v DMSO, 0.5 mL volume. **Data2 (pink)**: aerobic reaction with *Lb*ADH (~3 U), 3.5 h 10 mM **11a**, 1 mM $MgCl_2$, 1 mM NADPH, 5% v/v IPA, 50 mM MOPS-NaOH pH 7, 30 °C, 900 rpm, 0.5 mL volume. **Data3 (blue):** aerobic reaction with ADH-A (~3 U), 10 mM **11a**, 1 mM NADH, 5% v/v IPA, 50 mM MOPS-NaOH pH 7, 30 °C, 900 rpm, 0.5 mL volume, 3.5 h, separated using normal phase HPLC on column **E** CHIRALCEL OD 95:5 heptane:IPA at 30 °C, 210 nm.

Bioconversion of benzil 12a

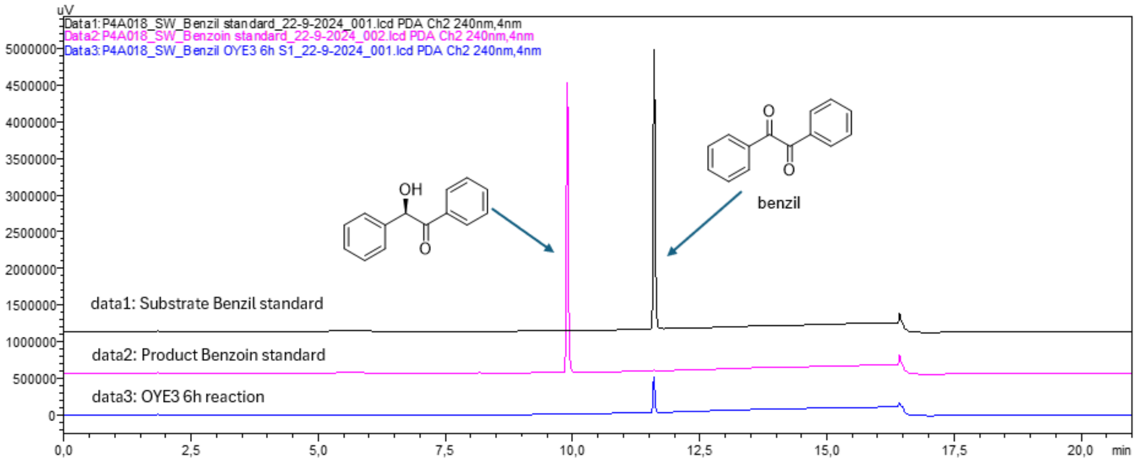

Figure S47. HPLC chromatogram of bioconversions of benzil **12a** at 240 nm.
Data1: benzil **12a** commercial standard. **Data2:** benzoin **12b** commercial standard. **Data3:** aerobic reaction with OYE3 (5 µM) for 6 h with 10 mM **12a** and 1.1 eq NADPH, 50 mM MOPS-NaOH pH 7, 30 °C, 900 rpm, 2% v/v DMSO, 0.5 mL volume. Column **D** ARC-18, method as outlined in **Table S6**.

Bioconversion of 1-(4-(trifluoromethyl)phenyl)propane-1,2-dione 13a

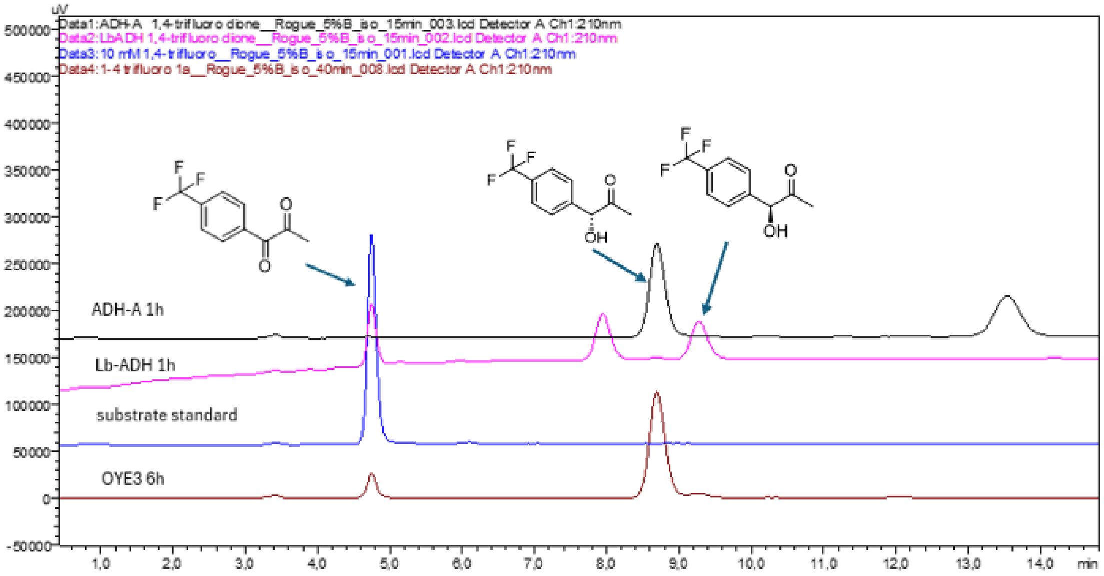

Figure S48. Chiral HPLC chromatogram of bioconversion of 1-(4-(trifluoromethyl)phenyl)propane-1,2-dione **13a**. **Data1**: reaction with ADH-A 1 h. **Data2**: reaction with *Lb*ADH 1 h. **Data3:** commercial standard **13a**. **Data4**: reaction with 5 µM OYE3, 1.1 eq NADPH, 10 mM 1-(4-(trifluoromethyl)phenyl)propane-1,2-dione **13a**, 6 h, 30 °C, 900 rpm, 2% v/v DMSO, 0.5 mL, 50 mM MOPS-NaOH pH 7. Separated using normal phase HPLC on column **E** CHIRALCEL OD 95:5 heptane:IPA at 30 °C, 210 nm. Measured conversion: 90 ± 2 %, *ee*: 99% *R*. Peak at 13.5 min is an impurity from the ADH-A reaction sample.

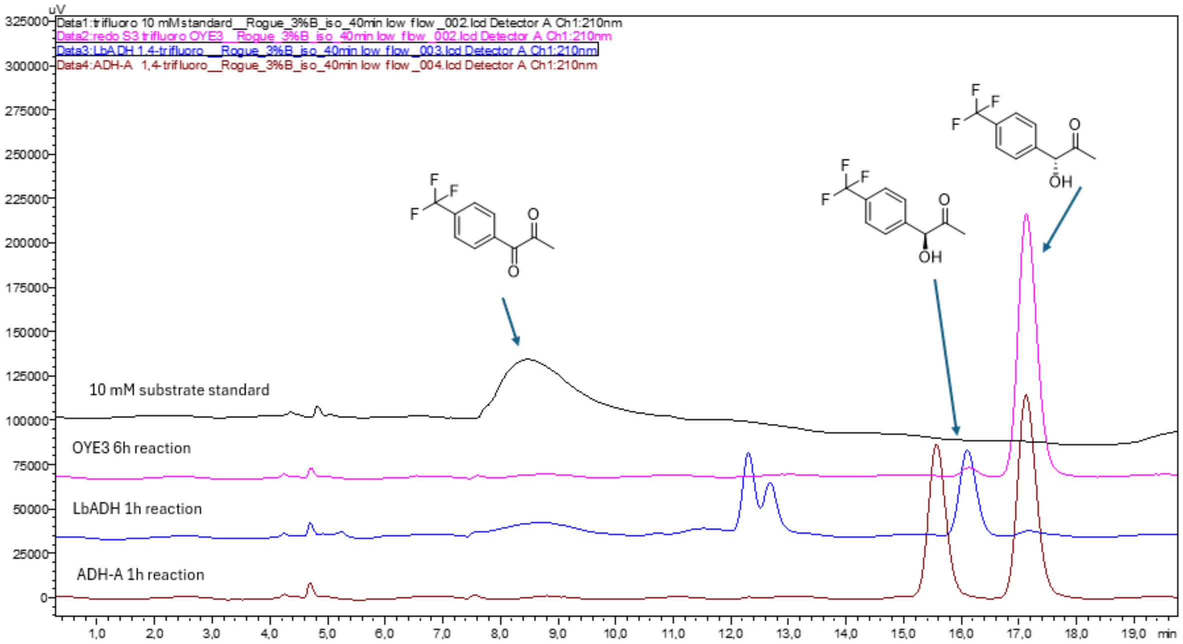

Figure S49. Chiral HPLC chromatogram of bioconversion of 1-(4-(trifluoromethyl)phenyl)propane-1,2-dione **13a.** Same data as **Figure S48**, however the method was changed to separate all peaks. We determined that the minor peak of the OYE reaction trace at 16.2 min is the other enantiomer, and no isomer (seen with *Lb*ADH at 12.2 and 12.6 min) was present. Separated using normal phase HPLC on column **F** CHIRALCEL OB-H, 97:3 heptane:IPA at 30 °C, 210 nm. Peak at 15.5 min is an impurity from the ADH-A reaction sample.

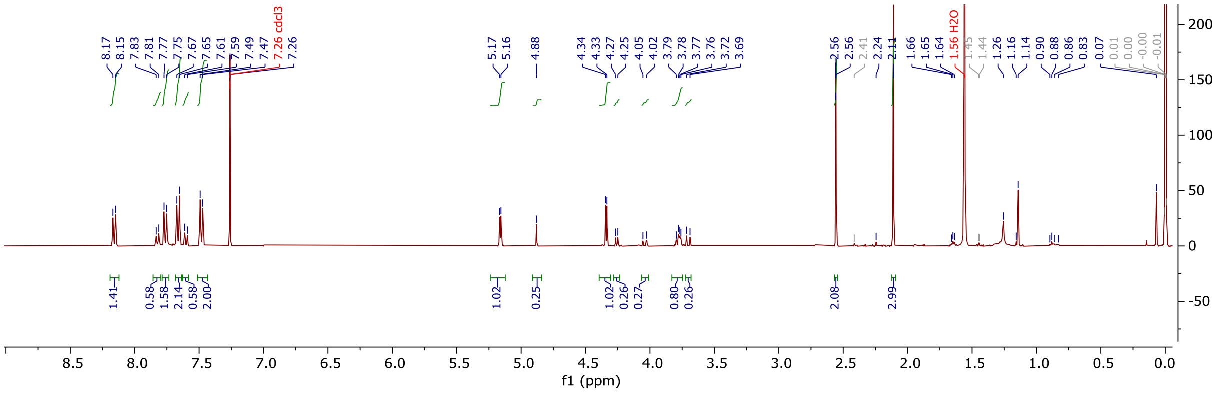

Figure S50. ^{1}H NMR spectrum of OYE3 reaction with 44 mM 1-(4-(trifluoromethyl)phenyl)propane-1,2-dione **13a**. Conditions: 60 µM OYE3, 1.1 eq NADPH, 44 mM 1-(4-(trifluoromethyl)phenyl)propane-1,2-dione **13a**, 4 h, 30 °C, 900 rpm, no cosolvent, 0.5 mL, 50 mM MOPS-NaOH pH 7. Shown here the product mixture, where 5.17 ppm is the C**H**OH of the hydroxy product.

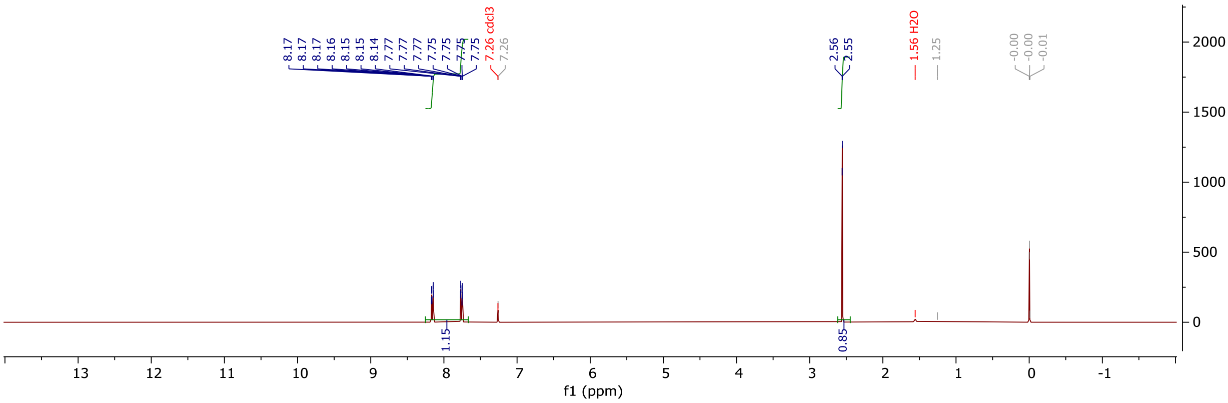

Figure S51. ^{1}H NMR spectrum of substrate standard 1-(4-(trifluoromethyl)phenyl)propane-1,2-dione **13a** for comparison**.**

Bioconversion of 1-(4-methoxyphenyl)propane-1,2-dione 14a

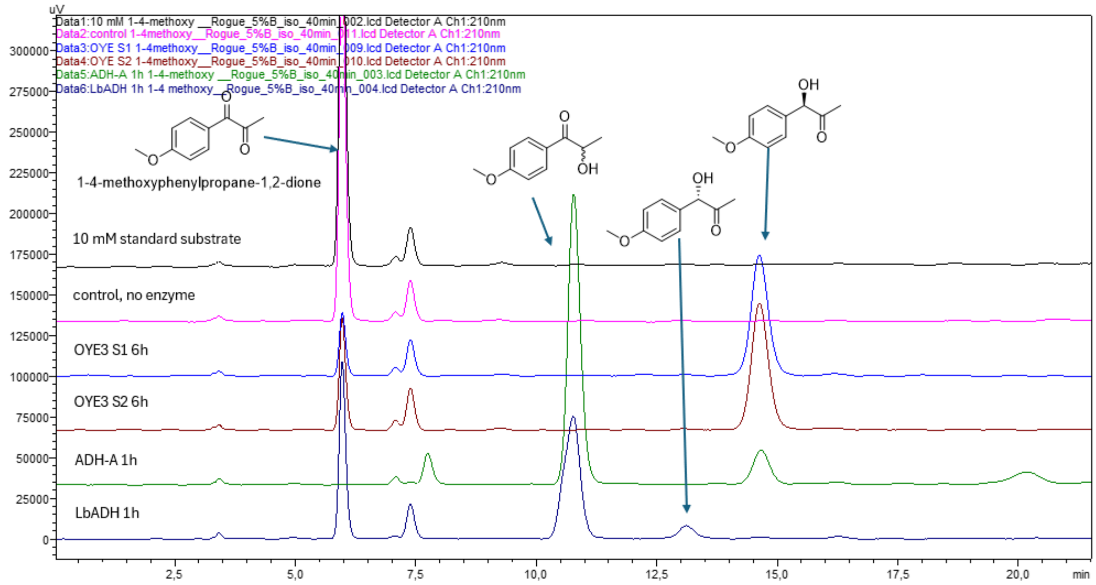

Figure S52. Chiral HPLC chromatogram of 1-(4-methoxyphenyl)propane-1,2-dione **14a** on column **E** CHIRALCEL OD, 95:5 heptane:IPA at 210 nm. **Data1**: Substrate **14a**, 10 mM extracted with 9:1 heptane:IPA. **Data2**: control reaction without enzyme. **Data3 & 4:** Duplicate aerobic reactions with OYE3 (5 µM) for 6 h with 10 mM **14a**, 1.1 eq NADPH, 50 mM MOPS-NaOH pH 7, 30 °C, 900 rpm, 0.5 mL volume. **Data5**: ADH-A 1 h. **Data6:** *Lb*ADH 1 h.

Bioconversion of 1-acetophenone 15a

No conversion of **15a** to **15b** was observed with OYE3.

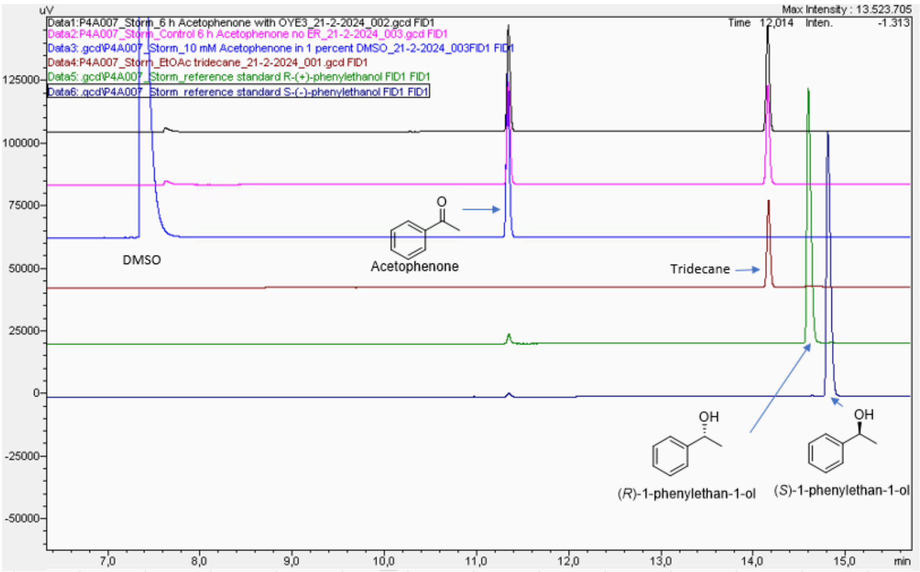

Figure S53. Chiral GC-FID chromatogram of attempted bioconversion of acetophenone **15a**.
Data1: reaction with 5 µM OYE3, 1.1 eq NADPH, 10 mM acetophenone, 2% v/v DMSO, 0.5 mL, 50 mM MOPS-NaOH pH 7, 6 h, 30 °C, 900 rpm. **Data2:** control reaction, same conditions as Data1 without OYE3. **Data3:** commercial standard **15a. Data4:** solvent and tridecane internal standard. **Data5:** commercial standard (*R*)-**15b. Data6:** commercial standard (*S*)-**15b.** Column **C**, method (5).

Proposed mechanisms

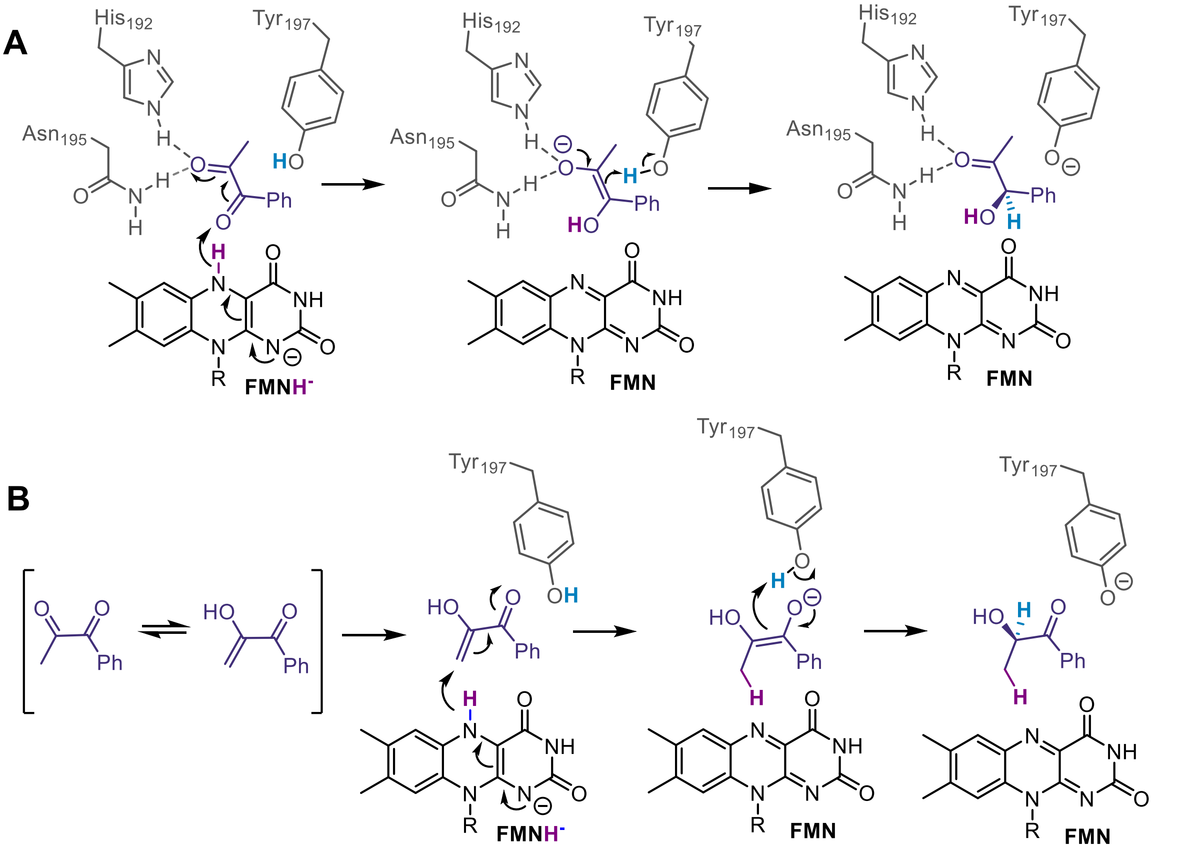

Figure S54. Proposed mechanism for the OYE monoreduction of **7a** to (*R*)-PAC **7b** and isomer **7b** (amino acid numbering of OYE2 and OYE3). **A:** hydride attack on the carbonyl oxygen and protonation at the benzylic carbon. **B:** enol formation then hydride attack and protonation at the terminal position to form **7c**, which is observed as a minor product in some cases.

Crystal structure and docking

OYE2 was crystallized by hanging drop vapor diffusion in 2 µL drops containing equal amounts of protein (OYE2, 6-8 mg/mL) and precipitant (0.1 M sodium citrate, pH 5, 16% (v/v) PEG 10 000). Crystals were soaked in 30% (w/v) glycerol before flash cryocooling in liquid nitrogen. X-ray diffraction data were collected at Diamond Light Source (UK) on beamline i03 (**Table S10**). Data was processed using autoPROC[14] with XDS[15] and scaled and merged with Aimless[16]. Molecular replacement was done using Phaser[17] OYE1 (PDB: 1OYA) as search model. The structure was refined by iterative cycles of manual building in Coot[18]and automatic refinement in Refmac5.[19] The structure was validated using programs from the CCP4 suite. Coordinates and structure factors were deposited in the Protein Data Bank (PDB) under accession code 9FH7.

Table S10. Data collection and refine statistics for OYE2.

Data collection	
X-ray source	Diamond Light Source, i03
Wavelength (Å)	0.97628
Resolution (Å)	47.23 – 1.53 (1.56 – 1.53)
Space group	I21 21 21
Unit cell parameters:	
a/b/c (Å)	99.75 / 101.27 / 160.86
α/β/γ (°)	90.0 / 90.0 / 90.0
Total reflections	567 499 (27 181)
Unique reflections	122 425 (6 095)
Completeness	99.8 (100)
R_{merge}	0.12 (1.26)
Average $I/\sigma(I)$	9.9 (1.8)
Multiplicity	4.6 (4.5)
CC(1/2)	0.997 (0.350)
**Refinement**	
R_{work}/R_{free}	0.177 / 0.203
Molecules in ASU	2
Average B factors:	
Protein	17.9
Ligands FMN/GOL	14.0 / 25.6
Solvent	27.8
RMSD:	
Bond lengths (Å)	0.012
Bond angles (°)	1.707
Ramachandran % distribution (favoured /allowed/outliers)	98 / 2 / 0

Values of highest resolution shell given in parentheses

Flexible docking of **7a** was performed using AutoDockLGA and the AMBER03 forcefield in Yasara Structure, with OYE2 (PDB: 9FH7:A), OYE3 (PDB: 5V4V:A) and *Ts*OYE (PDB: 3HF3:A). Local dockings (100 each) were performed above the FMN cofactor without result clustering and scored based on binding energy.

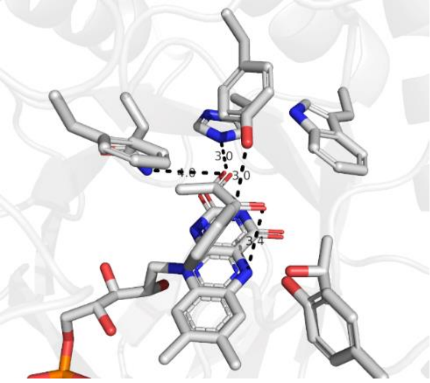

Figure S55. OYE3 docking of **7a**. β-carbonyl hydrogen bonded to His/Asn pair. Distances shown as dashed lines in Å.

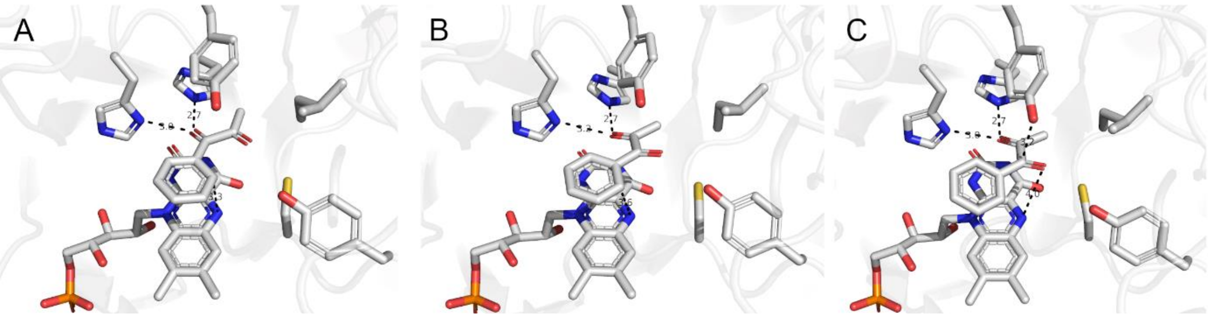

Figure S56. *Ts*OYE docking of **7a**. **A:** α-carbonyl hydrogen bonded to His pair with ortho-carbon of aromatic ring above N5 of FMN. **B:** β-carbonyl hydrogen bonded to His pair with *ortho*-carbon of aromatic ring above N5 of FMN. **C:** β-carbon hydrogen bonded to His pair with α-carbonyl oxygen closer to N5 of FMN.

References

(1) Geddes, A.; Paul, C. E.; Hay, S.; Hollmann, F.; Scrutton, N. S. Donor–Acceptor Distance Sampling Enhances the Performance of “Better than Nature” Nicotinamide Coenzyme Biomimetics. *J. Am. Chem. Soc.* **2016**, *138*, 11089–11092.

(2) Fu, H.; Lam, H.; Emmanuel, M. A.; Kim, J. H.; Sandoval, B. A.; Hyster, T. K. Ground-State Electron Transfer as an Initiation Mechanism for Biocatalytic C–C Bond Forming Reactions. *J. Am. Chem. Soc.* **2021**, *143*, 9622–9629.

(3) Salmeen, I.; Palmer, G. Electron Paramagnetic Resonance of Beef-Heart Ferricytochrome C. *J. Chem. Phys.* **1968**, *48*, 2049–2052.

(4) Lundin, A.; Aasa, R. A Simple Device to Maintain Temperatures in the Range 4.2–100 K for EPR Measurements. *J. Magn. Reson.* **1972**, *8*, 70–73.

(5) Matsumoto, N.; Shimosaka, T. Low-Temperature Electronic Paramagnetic Resonance Measurements of TEMPO and 4-Hydroxy-TEMPO Benzoate for Purity Analyses by the Effective Magnetic-Moment Method. *Anal. Sci.* **2017**, *33*, 1059–1065.

(6) Windle, J. J. Applications of Electron Paramagnetic Resonance Spectroscopy to the Study of Bio-Membranes. In *Research Instrumentation for the 21st Century*; Springer Netherlands: Dordrecht, 1988; pp 333–359.

(7) Chambers, M. C.; Maclean, B.; Burke, R.; Amodei, D.; Ruderman, D. L.; Neumann, S.; Gatto, L.; Fischer, B.; Pratt, B.; Egertson, J.; Hoff, K.; Kessner, D.; Tasman, N.; Shulman, N.; Frewen, B.; Baker, T. A.; Brusniak, M.-Y.; Paulse, C.; Creasy, D.; Flashner, L.; Kani, K.; Moulding, C.; Seymour, S. L.; Nuwaysir, L. M.; Lefebvre, B.; Kuhlmann, F.; Roark, J.; Rainer, P.; Detlev, S.; Hemenway, T.; Huhmer, A.; Langridge, J.; Connolly, B.; Chadick, T.; Holly, K.; Eckels, J.; Deutsch, E. W.; Moritz, R. L.; Katz, J. E.; Agus, D. B.; MacCoss, M.; Tabb, D. L.; Mallick, P. A Cross-Platform Toolkit for Mass Spectrometry and Proteomics. *Nat. Biotechnol.* **2012**, *30*, 918–920.

(8) Petras, D.; Phelan, V. V.; Acharya, D.; Allen, A. E.; Aron, A. T.; Bandeira, N.; Bowen, B. P.; Belle-Oudry, D.; Boecker, S.; Cummings, D. A.; Deutsch, J. M.; Fahy, E.; Garg, N.; Gregor, R.;

Handelsman, J.; Navarro-Hoyos, M.; Jarmusch, A. K.; Jarmusch, S. A.; Louie, K.; Maloney, K. N.; Marty, M. T.; Meijler, M. M.; Mizrahi, I.; Neve, R. L.; Northen, T. R.; Molina-Santiago, C.; Panitchpakdi, M.; Pullman, B.; Puri, A. W.; Schmid, R.; Subramaniam, S.; Thukral, M.; Vasquez-Castro, F.; Dorrestein, P. C.; Wang, M. GNPS Dashboard: Collaborative Exploration of Mass Spectrometry Data in the Web Browser. *Nat. Methods* **2022**, *19*, 134–136.

(9) Schrittwieser, J. H.; Coccia, F.; Kara, S.; Grischek, B.; Kroutil, W.; D'Alessandro, N.; Hollmann, F. One-Pot Combination of Enzyme and Pd Nanoparticle Catalysis for the Synthesis of Enantiomerically Pure 1,2-Amino Alcohols. *Green Chem.* **2013**, *15*, 3318–3331.

(10) Edegger, K.; Stampfer, W.; Seisser, B.; Faber, K.; Mayer, S. F.; Oehrlein, R.; Hafner, A.; Kroutil, W. Regio- and Stereoselective Reduction of Diketones and Oxidation of Diols by Biocatalytic Hydrogen Transfer. *Eur. J. Org. Chem.* **2006**, No. 8, 1904–1909.

(11) Kurina-Sanz, M.; Bisogno, F. R.; Lavandera, I.; Orden, A. A.; Gotor, V. Promiscuous Substrate Binding Explains the Enzymatic Stereo- and Regiocontrolled Synthesis of Enantiopure Hydroxy Ketones and Diols. *Adv. Synth. Catal.* **2009**, *351*, 1842–1848.

(12) Nestl, B. M.; Bodlenner, A.; Stuermer, R.; Hauer, B.; Kroutil, W.; Faber, K. Biocatalytic Racemization of Synthetically Important Functionalized α-Hydroxyketones Using Microbial Cells. *Tetrahedron: Asymmetry* **2007**, *18*, 1465–1474.

(13) Andreu, C.; del Olmo, M. Potential of Some Yeast Strains in the Stereoselective Synthesis of (R)-(−)-Phenylacetylcarbinol and (S)-(+)-Phenylacetylcarbinol and Their Reduced 1,2-Dialcohol Derivatives. *Appl. Microbiol. Biotechnol.* **2014**, *98*, 5901–5913.

(14) Vonrhein, C.; Flensburg, C.; Keller, P.; Sharff, A.; Smart, O.; Paciorek, W.; Womack, T.; Bricogne, G. Data Processing and Analysis with the *AutoPROC* Toolbox. *Acta Crystallogr. Sect. D Biol. Crystallogr.* **2011**, *67*, 293–302.

(15) Kabsch, W. *XDS*. *Acta Crystallogr. Sect. D Biol. Crystallogr.* **2010**, *66*, 125–132.

(16) Evans, P. R.; Murshudov, G. N. How Good Are My Data and What Is the Resolution? *Acta Crystallogr. Sect. D Biol. Crystallogr.* **2013**, *69*, 1204–1214.

(17) McCoy, A. J.; Grosse-Kunstleve, R. W.; Adams, P. D.; Winn, M. D.; Storoni, L. C.; Read, R. J. *Phaser* Crystallographic Software. *J. Appl. Crystallogr.* **2007**, *40*, 658–674.

(18) Emsley, P.; Lohkamp, B.; Scott, W. G.; Cowtan, K. Features and Development of *Coot*. *Acta Crystallogr. Sect. D Biol. Crystallogr.* **2010**, *66*, 486–501.

(19) Murshudov, G. N.; Skubák, P.; Lebedev, A. A.; Pannu, N. S.; Steiner, R. A.; Nicholls, R. A.; Winn, M. D.; Long, F.; Vagin, A. A. *REFMAC* 5 for the Refinement of Macromolecular Crystal Structures. *Acta Crystallogr. Sect. D Biol. Crystallogr.* **2011**, *67*, 355–367.
